# Global, regional, national burden and trends of unintentional injuries from 1990 to 2021 and projections to 2035: a systematic analysis of the Global Burden of Disease study 2021

**DOI:** 10.3389/fpubh.2025.1653491

**Published:** 2025-09-03

**Authors:** Wenpeng Qin, Xiugen Liu, Jusen Nong, Daqin Feng, Guoshen Liu, Jiaqi Xie, Weicheng Huang, Hao Liang, Linlu Yan, Haojun Tang, Fengjun Qin, Fei Huang, Kai Fu, Chang Liu, Changkai Wei, Bingning Yang, Shasha Jian, Liping Liu, Jinfeng Su, Ping Zhang, Jialing Tang, Jiao Zhuo, Qingtong Mo, Chang Liu, Yongjia Yu, Lun Liang

**Affiliations:** ^1^Department of Neurosurgery, The First Affiliated Hospital of Guangxi Medical University, Nanning, China; ^2^Department of Pediatric Surgery, The First Affiliated Hospital of Guangxi Medical University, Nanning, China; ^3^Guangxi Medical University, Nanning, China

**Keywords:** unintentional injuries, Global Burden of Disease study, age-standardised incidence rate (ASIR), age-standardised mortality rate (ASDR), age-standardised disability-adjusted life years (DALYs), prediction

## Abstract

**Background:**

Unintentional injuries, including drowning, falls, and heat-related incidents, constitute a substantial challenge to global health. The present study utilizes data from t the Global Burden of Disease (GBD) database to investigate the burden of unintentional injuries spanning the period from 1990 to 2021. It integrates these findings with future projections and advanced analytical approaches.

**Methods:**

Epidemiological data pertaining to unintentional injuries, sourced from the Global Burden of Disease (GBD) database covering the period 1990–2021, were subjected to analysis. This study centered on three core indicators: age-standardised incidence rate (ASIR), age-standardised mortality rate (ASDR), and age-standardised disability-adjusted life years (DALYs). The data were stratified by geographical region and classified in accordance with the Sociodemographic Index (SDI). The analytical approaches employed encompassed the computation of key metrics, trend evaluation, determination of relative variations, as well as the application of sophisticated methodologies for frontier analysis and projections, with all analyses conducted using R software.

**Results:**

From 1990 to 2021, the global number of new cases of unintentional injuries rose, while both mortality figures and DALYs cases associated with such injuries trended downward. Age-standardized rates for incidence, mortality, and DALYs also decreased to varying extents. Among level 3 classifications of unintentional injuries based on age-standardized rates, falls imposed the heaviest burden. A robust positive association was identified between ASIR and SDI, in contrast to the strong inverse correlations observed between SDI and both ASDR and age-standardized DALYs rate. Frontier analysis encompassing 204 countries and territories further indicated that age-standardized DALYs rates generally diminished as SDI levels climbed. Projections extending to 2035 suggest that the global downward trajectory will persist for age-standardized indicators, including ASIR, ASDR, and age-standardized DALYs rate.

**Conclusion:**

Although ASIR of unintentional injuries decreased between 1990 and 2021, and the burden of such injuries is relatively lighter in regions with a high SDI, notable disparities remain across countries. Sustained scholarly inquiry and innovative healthcare policies are imperative to further alleviate the burden imposed by unintentional injuries.

## Highlights

- While ASIR for unintentional injuries saw a reduction from 1990 to 2021, and areas with a high SDI bear a comparatively smaller burden from such injuries, significant discrepancies persist among nations. Ongoing academic investigation and pioneering health policy initiatives are crucial to further mitigate the burden brought about by unintentional injuries.
- Frontier analysis revealed superior performance in age-standardised DALYs rates within high SDI regions; nonetheless, disparities in age-standardised rates (ASR) continue to persist across certain nations.
- Projections extending to 2035 suggest that global disease metrics associated with unintentional injuries will undergo a downward trend, though the magnitude of reduction varies across different indicators.

## Introduction

Unintentional injuries are defined as external, abrupt, unplanned incidents unrelated to disease processes that result in physical damage (1). As noted by the World Health Organization (WHO), such injuries stand as the primary cause of mortality among adolescents and young adults (2, 3).

Drowning stands as a formidable global public health challenge, posing substantial mortality and morbidity burdens across the globe. Low- and middle-income countries bear over 90% of these fatalities (4, 5). Among these nations, China records the highest number of drowning-related deaths worldwide and ranks second globally in terms of mortality rate (6). As stated by the WHO, falls represent the second leading cause of unintentional injury-related deaths globally, causing roughly 684,000 fatalities each year. While falls affect all age cohorts, they are particularly common among adolescents and adults aged 60 years and above (7–9). Falls also constitute the primary cause of injury-related deaths among older adults in China (10, 11). Likewise, heat-related injuries place a significant strain on healthcare systems worldwide. Over 5 million annual deaths are associated with suboptimal temperatures, with 0.91% directly attributable to extreme heat. Between 2000 and 2019, the global excess mortality rate linked to extreme heat saw an upward trend (12–14). Heat exposure can impact mental health, disrupt thermoregulation (thereby significantly elevating basal metabolic heat production), and indirectly influence the transmission of infectious diseases (15–17).

Scholars have made strides in researching traffic and unintentional injuries. Notably, Peden, Amy E, et al. revealed that transport-related and other unintentional injuries among adolescents constituted key drivers of the associated health burden from 1990 to 2019 (3). Similarly, Behera, D. K. et al. demonstrated that road traffic injuries involving motorcyclists and pedestrians ranked as the leading causes of death in India during the same period (1990–2019), with environmental shifts, occupational hazards, behavioral risks, and metabolic risks identified as primary contributing factors (18). However, significant gaps persist in conducting granular analyses of these injuries stratified by age group, sex, year, country, and region. Furthermore, comprehensive assessments and projections regarding the global incidence, mortality, and burden of such injuries remain insufficient.

The GBD study delivers a systematic assessment of how diseases, injuries, and risk factors impact the health of global populations. It serves as a unique platform for investigating disease burdens and furnishes scientific evidence to inform public health policymaking, resource allocation, and preventive strategies (19, 20). For the first time, this research leverages the GBD 2021 database to evaluate ASIR, ASDR, and age-standardised DALYs associated with unintentional injuries from 1990 to 2021, with stratification by sex, age, and socioeconomic conditions. By integrating advanced analytical methods and forecasting techniques, it identifies trends in unintentional injuries and connects GBD 2021 estimates with longitudinal subnational socioeconomic data across countries. This approach addresses a critical evidence gap regarding the dynamic interactions between health burdens and macro-socioeconomic trends. The objective of this study is to offer insights for public health interventions and novel preventive strategies, while establishing a statistical foundation for global initiatives aimed at reducing the burden of unintentional injuries.

## Methods

### Study population and data compilation

This study drew on epidemiological datasets concerning unintentional injuries—including drowning, falls, and heat-related injuries—extracted from GBD database (1990–2021). The dataset covered 204 countries and territories, which were categorized into 21 GBD regions based on epidemiological similarities and geographic proximity. These regions were further stratified into five SDI quintiles. The core indicators analyzed included ASIR, ASDR, and age-standardised DALYs associated with unintentional injuries (21).

### Sociodemographic index (SDI)

The SDI is a composite indicator that mirrors the developmental status of a country or region. It is derived from metrics such as per capita income, educational attainment, and fertility rates, with values ranging from 0 to 1. Higher SDI values indicate superior socioeconomic and health conditions. The index classifies regions into five quintiles: low (0–0.454743), lower-middle (0.454743–0.607679), middle (0.607679–0.689504), upper-middle (0.689504–0.805129), and high (0.805129–1) (22).

### Data analysis

Analyses were conducted with R software. Initial steps involved examining the structure of the GBD dataset on unintentional injuries and calculating key indicators—including ASIR, ASDR, and age-standardised DALYs rates—at global, regional, and national levels. Trends from 1990 to 2021 were analyzed to determine changes in these indicators across different regions. Joinpoint regression was applied to identify significant temporal trends, distinguishing true trend changes from random variability (23). Pearson correlation analysis was used to assess the linear relationship between SDI values and unintentional injury indicators, offering insights into the strength and direction of these associations across regions (22). Correlations between SDI and injury metrics (ASIR, ASDR, age-standardised DALYs) were strictly descriptive, aiming to identify equity gradients rather than establish causal mechanisms.

Frontier analysis was employed to evaluate the relationship between unintentional injury burden and sociodemographic development. It defines a non-linear frontier representing the minimum achievable burden for a given SDI level. The Effective Difference (observed burden minus frontier burden) reflects unrealized health gains based on a country’s current development level (24, 25). Countries farther from the frontier exhibit larger effective differences and greater improvement potential. This frontier analysis approach enabled comparison of health outcomes among countries or regions with optimal performance at their respective SDI levels, establishing a theoretical baseline for the minimum burden of unintentional injuries. It quantified relative disparities in health outcomes across development levels and identified improvement opportunities by calculating “efficiency gaps” between current and potential burdens, adjusted for SDI.

### Prediction of trends

Future trends in unintentional injuries from 2021 to 2035 were projected via the Bayesian Age-Period-Cohort (BAPC) framework. This model accounts for age, period, and cohort effects, making it well-suited for predicting age-stratified incidence and mortality rates. It thus facilitated projection of unintentional injury trends in the coming years (26, 27). All statistical analyses and data visualizations were performed using R (version 4.4.2) and JD_GBDR (V2.37, Jingding Medical Technology Co., Ltd.). In this study, the R software package (version 4.2.3) and JD_GBDR (V2.22, Jingding Medical Technology Co., Ltd.) was used for the drawing of the figures, with a *P*-value < 0.05 considered statistically significant.

## Results

### Global trends

**Figure 1:**
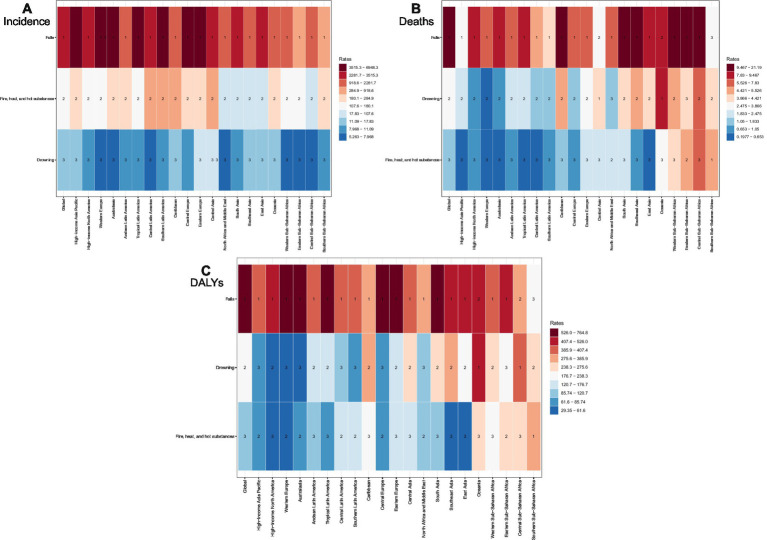
The rank of level 3 cause of unintentional injuries for age- standardized rate of incidence **(A)**, deaths **(B)** and DALYs **(C)** by regions in 2021.

Between 1990 and 2021, the global count of new unintentional injury cases rose by 13.5%, increasing from 444.43 million to 504.55 million. Conversely, ASIR fell by 22.7%, dropping from 8,268.4 per 100,000 population to 6,389.7 per 100,000 population. Mortality cases saw a substantial absolute decrease of 32.3%, alongside a notable 39.7% reduction in ASDR. Similarly, global DALYs cases associated with unintentional injuries declined by 45.5%, with their ASR showing a comparable 45.9% reduction (Tables 1–3). Among level 3 classifications of unintentional injuries based on age-standardized rates, falls ranked as the leading cause of incidence in 2021 (Figure 1A). They also imposed the heaviest burden of death and disability (Figures 1B,C).

**Table 1:** Volume and ASR of global unintentional injury morbidity in 1990 and 2021 and annual percentage change in age-standardized estimates between 1990 and 2021.

Characteristic	Incident cases			ASIR per 100,000			
	1990, *N* (95% UI)	2021, *N* (95% UI)	Percentage change (%)	1990, *N* (95% UI)	2021, *N* (95% UI)	Relative change of age-standardized rate from 1990 to 2021	Percentage change (%)
Global	444,434,306 (409,360,047, 480,169,775)	504,555,087 (472,014,395, 539,343,468)	13.527 (11.009, 15.969)	8268.41 (7652.57, 8918.02)	6389.73 (5965.92, 6828.14)	−1878.68	−22.721 (−23.735, −21.810)
Sex							
Female	169,972,174 (156,642,302, 183,857,648)	210,364,435 (196,109,440, 225,734,555)	—	6417.63 (5931.21, 6929.06)	5293.01 (4918.65, 5692.71)	−1124.62	—
Male	274,462,132 (252,347,839, 296,691,305)	294,190,651 (274,596,605, 314,934,141)	—	10010.20 (9236.15, 10774.61)	7425.09 (6934.76, 7930.79)	−2585.11	—
5 SDI Region							
High-middle SDI	109,715,840 (100,463,776, 119,029,214)	102,335,329 (95,017,818, 110,300,382)	−6.727 (−9.358, –4.169)	10217.78 (9360.06, 11081.91)	8,187 (7536.98, 8827.4)	−2030.78	−19.875 (−20.989, –18.648)
High SDI	119,902,824 (110,386,727, 129,816,627)	123,879,180 (115,694,720, 132,174,585)	3.316 (0.786, 5.684)	13832.17 (12644.37, 15034.46)	11254.92 (10355.68, 12174.21)	−2577.25	−18.632 (−19.629, –17.503)
Low-middle SDI	76,739,859 (70,887,747, 83,195,338)	98,154,235 (91,769,392, 104,772,654)	27.905 (24.499, 31.353)	6464.21 (6013.17, 6920.29)	5135.58 (4812.22, 5462.38)	−1328.63	−20.554 (−21.801, –19.229)
Low SDI	28,337,111 (26,111,358, 30,467,691)	49,645,593 (46,221,056, 53,353,815)	75.196 (71.875, 78.742)	5414.33 (5056.5, 5767.37)	4432.27 (4165.38, 4707.18)	−982.06	−18.138 (−19.379, –16.715)
Middle SDI	109,125,994 (100,027,087, 119,566,959)	130,006,446 (120,425,400, 141,127,503)	19.134 (15.263, 22.664)	6115.93 (5630.6, 6687.17)	5364.72 (4981.07, 5816.11)	−751.21	−12.283 (−13.504, –10.943)
21 GBD Region							
East Asia	51,682,033 (46,438,727, 58,850,615)	68,236,680 (61,841,659, 76,983,344)	32.032 (24.861, 38.860)	4233.44 (3820.13, 4755.66)	4568.33 (4134.93, 5080.48)	334.89	7.911 (5.111, 10.816)
Southeast Asia	25,243,083 (23,245,547, 27,364,402)	29,088,266 (27,151,687, 31,084,416)	15.233 (12.157, 18.169)	5260.07 (4875.26, 5664.99)	4203.88 (3916.36, 4496.68)	−1056.19	−20.079 (−21.319, –18.791)
Oceania	222,858 (206,173, 242,802)	478,995 (449,311, 511,719)	114.933 (107.635, 121.533)	3375.67 (3156.81, 3609.61)	3496.5 (3294.99, 3717.79)	120.83	3.580 (0.643, 6.264)
Central Asia	8,038,563 (7,353,048, 8,708,280)	8,510,358 (7,839,134, 9,181,951)	5.869 (3.669, 8.134)	10829.25 (9950.85, 11669.05)	8818.49 (8116.25, 9530.91)	−2010.76	−18.568 (−19.908, –17.300)
Central Europe	25,226,007 (22,894,997, 27,434,008)	16,886,361 (15,535,452, 18,175,364)	−33.060 (−34.726, −31.368)	20727.47 (18747.04, 22630.34)	16045.75 (14405.61, 17606.18)	−4681.72	−22.587 (−23.978, –21.189)
Eastern Europe	37,762,730 (34,473,185, 41,095,888)	25,234,371 (23,348,762, 27,155,955)	−33.177 (−35.539, –30.873)	17353.59 (15758.04, 18923.07)	13152.53 (12127.5, 14162.24)	−4201.06	−24.209 (−26.169, –22.017)
High-income Asia Pacific	25,228,641 (23,157,285, 27,424,584)	18,470,693 (17,109,119, 19,904,432)	−26.787 (−28.681, –24.754)	14788.02 (13504.81, 16114.11)	10937.27 (9898.22, 12005.97)	−3850.75	−26.040 (−27.403, –24.751)
Australasia	5,576,649 (4,986,247, 6,153,416)	7,172,257 (6,533,758, 7,797,879)	28.612 (24.799, 32.985)	28402.88 (25290.21, 31561.93)	24965.56 (22212.63, 27668.42)	−3437.32	−12.102 (−14.547, –9.576)
Western Europe	55,351,158 (51,241,681, 59,742,520)	52,346,233 (48,855,095, 56,081,389)	−5.429 (−7.363, –3.450)	14948.86 (13681.06, 16299.04)	12581.27 (11489.63, 13778.3)	−2367.59	−15.838 (−17.373, –14.233)
Southern Latin America	9,650,463 (8,735,200, 10,489,750)	11,498,292 (10,516,892, 12,429,372)	19.148 (15.372, 23.659)	19213.49 (17416.04, 20846.63)	17685.05 (16057.66, 19158.81)	−1528.44	−7.955 (−10.597, –5.125)
High-income North America	35,383,742 (32,214,917,38,625,000)	41,659,830 (38,819,137,44,651,349)	17.737 (12.814,22.186)	12539.36 (11386.3,13732.79)	10151.29 (9386.19,10940.87)	−2388.07	−19.045 (−21.570, –16.528)
Caribbean	2,655,116 (2,430,685, 2,883,995)	3,781,088 (3,502,715, 4,033,090)	42.408 (38.440, 46.375)	7236.35 (6651.77, 7801.8)	8116.78 (7502.39, 8689.25)	880.43	12.167 (10.106,14.525)
Andean Latin America	3,076,164 (2,865,634, 3,310,590)	4,620,612 (4,298,332, 4,941,656)	50.207 (45.999, 54.003)	7560.35 (7077.44, 8067.19)	6884.05 (6407.22, 7367.44)	−676.3	−8.945 (−11.107, –7.020)
Central Latin America	21,350,688 (19,233,702, 23,537,199)	20,490,362 (18,553,241, 22,331,122)	−4.030 (−6.465, –1.281)	11756.24 (10692.58, 12774.6)	8270.36 (7455.29, 9049.3)	−3485.88	−29.651 (−30.882, –28.341)
Tropical Latin America	16,321,063 (14,786,474, 18,235,682)	16,517,453 (15,247,404, 17,956,052)	1.203 (−3.013, 5.780)	10113.96 (9180.65, 11297.93)	7325.55 (6774.79, 7985.2)	−2788.41	−27.570 (−30.028, –25.261)
North Africa and Middle East	26,509,400 (24,424,252, 28,596,423)	38,480,294 (35,893,552, 41,266,506)	45.157 (41.036, 49.200)	7323.82 (6796.37, 7840.51)	6036.4 (5638.37, 6448.48)	−1287.42	−17.578 (−19.141, –16.008)
South Asia	71,421,995 (65,844,057, 77,628,410)	96,865,950 (90,382,748, 103,702,901)	35.625 (30.888, 40.414)	6601.91 (6133.96, 7090.03)	5320.13 (4976.32, 5677.55)	−1281.78	−19.415 (−21.118, –17.657)
Central Sub-Saharan Africa	2,408,532 (2,220,242, 2,618,242)	4,884,647 (4,504,148, 5,291,755)	102.806 (96.958, 108.241)	4058.37 (3782.26, 4336.65)	3471.43 (3254.66, 3690.25)	−586.94	−14.463 (−16.226, –12.785)
Eastern Sub-Saharan Africa	9,885,642 (9,086,212, 10,726,194)	17,217,869 (15,898,640, 18,704,206)	74.170 (70.835, 77.617)	4785.27 (4440.77, 5110.55)	3890.55 (3635.69, 4151.86)	−894.72	−18.697 (−19.795, –17.554)
Southern Sub-Saharan Africa	2,394,667 (2,195,795, 2,606,838)	2,769,938 (2,580,153, 2,980,222)	15.671 (12.629, 18.736)	4273.11 (3958.35, 4598.9)	3364.11 (3138.58, 3613.67)	−909	−21.273 (−22.707, –19.805)
Western Sub-Saharan Africa	9,045,112 (8,306,646, 9,801,214)	19,344,540 (17,890,280, 20,971,445)	113.867 (109.428, 118.331)	4282.44 (3962.81, 4579.06)	3760.87 (3511.17, 4004.98)	−521.57	−12.179 (−13.572, –10.749)

UI, uncertainty interval; ASIR, Age-standardized Incidence Rate. “—,” No relevant data could be derived from the calculations.

**Table 2:** Volume and ASR of global unintentional injury mortality in 1990 and 2021 and annual percentage change in age-standardized estimates between 1990 and 2021.

Characteristic	Death cases			ASR per 100,000			
	1990, *N* (95% UI)	2021, *N* (95% UI)	Percentage change (%)	1990, *N* (95% UI)	2021, *N* (95% UI)	Relative change of age-standardized rate from 1990 to 2021	Percentage change (%)
Global	1,814,129 (1,697,390, 1,932,616)	1,817,195 (1,571,722, 1,978,317)	−32.298 (−39.642, −25.730)	37.82 (35.52, 40.12)	22.79 (19.7, 24.85)	−15.03	−39.733 (−46.070, −34.096)
Sex							
Female	665,232 (615,257, 715,908)	726,275 (615,100, 804,856)	—	27.37 (25.27, 29.26)	16.85 (14.24, 18.68)	−10.52	—
Male	1,148,897 (1,063,626, 1,236,174)	1,090,920 (938,136, 1,203,051)	—	48.62 (45.29, 52.10)	29.12 (25.12, 32.12)	−19.50	—
5 SDI Region							
High-middle SDI	319,662 (303,039, 342,478)	271,032 (231,692, 303,603)	−30.850 (−42.707, −21.016)	32.86 (31.03, 35.06)	16.55 (14.33, 18.44)	−16.31	−49.623 (−57.573, −42.941)
High SDI	184,818 (174,977, 189,944)	278,624 (244,481, 297,785)	21.197 (11.134, 26.891)	18.86 (17.88, 19.39)	13.42 (12.15, 14.13)	−5.44	−28.869 (−32.482, −26.295)
Low-middle SDI	459,888 (407,764, 501,275)	479,140 (412,709, 528,206)	−37.014 (−43.280, −28.435)	48.78 (43.31, 53.28)	32.46 (28.12, 35.52)	−16.32	−33.451 (−39.236, −26.157)
Low SDI	238,289 (204,827, 274,062)	288,933 (222,308, 351,847)	−45.601 (−54.884, −34.802)	59.1 (51.93, 67.98)	41.33 (34.08, 48.42)	−17.77	−30.066 (−37.803, −19.717)
Middle SDI	609,890 (569,289, 651,511)	497,921 (419,015, 553,236)	−42.554 (−51.297, −35.359)	41.63 (39.05, 44.3)	21.06 (17.68, 23.35)	−20.57	−49.420 (−56.414, −43.792)
21 GBD Region							
East Asia	429,150 (382,148, 491,400)	305,538 (227,818, 376,437)	−41.147 (−59.234, −24.262)	41.85 (37.6, 47.44)	18.93 (14.52, 22.8)	−22.92	−54.774 (−67.610, −43.027)
Southeast Asia	137,797 (123,132, 152,121)	132,587 (113,925, 148,500)	−35.858 (−43.453, −28.000)	36.13 (31.49, 40.3)	21.73 (18.28, 24.4)	−14.40	−39.846 (−45.777, −32.763)
Oceania	1709 (1,305, 2093)	3,218 (2,612, 3,957)	−11.435 (−25.531, 6.914)	32.79 (25.17, 42.18)	28.04 (21.87, 35.91)	−4.75	−14.495 (−28.456, 2.984)
Central Asia	23,301 (22,315, 24,295)	15,695 (14,159, 17,289)	−51.270 (−55.847, −45.749)	33.95 (32.67, 35.24)	16.85 (15.26, 18.53)	−17.10	−50.358 (−54.972, −45.118)
Central Europe	41,314 (40,265, 42,241)	27,633 (25,464, 29,347)	−27.412 (−32.152, −22.908)	32.72 (31.69, 33.5)	14.68 (13.6, 15.57)	−18.04	−55.134 (−58.014, −52.355)
Eastern Europe	98,719 (97,236, 102,304)	66,807 (61,833, 71,728)	−25.868 (−32.153, −20.212)	41.4 (40.75, 42.79)	24.33 (22.6, 26.06)	−17.07	−41.244 (−45.906, −36.991)
High-income Asia Pacific	28,023 (26,642, 28,866)	50,605 (42,191, 55,585)	68.832 (45.731, 83.560)	15.92 (15.06, 16.47)	10.12 (8.95, 10.89)	−5.80	−36.396 (−41.422, −32.416)
Australasia	2,583 (2,431, 2,680)	6,318 (5,362, 6,879)	60.189 (43.668, 72.613)	12.44 (11.65, 12.94)	11.12 (9.69, 11.99)	−1.32	−10.649 (−17.344, −4.936)
Western Europe	91,922 (85,400, 95,280)	125,428 (107,072, 134,896)	19.923 (9.605, 25.188)	18.04 (16.86, 18.65)	11.91 (10.56, 12.62)	−6.13	−33.953 (−37.668, −31.681)
Southern Latin America	14,497 (14,121, 14,816)	10,544 (9,866, 11,015)	−46.772 (−49.448, −44.703)	31.35 (30.41, 32.14)	13.21 (12.42, 13.77)	−18.14	−57.868 (−59.724, −56.309)
High-income North America	44,243 (41,609,45,599)	89,649 (78,849,95,374)	54.041 (42.955,59.902)	13.89 (13.17, 14.28)	14.65 (13.23, 15.43)	0.76	5.431 (−0.501, 8.861)
Caribbean	11,700 (10,641, 12,749)	15,990 (14,449, 17,607)	1.632 (−9.945, 13.525)	36.79 (34.05, 39.57)	32.42 (29.21, 35.89)	−4.37	−11.883 (−21.252, −1.713)
Andean Latin America	16,580 (14,166, 17,921)	12,378 (10,391, 14,962)	−57.109 (−64.687, −48.379)	45.78 (39.97, 49.5)	19.87 (16.67, 24.02)	−25.91	−56.590 (−64.188, −48.247)
Central Latin America	42,711 (41,586, 43,974)	35,904 (32,245, 40,097)	−45.375 (−51.054, −39.330)	32.31 (31.51, 33.04)	14.52 (13.01, 16.26)	−17.79	−55.058 (−59.730, −49.919)
Tropical Latin America	31,513 (30,440, 32,553)	43,431 (40,168, 45,383)	−7.594 (−14.805, −2.608)	25.21 (24.22, 25.97)	18.06 (16.63, 18.94)	−7.15	−28.344 (−32.725, −25.417)
North Africa and Middle East	144,296 (129,980, 157,973)	88,599 (74,704, 102,046)	−66.570 (−70.439, −61.496)	46.04 (41.56, 50.43)	16.76 (14.25, 18.94)	−29.28	−63.601 (−67.473, −58.983)
South Asia	462,599 (395,097,514,190)	541,026 (458,956,600,874)	−30.748 (−38.432, −20.749)	57.11 (48.9, 63.68)	38.32 (32.38, 42.43)	−18.79	−32.911 (−39.519, −24.437)
Central Sub-Saharan Africa	26,195 (21,861, 30,842)	29,277 (22,321, 38,063)	−55.137 (−65.276, −41.966)	51.95 (44.21, 61.65)	34.24 (26.85, 43.01)	−17.71	−34.088 (−46.978, −18.702)
Eastern Sub-Saharan Africa	81,634 (69,168, 99,948)	89,530 (71,495, 117,388)	−50.884 (−58.836, −38.873)	59.16 (51.04, 74.59)	38.27 (32.38, 47.69)	−20.89	−35.298 (−42.575, −25.002)
Southern Sub-Saharan Africa	13,450 (11,948, 14,927)	16,792 (14,887, 18,861)	−18.506 (−28.016, −8.798)	29.61 (26.09, 33)	23.26 (20.8, 25.99)	−6.35	−21.460 (−30.873, −11.539)
Western Sub-Saharan Africa	70,193 (59,857, 80,388)	110,245 (72,167, 138,472)	−38.065 (−56.964, −20.797)	42.38 (36.75, 48.52)	34.02 (25.26, 41.28)	−8.36	−19.729 (−36.609, −3.288)

UI, uncertainty interval; ASR, Age-standardized Rate. “—,” No relevant data could be derived from the calculations.

**Table 3:** Volume and ASR of global unintentional disability-adjusted life year (DALY) rates, 1990 and 2021, and annual percentage change in age-standardized estimates between 1990 and 2021.

Characteristic	DALY cases			ASR per 100,000			
	1990, *N* (95% UI)	2021, *N* (95% UI)	Percentage change (%)	1990, *N* (95% UI)	2021, *N* (95% UI)	Relative change of age-standardized rate from 1990 to 2021	Percentage change (%)
Global	134,037,489 (122,806,901, 147,196,683)	108,119,971 (93,352,016, 125,083,408)	−45.481 (−50.995, −40.094)	2520.48 (2305.86, 2778.33)	1364.23 (1178.58, 1572.76)	−1156.25	−45.874 (−51.115, −40.707)
Sex							
Female	49,862,423 (44,890,150, 55,294,068)	42,069,581 (35,742,395, 49,409,289)	—	1882.96 (1689.12, 2104.20)	1038.67 (886.64, 1217.74)	−844.29	—
Male	84,175,065 (77,169,876, 92,685,139)	66,050,390 (56,927,668, 75,954,632)	—	3143.61 (2880.24, 3482.88)	1684.99 (1454.83, 1938.08)	−1458.62	—
5 SDI Region							
High-middle SDI	24,633,049 (22,085,018, 27,780,016)	17,218,538 (14,329,054, 20,918,512)	−42.991 (−49.314, −36.885)	2401.08 (2153.99, 2698.68)	1141.5 (959.11, 1364.65)	−1259.58	−52.459 (−57.210, −48.062)
High SDI	13,922,691 (11,499,864, 16,788,597)	15,622,493 (12,574,487, 19,315,970)	−9.792 (−13.342, −6.499)	1478.17 (1236.3, 1770.34)	1013.04 (824.18, 1248.47)	−465.13	−31.466 (−34.115, −29.008)
Low-middle SDI	32,723,647 (29,197,220, 35,971,741)	27,039,904 (23,576,398, 30,604,477)	−50.045 (−55.724, −42.946)	2740.57 (2472.02, 3014.08)	1544.54 (1348.99, 1742.96)	−1196.03	−43.642 (−48.671, −36.912)
Low SDI	17,148,184 (14,420,165, 19,736,897)	18,838,380 (14,647,336, 23,105,738)	−50.714 (−59.609, −40.041)	3043.02 (2671.07, 3491.08)	1847.12 (1504.74, 2193.5)	−1195.90	−39.300 (−47.340, −29.217)
Middle SDI	45,487,127 (42,150,378, 49,747,092)	29,293,162 (25,370,075, 33,720,060)	−54.686 (−60.032, −49.507)	2632.2 (2432.96, 2877.75)	1193.01 (1043.22, 1359.94)	−1439.19	−54.676 (−59.598, −50.048)
21 GBD Region							
East Asia	31,646,943 (28,083,448, 35,817,906)	16,659,114 (13,893,492, 19,998,213)	−56.486 (−65.706, −47.842)	2737.93 (2432.67, 3098.57)	1081.69 (921.77, 1272.53)	−1656.24	−60.492 (−67.865, −53.561)
Southeast Asia	10,037,497 (8,924,187, 11,116,198)	8,147,981 (7,203,678, 9,223,572)	−45.886 (−51.912, −39.307)	2157.34 (1943.72, 2382.65)	1212.58 (1077.43, 1365.96)	−944.76	−43.793 (−49.070, −37.993)
Oceania	124,142 (100,149, 146,833)	238,709 (200,972, 283,355)	−9.570 (−22.507, 6.248)	1890.29 (1541.38, 2280.25)	1728.98 (1463.79, 2059.07)	−161.31	−8.534 (−20.709, 6.058)
Central Asia	2,033,322 (1,866,195, 2,237,664)	1,431,041 (1,248,737, 1,679,935)	−49.085 (−53.437, −44.463)	2,835 (2577.73, 3169.69)	1482.66 (1291.2, 1738.64)	−1352.34	−47.701 (−51.585, −43.237)
Central Europe	3,432,085 (2,893,534, 4,120,750)	2,189,956 (1,724,387, 2,754,513)	−30.751 (−35.267, −26.673)	2643.08 (2249.17, 3148.26)	1422.01 (1124.28, 1790.27)	−1221.07	−46.199 (−49.959, −42.677)
Eastern Europe	7,678,174 (6,799,720, 8,819,020)	4,741,296 (4,015,159, 5,691,932)	−32.357 (−36.318, −28.234)	3274.36 (2930.33, 3727.24)	1888.27 (1614.76, 2242.4)	−1386.09	−42.332 (−45.496, −39.132)
High-income Asia Pacific	2,569,648 (2,096,955, 3,163,198)	2,564,530 (2,035,205, 3,219,266)	−6.693 (−11.684, −2.558)	1427.59 (1185.6, 1735.51)	846.7 (664.42, 1074.01)	−580.89	−40.690 (−44.889, −36.920)
Australasia	344,538 (262,987,443,664)	543,378 (408,869,710,719)	3.283 (0.392,6.061)	1620.99 (1247.18,2076.63)	1346.24 (999.33,1770.15)	−274.75	−16.949 (−19.725, −14.531)
Western Europe	6,319,838 (5,069,598, 7,806,916)	6,826,242 (5,353,788, 8,642,106)	−5.070 (−8.101, −2.599)	1397.78 (1130.96, 1721.89)	1009.36 (774.96, 1293.39)	−388.42	−27.789 (−31.113, −24.810)
Southern Latin America	1,139,946 (1,003,063, 1,319,063)	956,023 (759,698, 1,197,588)	−38.628 (−44.618, −33.029)	2343.1 (2057.24, 2715.19)	1295.41 (1040.41, 1615.46)	−1047.69	−44.714 (−49.415, −39.906)
High-income North America	3,567,723 (2,930,974, 4,340,592)	4,841,063 (3,992,509, 5,980,945)	3.153 (−0.250, 6.556)	1182.56 (982.43, 1426.9)	962.23 (804.01, 1165.43)	−220.33	−18.632 (−20.944, −16.440)
Caribbean	879,897 (774,357, 977,745)	1,057,812 (921,458, 1,209,668)	−10.599 (−20.212, −1.199)	2451.06 (2162.6, 2722.04)	2280.1 (1992.62, 2611.56)	−170.96	−6.975 (−16.164, 2.704)
Andean Latin America	1,327,493 (1,141,700, 1,457,737)	896,392 (758,849, 1,069,851)	−61.207 (−66.690, −54.931)	3214.35 (2801.48, 3546.82)	1384.17 (1169.75, 1651.93)	−1830.18	−56.938 (−62.626, −50.409)
Central Latin America	3,985,297 (3,592,463, 4,448,612)	3,068,490 (2,597,138, 3,662,128)	−49.966 (−54.039, −45.370)	2530.94 (2244.91, 2859.2)	1210.31 (1024.59, 1444.62)	−1320.63	−52.179 (−55.636, −48.341)
Tropical Latin America	2,922,807 (2,596,330, 3,323,454)	2,878,081 (2,467,506, 3,403,606)	−33.978 (−37.549, −30.516)	2051.98 (1802.15, 2358.19)	1194.64 (1027.11, 1401.51)	−857.34	−41.781 (−44.195, −39.363)
North Africa and Middle East	10,953,440 (9,923,235, 12,023,580)	6,938,887 (5,943,477, 8,129,753)	−65.510 (−69.774, −60.553)	2991.92 (2709.82, 3300.92)	1152.2 (987.02, 1351.42)	−1839.72	−61.490 (−65.728, −56.872)
South Asia	30,992,247 (27,264,148, 34,594,995)	27,458,529 (23,474,521, 31,182,639)	−47.538 (−53.662, −39.819)	2897.9 (2555.32, 3222.47)	1652.36 (1423.32, 1874.37)	−1245.54	−42.981 (−48.347, −35.966)
Central Sub-Saharan Africa	2,016,427 (1,668,296, 2,378,637)	1,976,890 (1,528,202, 2,573,115)	−60.647 (−69.289, −48.706)	2888.87 (2457.46, 3373.44)	1564.68 (1245.61, 1966.14)	−1324.19	−45.838 (−56.195, −32.968)
Eastern Sub-Saharan Africa	5,842,932 (4,903,297, 6,905,187)	5,785,495 (4,662,671, 7,534,058)	−55.656 (−63.203, −43.679)	2795.59 (2433.04, 3433.75)	1577.06 (1331.49, 1981.08)	−1218.53	−43.588 (−50.612, −32.857)
Southern Sub-Saharan Africa	975,761 (879,243, 1,080,659)	1,084,641 (962,221, 1,223,659)	−27.442 (−35.085, −19.266)	1855.81 (1672.86, 2058.76)	1366.1 (1215.89, 1540.7)	−489.71	−26.388 (−33.479, −18.200)
Western Sub-Saharan Africa	5,247,331 (4,457,202, 6,010,874)	7,835,419 (5,232,495, 9,833,594)	−41.116 (−58.917, −25.465)	2242.75 (1958.43,2 568.03)	1584.48 (1137.14, 1959.52)	−658.27	−29.351 (−45.962, −13.548)

DALYs, disability adjusted life years; UI, uncertainty interval; ASR, Age-standardized Rate.“—,” No relevant data could be derived from the calculations.

### Age- and gender-specific global trends

**Figure 2:**
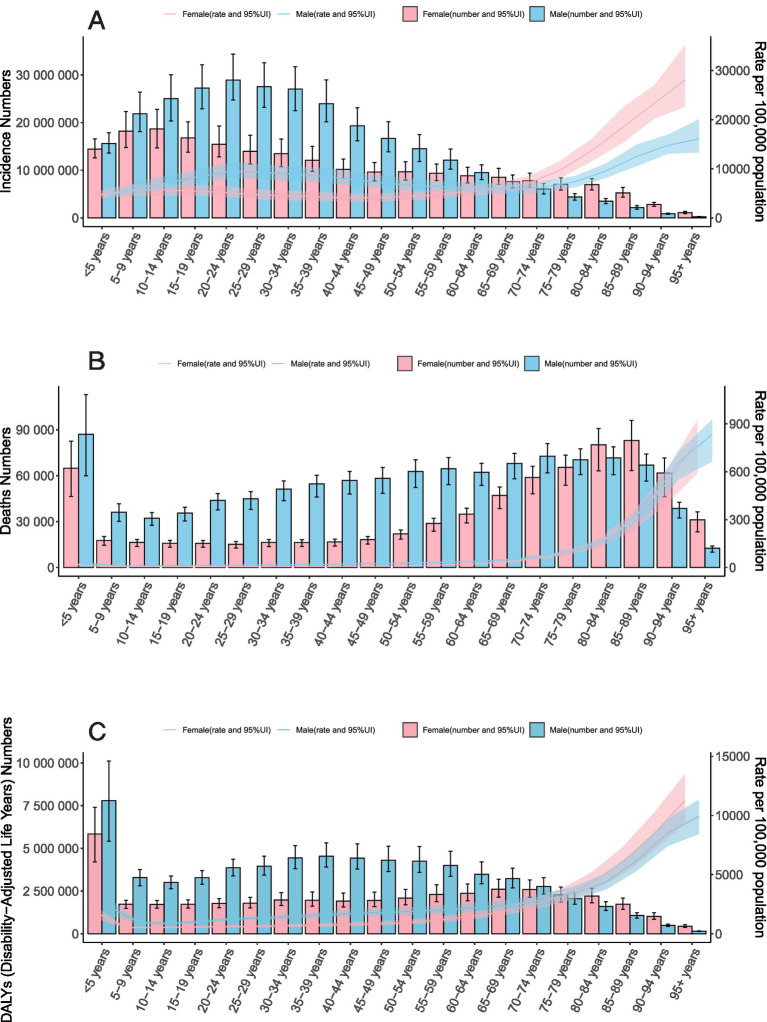
Trends in Unintentional Injuries. **A** Incidence; **B** Deaths; and **C** DALYs by Sex-Age Group, 2021.

Although the number of unintentional injury cases among older adults was lower than among children, adolescents, and middle-aged groups, the incidence rate rose steadily with age—peaking significantly among individuals aged 95 and older. From the 65–69 age group onward, female incidence rates consistently surpassed those of males (Figure 2A). Mortality and DALYs also increased with age, reaching their highest levels in the 95 + age group. However, a distinct peak in deaths and DALYs was observed among children under 5 years of age (Figures 2B,C).

### Country-specific global trends

**Figure 3:**
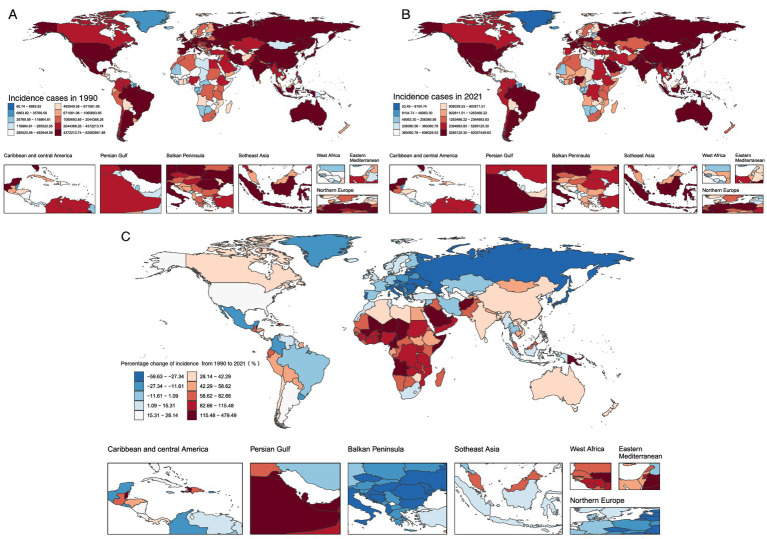
Global Trends in Unintentional Injury incidence Cases and Percentage Changes in 204 Countries and Areas **A**. Number of incidence Cases in 1990 **B**. Number of incidence Cases in 2021 **C**. Percentage Change in incidence Cases.

**Figure 4:**
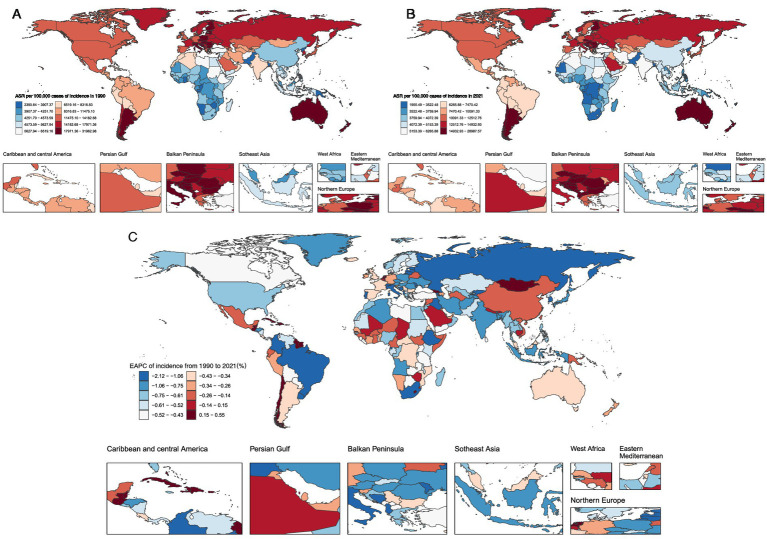
Global Trends in Age-Standardized Incidence Rates of Unintentional Injuries and Estimated Annual Percentage Changes for 204 Countries and Areas **A**. Age-Standardized Incidence Rates for 1990 **B**. Age-Standardized Incidence Rates for 2021 **C**. Estimated Annual Percentage Changes.

India (81.25 million), China (67.10 million), and the United States (37.40 million) accounted for over one-third (37%) of global unintentional injury cases (Figure 3; Supplementary Table S1). Similarly, these three countries also held the top global rankings for deaths and DALYs. Age-standardized rates (ASRs) showed that New Zealand had the highest ASIR (28.7%) (Figure 4), while Haiti exhibited the highest ASDR (0.064%) and age-standardized DALYs rate (4.39%) (Supplementary Figures S1–S4; Supplementary Tables S1–S3).

From 1990 to 2021, unintentional injury incidence rates declined in India (EAPC: −0.907, 95% CI: −0.972 to −0.841), China (EAPC: −0.159, 95% CI: −0.647 to 0.332), and the United States (EAPC: −0.191, 95% CI: −0.468 to 0.086) (Supplementary Table S1). Death rates decreased in India (EAPC: −0.880, 95% CI: −0.945 to −0.814) and China (EAPC: −1.614, 95% CI: −1.817 to −1.410) but increased in the United States (EAPC: 1.622, 95% CI: 1.489 to 1.755). DALYs rates followed a similar pattern: declining in India (EAPC: −1.876, 95% CI: −1.971 to −1.780) and China (EAPC: −2.853, 95% CI: −3.068 to −2.638) while showing a slight increase in the United States (EAPC: 0.182, 95% CI: −0.054 to 0.420) (Supplementary Tables S2, S3).

### Correlation between ASRs and SDI

**Figure 5:**
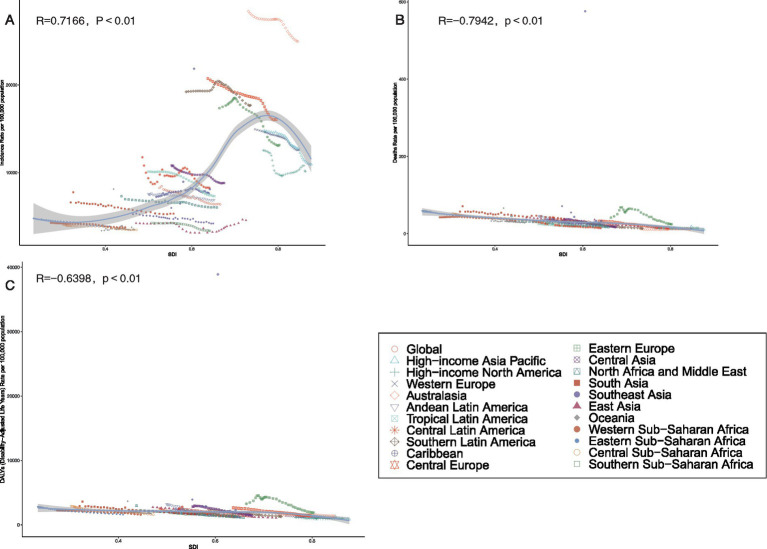
Association between age-standardized incidence, age-standardized deaths, and age-standardized disability-adjusted life year (DALY) rates of Unintentional Injuries and Regional Socio-Demographic Indicator (SDI) in Region 21, 1990-2021. **(A)** Age-standardized incidence rates. **(B)** Age-standardized deaths rates. **(C)** Age-standardized DALYs rates.

Analysis of unintentional injury burdens across 21 regions revealed nuanced associations with SDI. ASIR showed a strong positive correlation with SDI (*R* = 0.7166, *p* < 0.001), whereas ASDR and SDI exhibited a strong negative correlation (*R* = −0.7942, *p* < 0.001). Age-standardized DALYs rates displayed a similar negative correlation with SDI (*R* = −0.6398, *p* < 0.001) (Figure 5).

**Figure 6:**
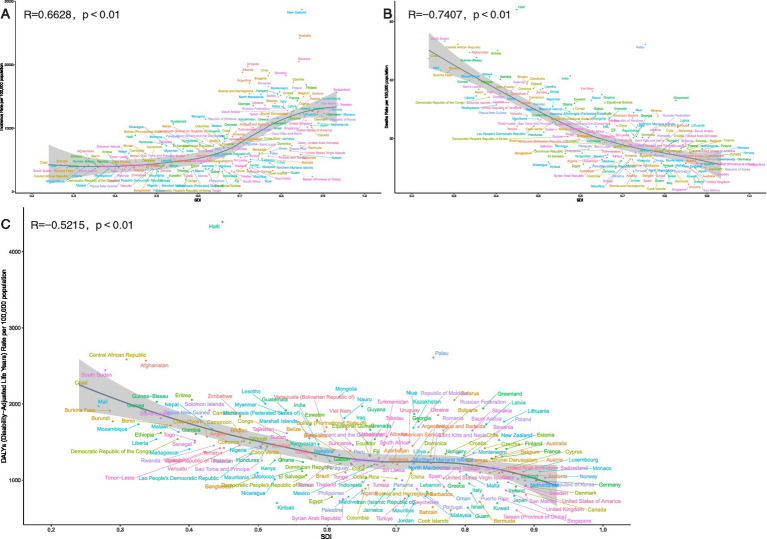
Association between age-standardized incidence, age-standardized deaths, and age-standardized disability-adjusted life year (DALY) rates for unintentional injuries and regional socio-demographic indices (SDI), 204 countries and territories, 2021. **(A)** Age-standardized incidence rates. **(B)** Age-standardized deaths rates. **(C)** Age-standardized DALYs rates.

A broader analysis of 204 countries and territories in 2021 confirmed these patterns: ASIR was strongly positively correlated with SDI (*R* = 0.6628, *p* < 0.001), while ASDR and age-standardised DALYs showed strong negative correlations (*R* = −0.7407 and *R* = −0.5215, respectively; both *p* < 0.001) (Figure 6).

### Frontier analysis

Comprehensive frontier analysis of unintentional injuries across 204 countries and territories from 1990 to 2021—based on SDI and ASRs—revealed distinct trends. For ASRs, higher SDI values corresponded with a general decline in unintentional injury-related ASRs (Fig S5). Similarly, DALYs showed an overall downward trend as SDI increased, characterized by a color density shift from lighter to darker over time. This transition illustrates that unintentional injury burdens tend to decrease with higher developmental levels (Supplementary Figure S5C).

**Figure 7:**
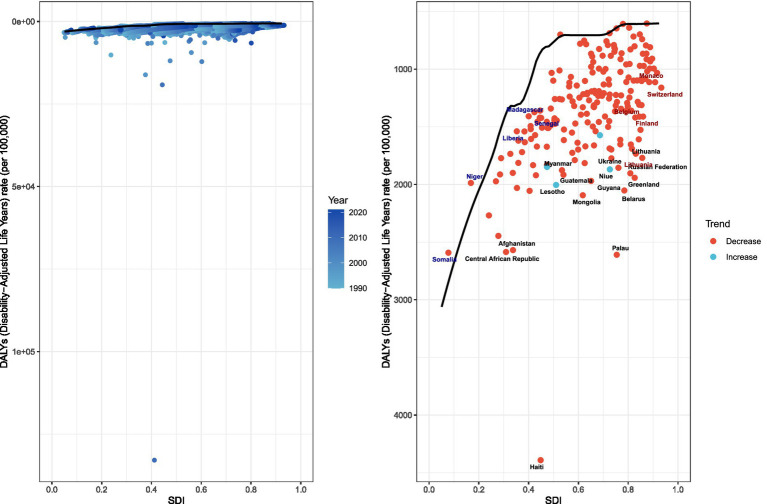
Frontier analysis, represented by the solid black line, exploring the relationship between the socio-demographic index (SDI) and the age-standardized rate (ASR) of the disability-adjusted life year (DALY) in the context of unintentional injuries.

Focusing on 2021 results, the analysis highlighted significant cross-country and regional disparities. For DALYs, countries such as Haiti, Afghanistan, and Palau exhibited substantially higher values, placing them far from the frontier. In contrast, nations including Somalia, Niger, Liberia, and Senegal were positioned closer to the frontier, reflecting rates that approached the ideal benchmarks for their respective SDI levels. These findings underscore the varying efficiency in addressing unintentional injury burdens across different developmental contexts (Figure 7).

### Trends in falls, drowning, heat, and heat-related objects

**Figure 8:**
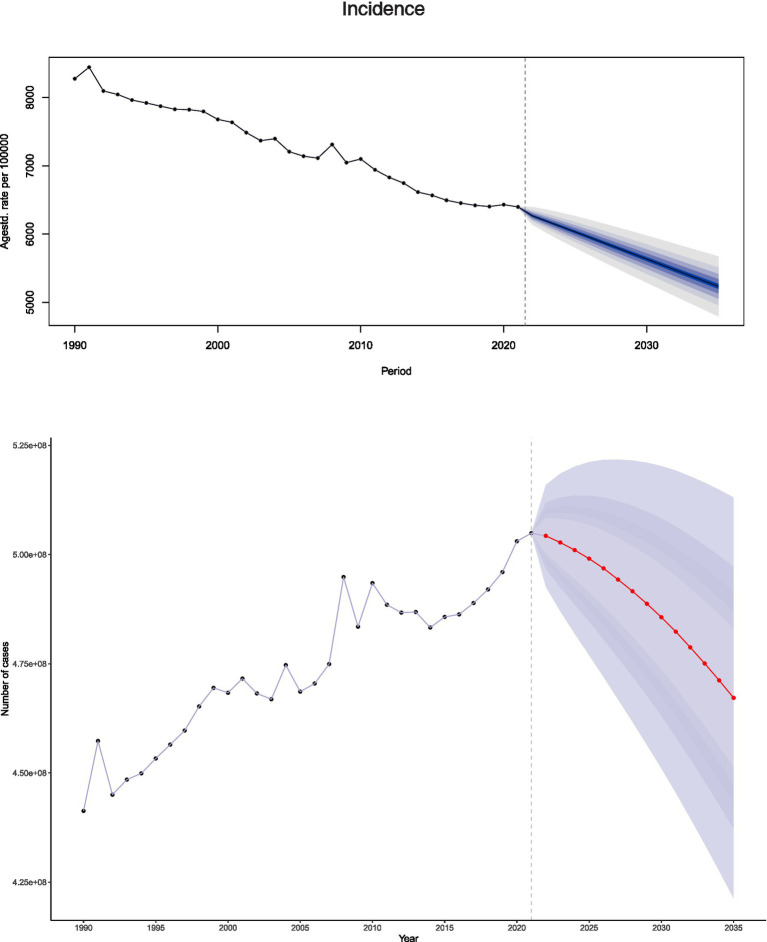
Projected figures and ASR for unintentional injury incidence from 1990 to 2035 based on BAPC modeling.

Projections for 2021–2035 indicate consistent declines in ASIR, ASDR, and age-standardized DALYs rates related to unintentional injuries. Incidence ASRs show a significant downward trajectory throughout the period, with projections indicating a marked reduction in absolute case numbers (Figure 8). Mortality and DALYs follow similar patterns: ASDRs, DALYs, and their respective ASRs consistently decrease after 2021, a trend projected to continue through 2035 alongside substantial declines in absolute deaths and DALYs (Supplementary Figures S6–S7). These patterns underscore sustained progress in mitigating the global burden of unintentional injuries over the study period.

## Discussion

From 1990 to 2021, the global ASIR of unintentional injuries dropped by 22.7%, with ASDR and age-standardized DALYs rate declining by 39.7 and 45.9%, respectively. Despite males consistently exhibiting a higher incidence rate than females, both genders saw significant reductions in ASIR. This gender disparity is plausibly linked to males’ greater engagement in high-risk activities in daily life and physiological differences such as variations in body mass index (BMI) (28). Aligning with the Global Burden of Disease analytical framework, males accumulate substantially more exposure-risk person-years to open water, elevated work surfaces, and strenuous outdoor labor. This is further compounded by higher population-attributable fractions for alcohol consumption and non-use of personal protective equipment, collectively contributing to a greater incidence of unintentional injuries (29–33). In contrast, females experience lower exposure through predominantly domestic activities and visits to lifeguard-supervised public pools, resulting in a lower overall occurrence of unintentional injuries. However, due to osteoporosis and differential vasomotor control, females demonstrate poorer post-injury trajectories—specifically, higher non-fatal disability weights (YLDs) for hip fractures following falls and for syncope during heatwaves (34–36).

To concurrently reduce premature mortality and disability burdens across both sexes, targeted interventions should include mandated alcohol restrictions, enforced engineering controls, and compulsory work-rest cycles during heat exposure for males. For older females, priorities should include osteoporosis screening, age-friendly home modifications, and early heatwave warning systems. These observed declines underscore the effectiveness of preventive interventions and the positive impact of public health education in enhancing awareness of safety practices and health-related behaviors (37–39). Projections for 2021 to 2035 indicate continued decreases in the absolute numbers of cases, deaths, and DALYs, along with reductions in age-standardized rates—trends attributable to growing global attention and strengthened measures aimed at mitigating the burden of unintentional injuries.

From 1990 to 2021, the global incidence of unintentional injuries increased by 13.5%, while the age-standardized incidence rate (ASIR) dropped by 22.72%—revealing a nuanced epidemiological pattern. This divergence indicates that although the absolute number of cases rose—likely driven by lifestyle shifts, inadequate supervision, physiological variations, population aging, and comorbid health conditions— (40–42)the decline in age-standardized rates reflects adjustments in preventive policies and advancements in public safety infrastructure (43–45). For instance, in its 2012 updated advisory statement, the U.S. Preventive Services Task Force (USPSTF) recommended exercise or physical therapy alongside vitamin D supplementation to prevent falls among community-dwelling adults aged 65 years or older at elevated risk. This intervention has been shown to reduce fall incidence significantly (46). These findings underscore the value of targeted management strategies for unintentional injuries. Despite the rise in total cases, the success of focused preventive measures offers an optimistic outlook, emphasizing the need to continue exploring effective approaches to further reduce related health metrics.

At the national level, India, China, and the United States reported the highest number of cases in 2021, drawing attention to critical issues: the risk factors for unintentional injuries (including falls, drowning, heat exposure, and heat-related incidents), the urgency of expediting improvements to safety infrastructure, and the implementation of preventive and intervention strategies (45, 47). The sustained decline in incidence rates in these three countries—evident in their negative Estimated Annual Percentage Changes (EAPCs)—suggests that their prevention strategies, policy shifts, and enhanced public health education have yielded results. In China, for example, a comprehensive national injury surveillance system has been established, which employs the Haddon Matrix and the “5E” framework to evaluate intervention effectiveness, identify optimal prevention strategies, and create an enabling policy environment. This system has effectively reduced the burden of unintentional injuries (48). In China, the age-standardized mortality rate (ASDR) for unintentional injuries fell from 46.18 per 100,000 in 1990 to 19.06 per 100,000 in 2021, while the age-standardized DALYs rate decreased from 2775.574 per 100,000 to 1089.777 per 100,000 over the same period. However, to address overlapping challenges such as rapid urbanization, motorization, and population aging, China must scale local evidence to national standards through cross-sector data sharing and rigorous cost-effectiveness analyses (48). Similarly, the U.S. Centers for Disease Control and Prevention launched the STEADI initiative (Stopping older adults Accidents, Deaths & Injuries) to support primary care providers in integrating fall risk screening, assessing modifiable risk factors, and delivering evidence-based interventions (49). In the United States, the ASIR for unintentional injuries declined from 12,628.483 per 100,000 in 1990 to 10,158.231 per 100,000 in 2021. Nonetheless, rising trends in certain countries highlight the urgent need to address underlying risk factors, public health challenges, socioeconomic conditions, and gaps in intervention efforts. These variations emphasize the need to customize health strategies to each country’s specific context, accounting for unique challenges and leveraging individual strengths.

The association between SDI and health outcomes related to unintentional injuries revealed distinct correlations. Higher SDI levels showed a strong negative correlation with ASDR and age-standardised DALYs, highlighting the advantages of advanced socioeconomic development in lowering these metrics. This connection may stem from the implementation of comprehensive preventive strategies (43, 44), safer living environments (45), mental health interventions (37),improved health literacy (50), and robust healthcare systems—factors more commonly found in regions with medium-to-high SDI levels (38, 46, 51). On the other hand, the age-standardized incidence rate (ASIR) displayed a strong positive correlation with SDI, potentially influenced by factors such as increased life expectancy, more refined injury reporting mechanisms, and enhanced social and economic conditions (52, 53). While these data underscore the role of SDI in reducing the burden of unintentional injuries, they also emphasize the need to incorporate SDI improvements into health policy frameworks to achieve sustainable reductions. It should be noted that our SDI correlation analyses identify disparities in injury burden but do not establish causal relationships. Future studies integrating subnational socioeconomic data, behavioral surveys (e.g., WHO STEPwise), and policy implementation metrics are required to clarify the drivers of these disparities.

Frontier analysis, based on SDI and age-standardized rate (ASR) trends from 1990 to 2021, offers a comprehensive view of global unintentional injury outcomes. The analysis shows that as SDI improves, DALY-based outcomes tend to decrease consistently. However, a closer look at 2021 data revealed subtle differences between countries. For example, nations like Haiti, Afghanistan, and Palau had disproportionately high DALYs, placing them far from optimal outcomes. Haiti, for instance, had the highest ASDR and age-standardized DALYs rates. In 2021, Haiti’s ASDR for unintentional injuries was 64.104 per 100,000, and its age-standardized DALYs rate was 4,391.002 per 100,000. Although Haiti is classified as a lower SDI country, it bears an excessively high disease burden (in terms of DALYs). This excess burden is rooted in a long history of political instability, repeated coups, authoritarian rule, and successive foreign interventions, which have gradually eroded state capacity and left the economy chronically fragile (54, 55). The 2010 earthquake was a critical exacerbating factor. The disaster decapitated national leadership, paralyzed policy formulation and implementation, and caused widespread damage to already weak infrastructure and emergency response capabilities, thereby significantly increasing the population’s exposure to unintentional injuries (56, 57). Of the 49 health facilities in the affected area, 30 were partially or completely destroyed, including the country’s only tertiary referral center—dealing an unbearable blow to an already fragile health system (58). Coupled with the structural inability to convert emergency surge resources into sustained trauma care, these factors collectively explain why Haiti’s disease burden is vastly inconsistent with its SDI level (55). In contrast, countries such as Niger and Somalia, despite facing significant sociopolitical and economic challenges, had DALYs closer to the frontier, more in line with the ideal baseline for their SDI levels. These disparities highlight the diversity of healthcare outcomes and emphasize that factors beyond SDI—such as environmental conditions, genetic predispositions, and unique national healthcare policies—play a role in shaping unintentional injury trends.

### Study limitations

This study has several limitations. While it offers a thorough analysis of the global, regional, and national burdens of unintentional injuries, its scope is bounded by the data accessible in the GBD database—including potential gaps in recorded information and inherent limitations in modeling approaches—which may introduce inaccuracies. The GBD study, while groundbreaking in quantifying global health metrics, harbors inherent limitations in its dataset construction and modeling approaches that have yet to be fully acknowledged. A key oversight lies in the variability of data quality across countries and regions. Its technical framework for standardizing data has not adequately addressed the intrinsic disparities among nations—specifically in disease surveillance capacities, the completeness of case reporting, and the consistency of statistical standards. This systemic failure to account for cross-national differences in data quality not only risks underestimating biases in model outputs but also weakens, to some degree, the interpretability and reliability of research findings when conducting cross-regional comparisons. Addressing these issues will require methodological innovations in future research, making such advancements imperative for refining the robustness of global health analyses.

Moreover, although the study examined trends in unintentional injuries from 1990 to 2021 and projected these trends through 2035 using the Bayesian Age-Period-Cohort (BAPC) model, external variables such as environmental shifts, policy revisions, and advancements in medical technology could compromise the precision of these projections. Even though the GBD 2035 projections draw on extensive datasets, three critical sources of uncertainty warrant emphasis. First, climate change is already reshaping the fundamental social and environmental determinants of health on a global scale; extreme weather events, for instance, can undermine health infrastructure. Watts et al. (2020) demonstrate that exposure to high temperatures and heatwaves triggers a cascade of adverse health effects, leading to excess mortality among older adults and individuals with chronic conditions (59, 60). Second, armed conflict is inherently unpredictable. Protracted violence can generate long-term, large-scale internal displacement, and relevant data often excludes cross-border refugee flows into neighboring countries. GBD models typically assume geopolitical stability, and any deviation from this assumption can rapidly render projections invalid (61, 62). Third, uneven progress in medical technology and health system expansion may significantly alter future disease burdens. The GBD study is committed to iteratively incorporating new evidence and transparently communicating these limitations to enhance the credibility of its 2035 health landscape projections.

Notably, while advanced diagnostic methods (e.g., residual plots) were not integrated into the analysis, our methodologies adhere to the standards of the GBD consortium, ensuring comparability across studies. The reliance on aggregated GBD data precluded the use of causal mediation analyses or multivariable regression to examine specific determinants such as infrastructure quality or occupational factors. Future research should incorporate individual-level datasets to address these questions. Thus, while the findings provide valuable insights for managing unintentional injuries globally, they should be interpreted with caution, acknowledging the potential biases and uncertainties inherent in this body of research.

## Conclusion

This study clarifies the critical associations between trends in unintentional injuries, sociodemographic indices, and population factors, providing valuable insights for policy adjustments, regional management strategies, and health education initiatives. The findings indicate a significant decline in the ASIR from 1990 to 2021, along with lower ASDR and age-standardized DALYs in high SDI regions. This reflects a reduced burden in more developed areas, though disparities among countries persist. The overall downward trend in ASIR underscores the effectiveness of existing interventions and policies. That said, sustained research and the development of innovative health policies remain crucial to further mitigate the impact of unintentional injuries on a global scale.

## Data Availability

The original contributions presented in the study are included in the article/Supplementary material, further inquiries can be directed to the corresponding authors.
