# Supplementary figures and images for: Global, regional, national burden and trends of unintentional injuries from 1990 to 2021 and projections to 2035: a systematic analysis of the Global Burden of Disease study 2021
Source: Front Public Health. 2025 Sep 3;13:1653491. doi: 10.3389/fpubh.2025.1653491 (PMC12442766; doi:10.3389/fpubh.2025.1653491)

A

Deaths cases in 1990

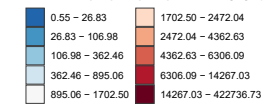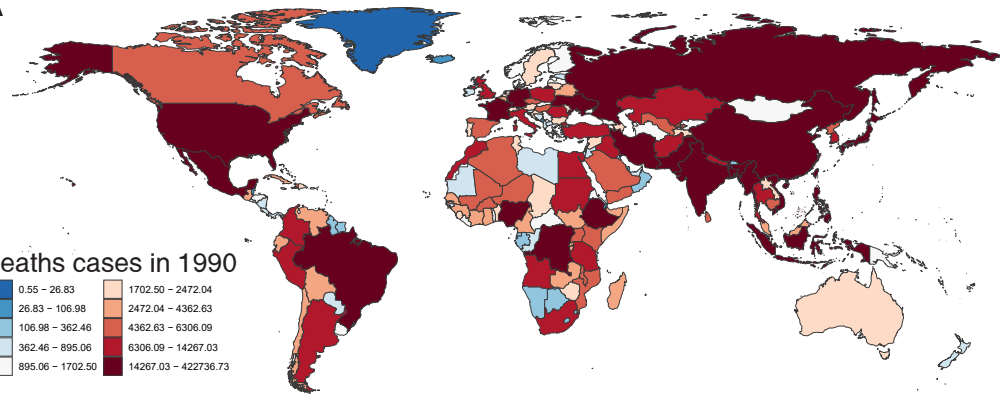

B

Deaths cases in 2021

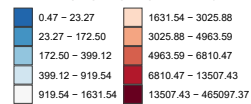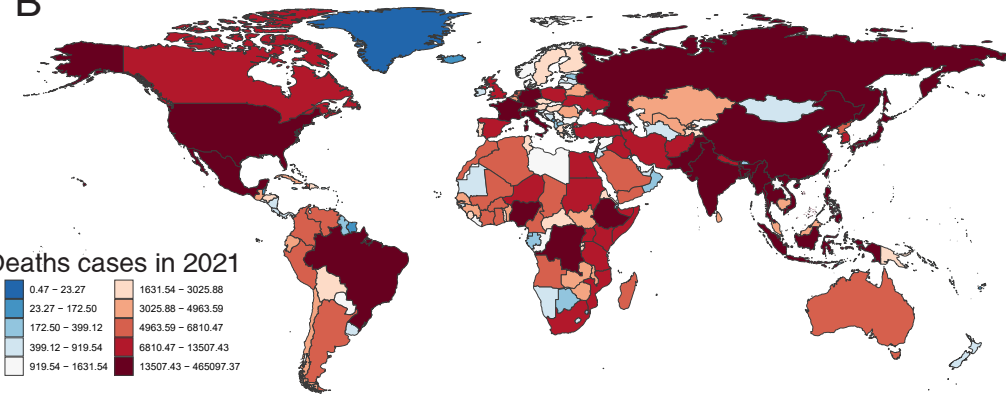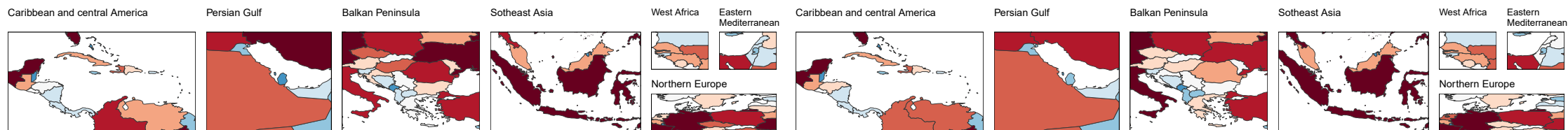

C

Percentage change of deaths from 1990 to 2021 ( % )

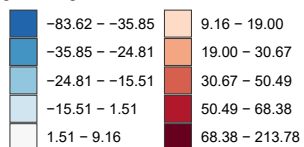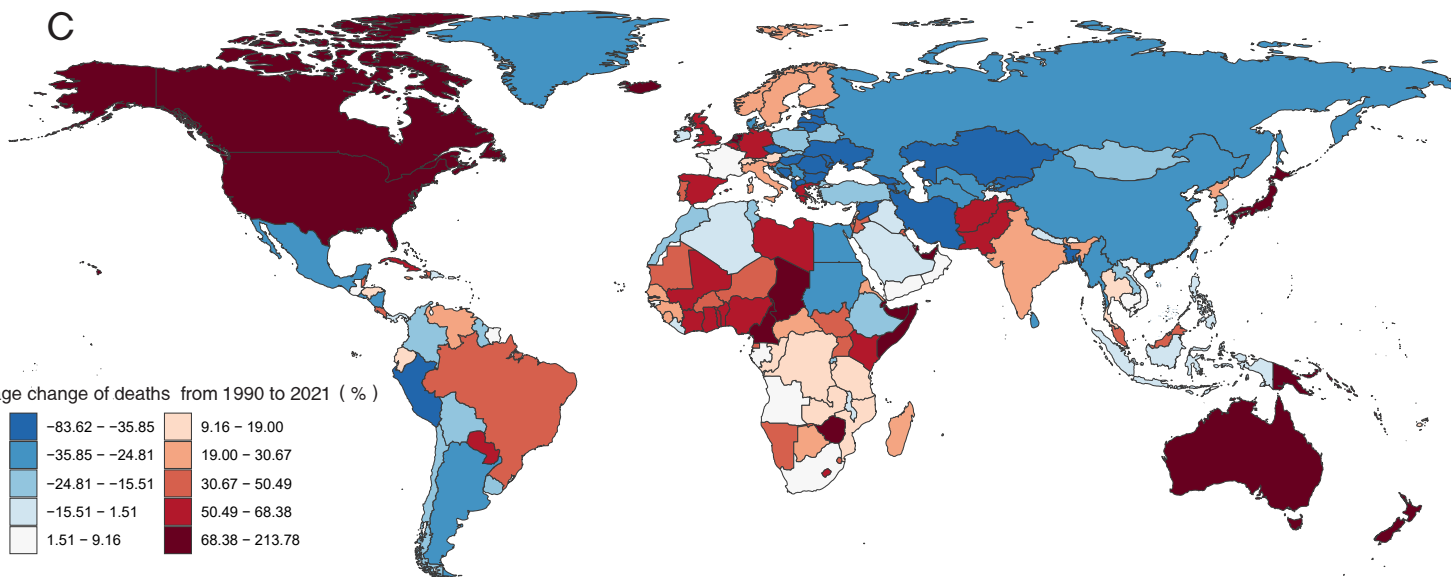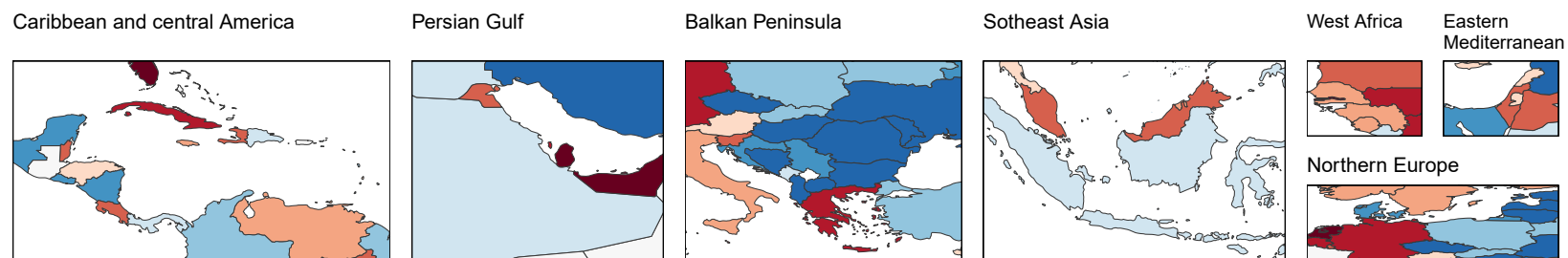

Supplement: Supplementary file 1 [file Data_Sheet_1.pdf]

A

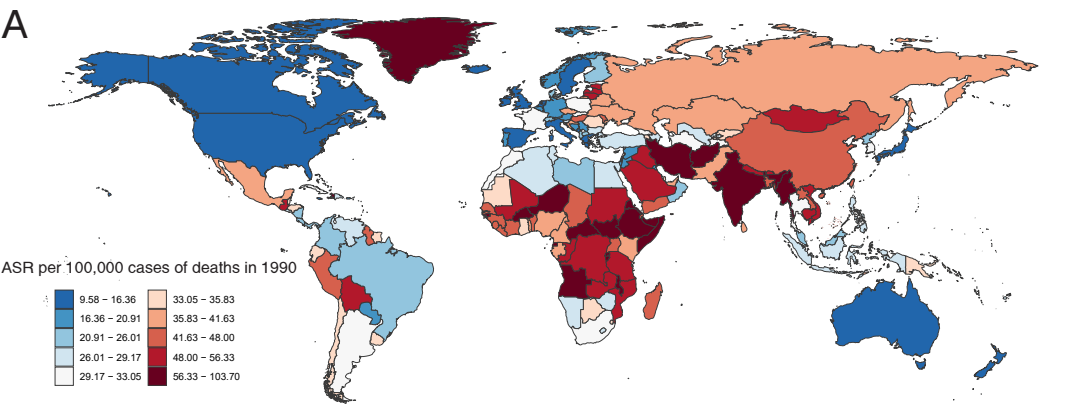

B

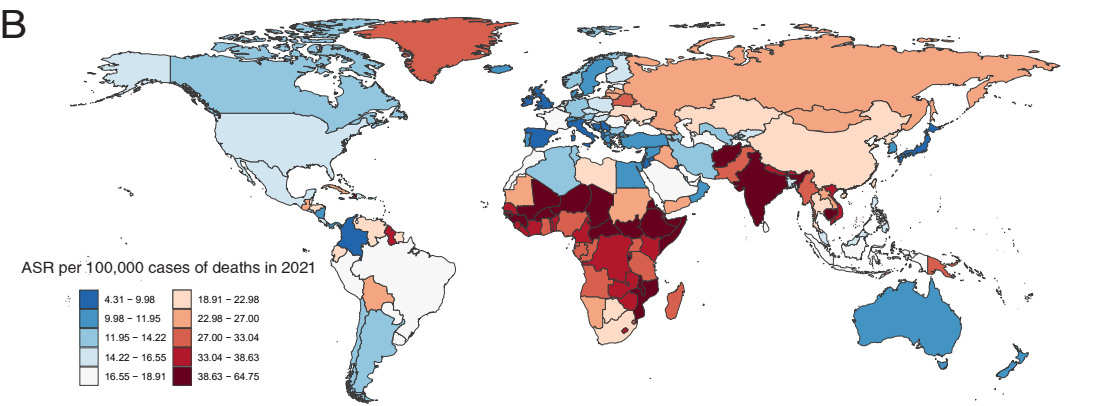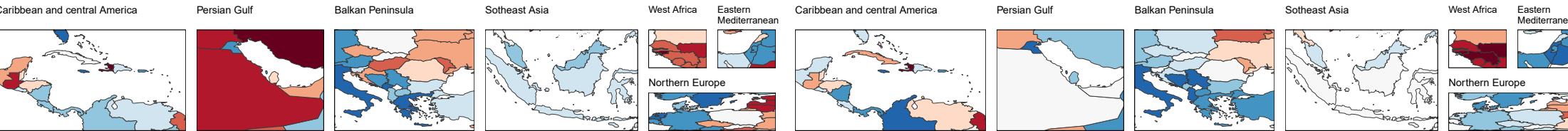

C

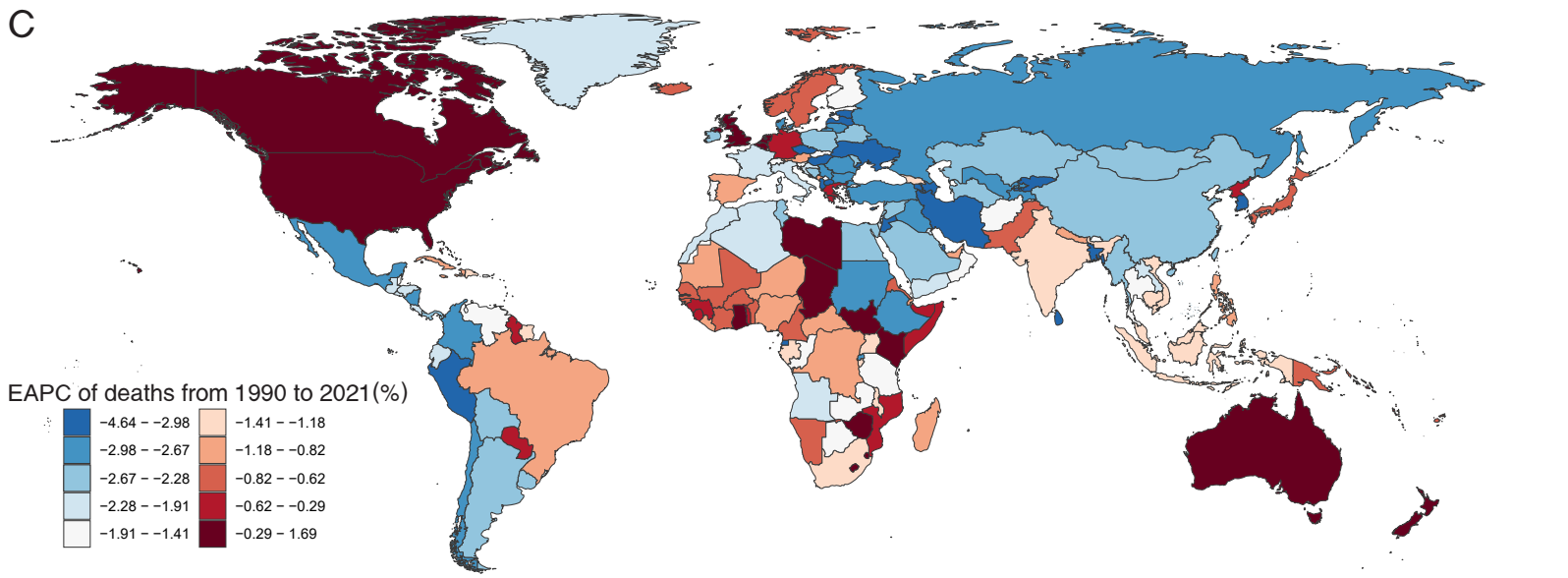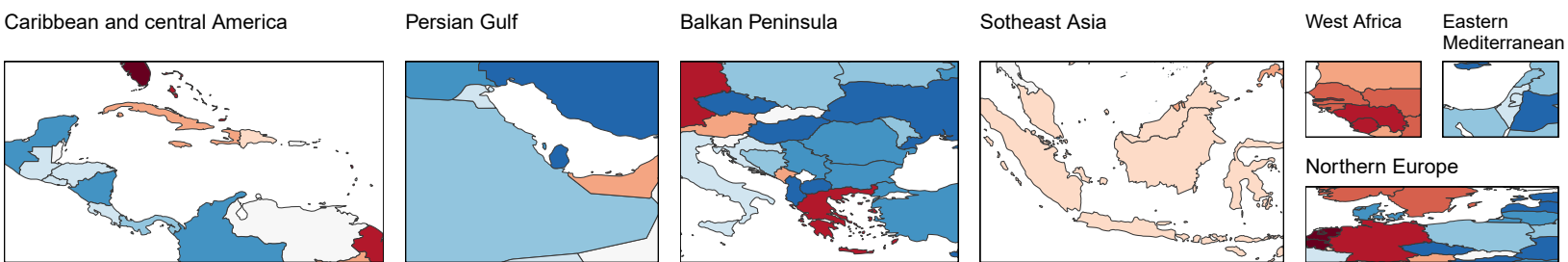

Supplement: Supplementary file 2 [file Data_Sheet_2.pdf]

A

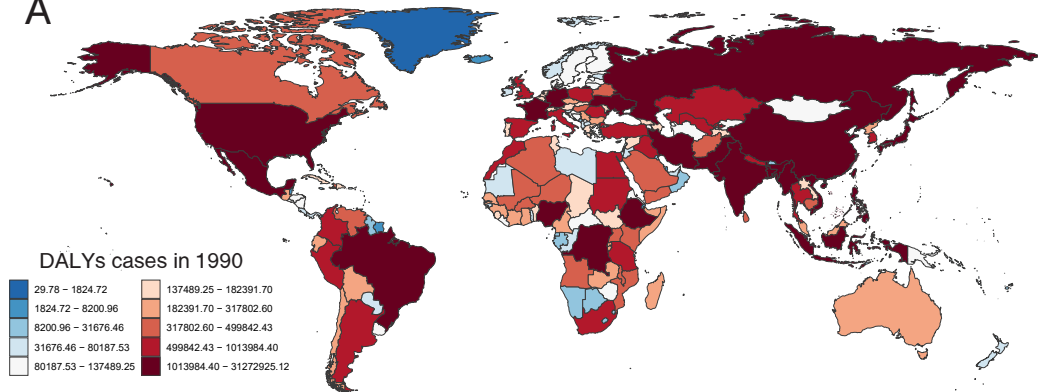

B

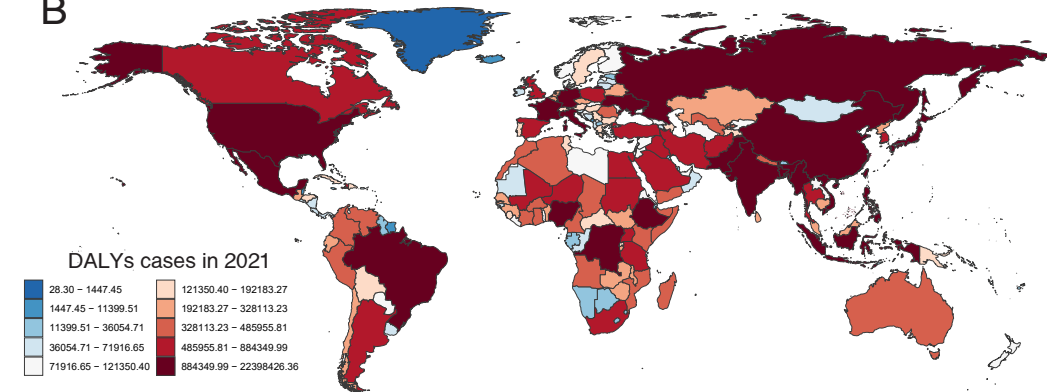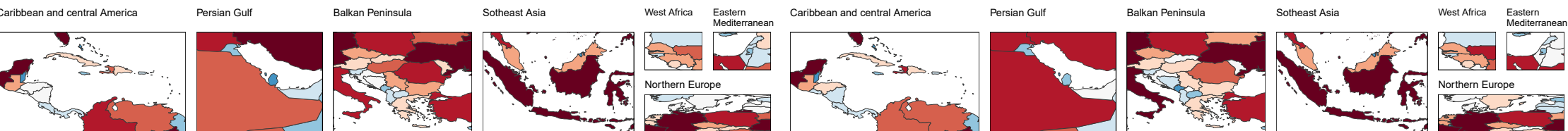

C

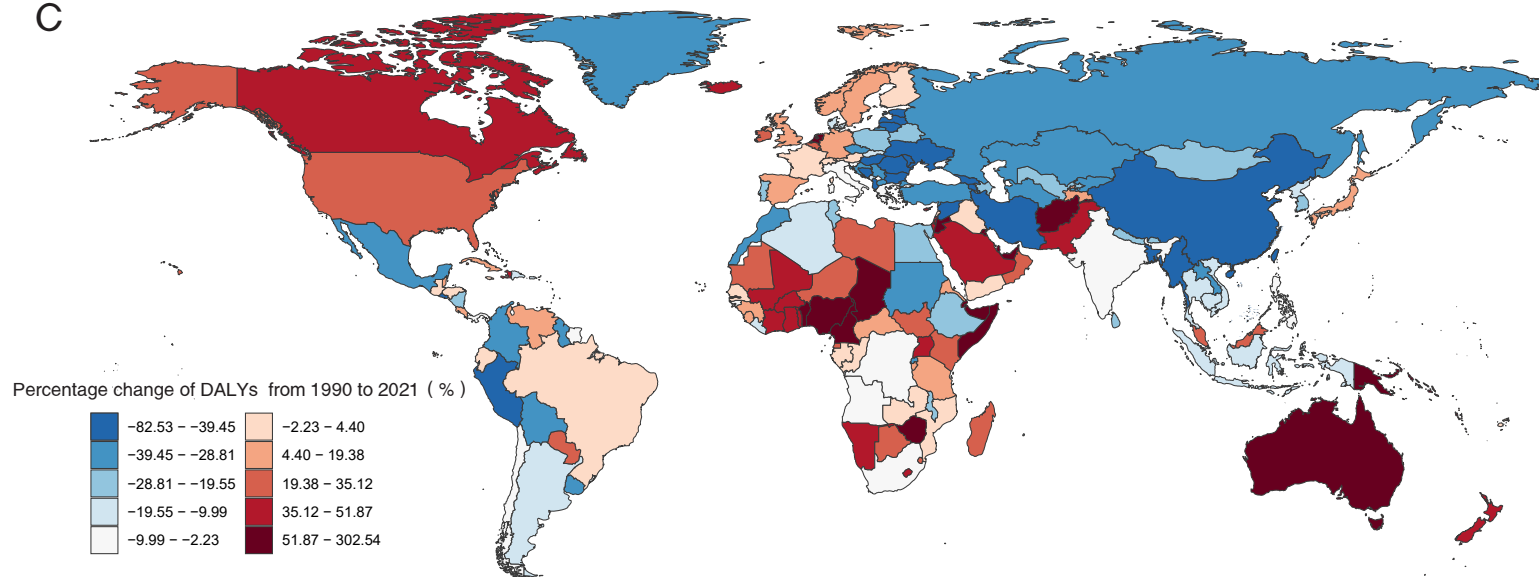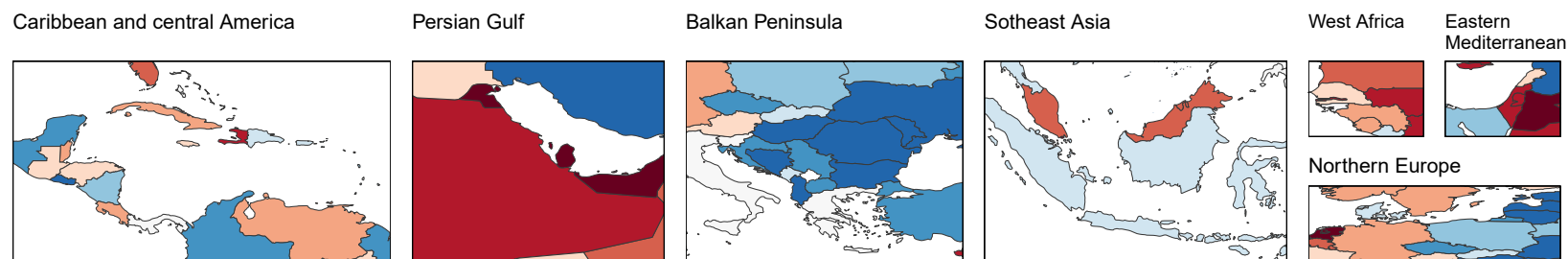

Supplement: Supplementary file 3 [file Data_Sheet_3.pdf]

A

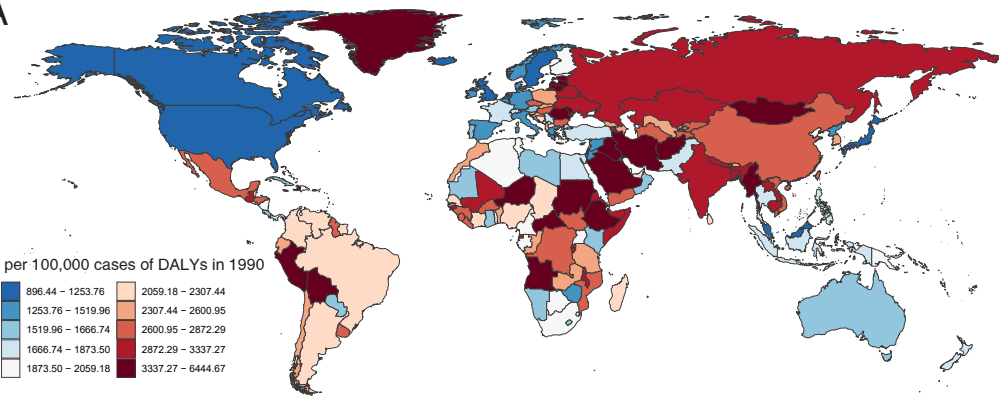

B

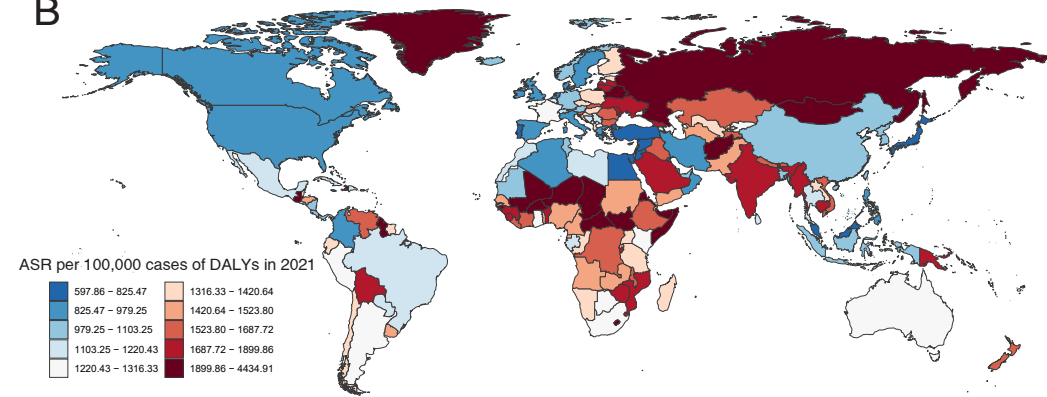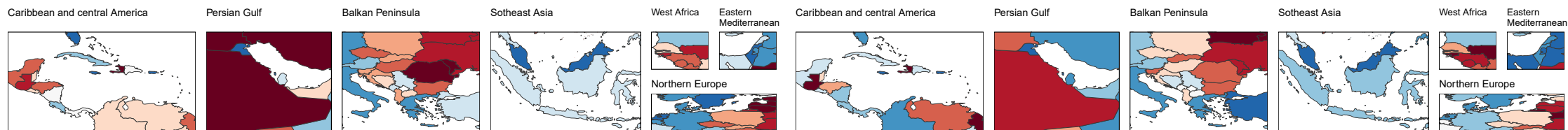

C

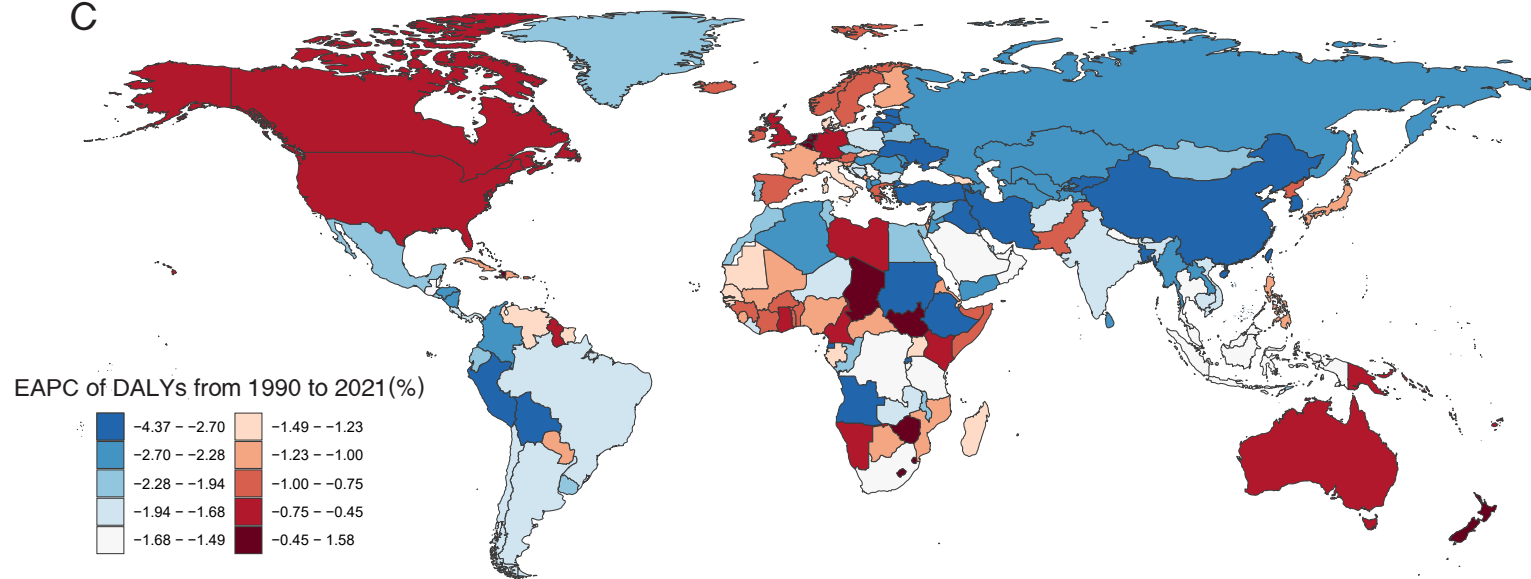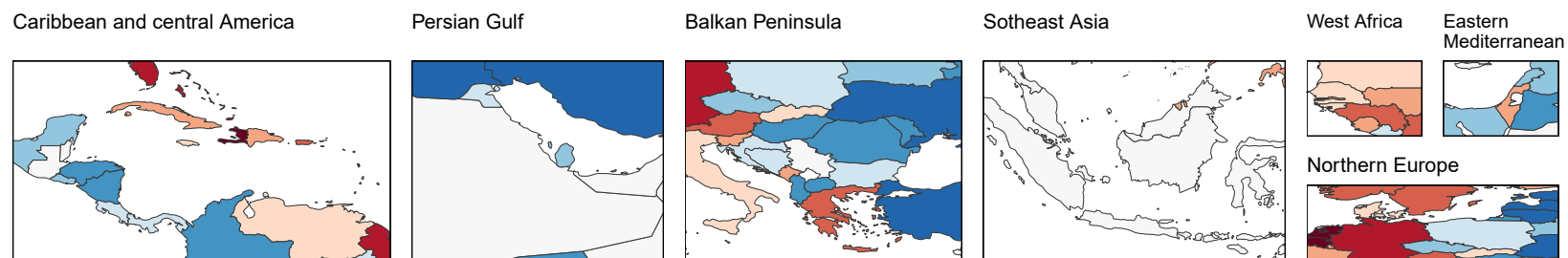

Supplement: Supplementary file 4 [file Data_Sheet_4.pdf]

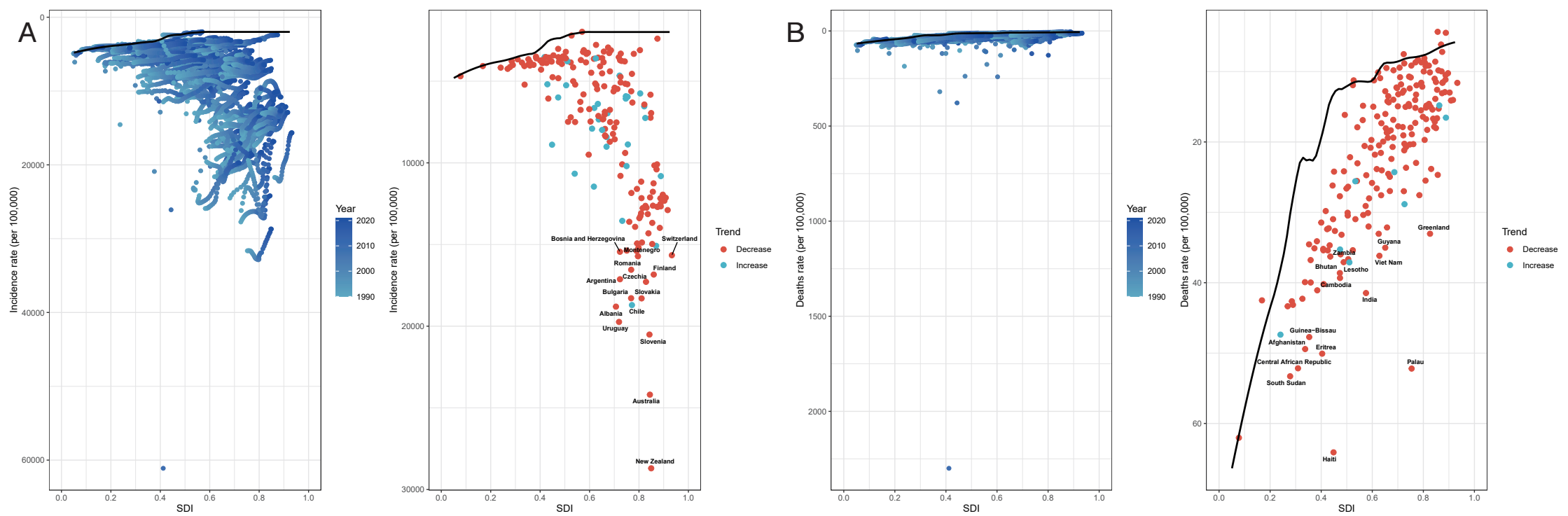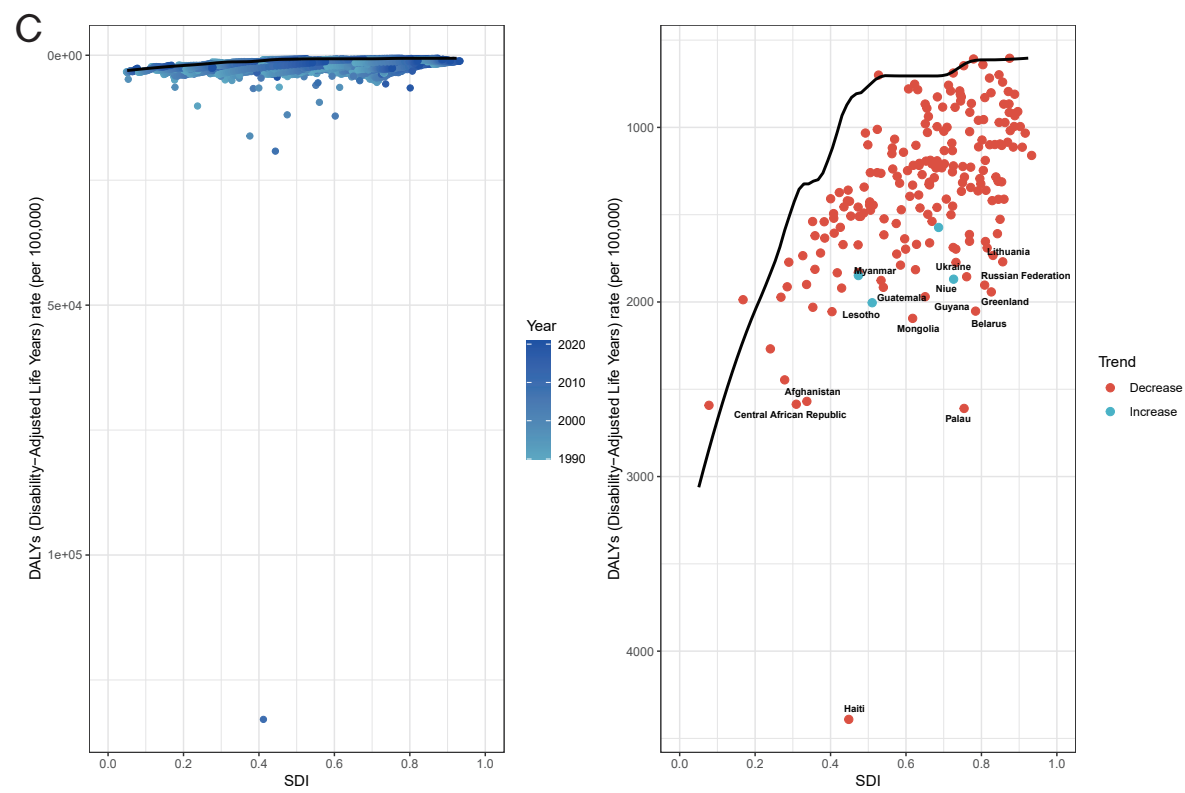

Supplement: Supplementary file 5 [file Data_Sheet_5.pdf]

# Deaths

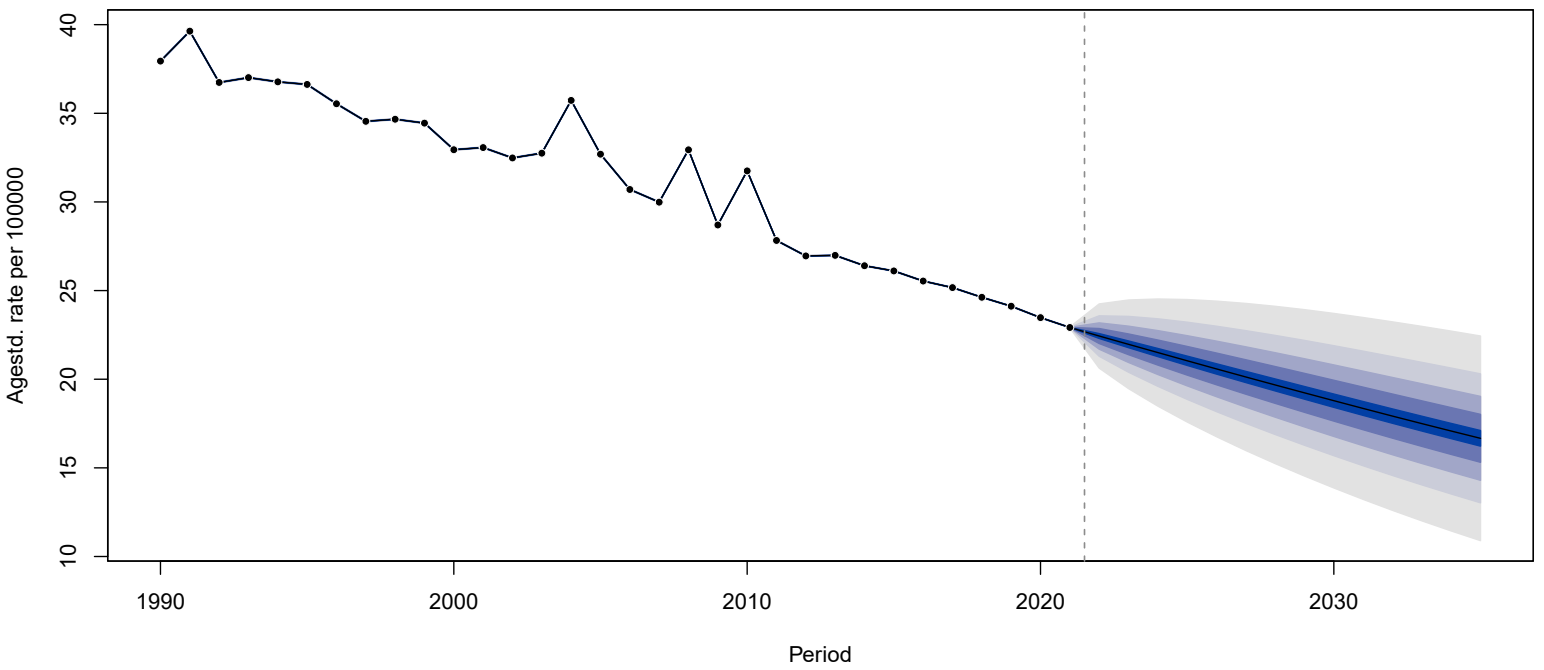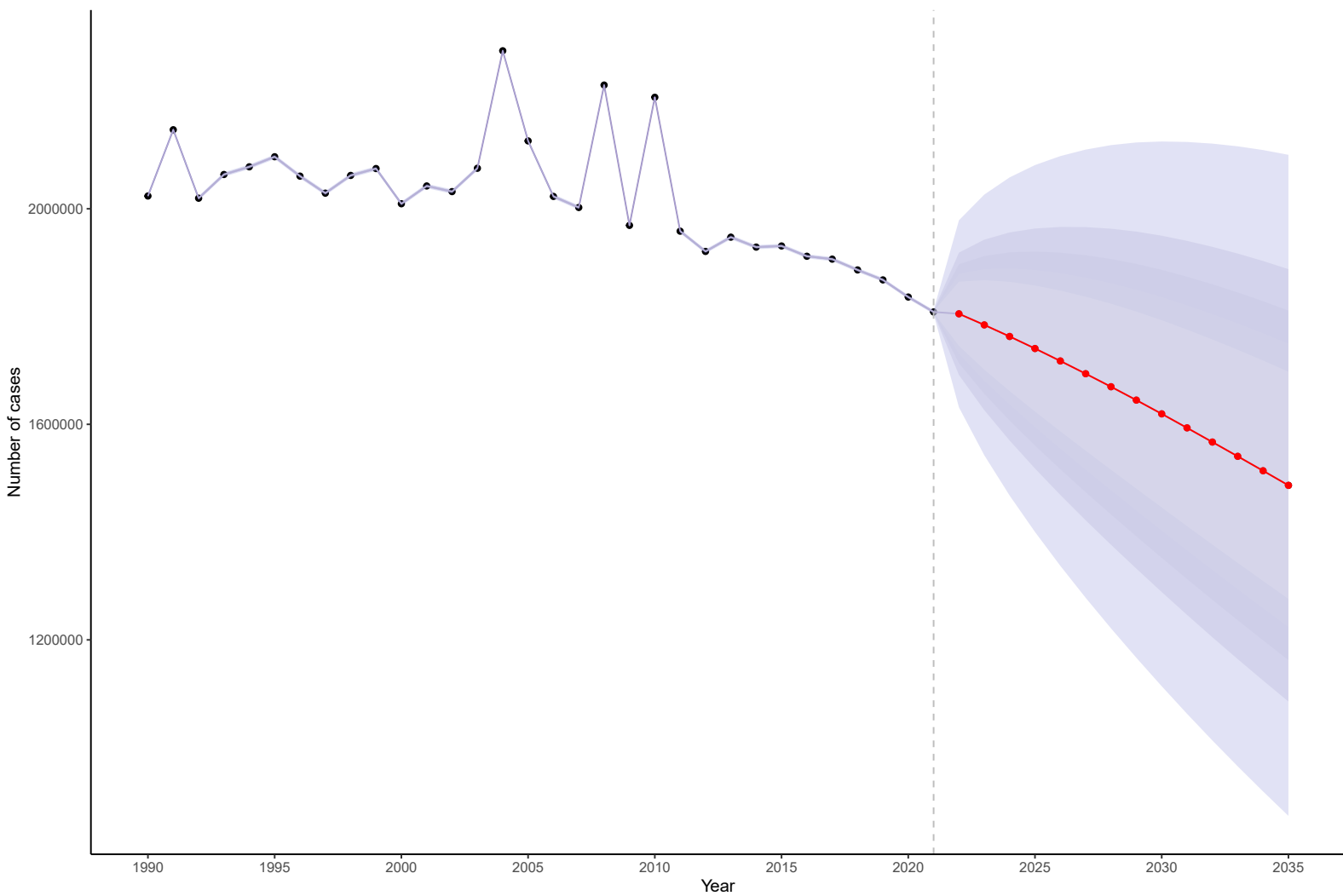

Supplement: Supplementary file 6 [file Data_Sheet_6.pdf]

# DALYs

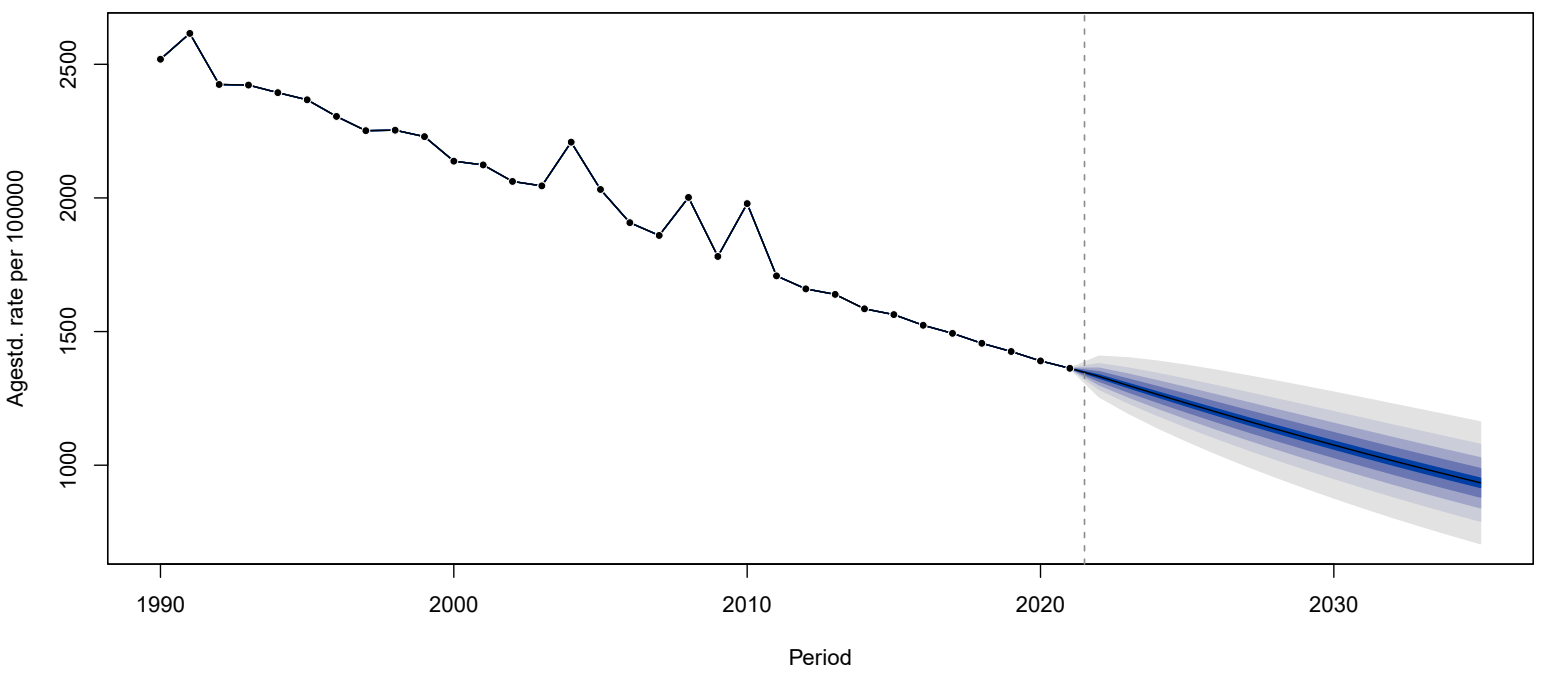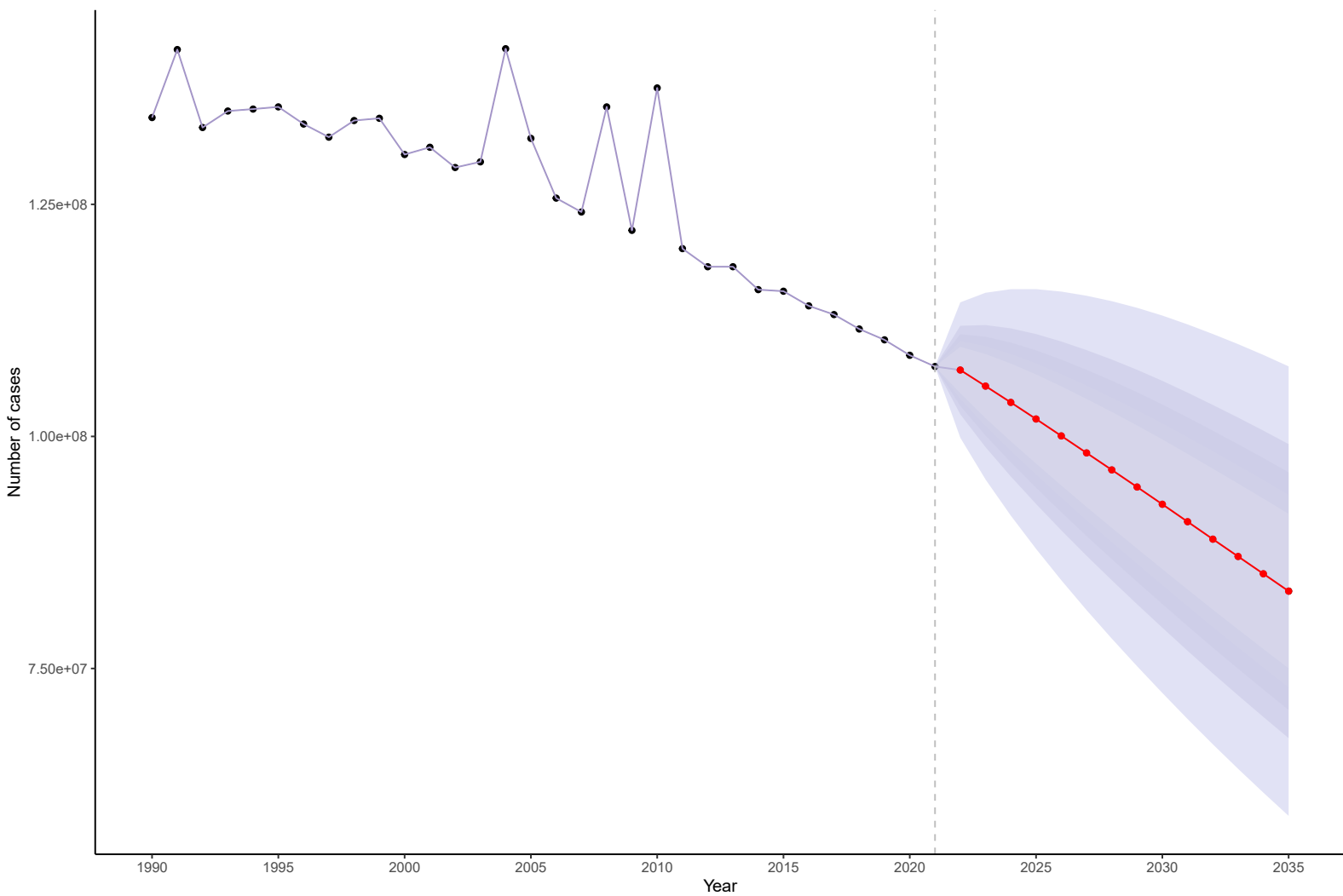

Supplement: Supplementary file 7 [file Data_Sheet_7.pdf]
