# Supplementary material for: Global, regional, national burden and trends of unintentional injuries from 1990 to 2021 and projections to 2035: a systematic analysis of the Global Burden of Disease study 2021
Source: Front Public Health. 2025 Sep 3;13:1653491. doi: 10.3389/fpubh.2025.1653491 (PMC12442766; doi:10.3389/fpubh.2025.1653491)
Supplement: Supplementary file 8 [file Table_1.docx]

**Table S1:** Unintentional injury incidence and ASRs per 100,000 cases, by 204 countries and territories, 1990 and 2021, and EAPC per 100,000 ASRs, 1990-2021

| Characteristic | Number in 1990 | Age-standardized Rate in 1990 (95% CI) | Number in 2021 | Age-standardized Rate in 2021 (95% CI) | EAPC (Incidence rates) |
| --- | --- | --- | --- | --- | --- |
| India | 61448210(56696206,66456644) | 7336.962(6812.902,7872.494) | 81245000(75866552,87017087) | 5808.183(5427.179,6198.103) | -0.907(-0.972,-0.841) |
| China | 50347974(45178440,57441600) | 4270.162(3849.629,4803.919) | 67103655(60750989,75772764) | 4656.912(4211.575,5183.399) | -0.159(-0.647,0.332) |
| United States of America | 32094305(29163465,35113261) | 12628.483(11436.198,13852.546) | 37396861(34784109,40157371) | 10158.231(9390.784,10967.636) | -0.191(-0.468,0.086) |
| Russian Federation | 26169839(23667028,28633018) | 17971.357(16182.493,19698.835) | 17766730(16428075,19110965) | 13086.787(12079.143,14094.884) | -1.417(-1.750,-1.084) |
| Brazil | 15965670(14461880,17845824) | 10163.958(9220.095,11357.325) | 15990179(14761644,17400127) | 7328.441(6780.347,7987.805) | -1.486(-1.677,-1.294) |
| Japan | 16417316(14972414,17949366) | 13588.123(12306.904,14906.196) | 11767413(10822735,12770520) | 10398.333(9359.41,11466.47) | -1.183(-1.278,-1.088) |
| Mexico | 12213576(10918461,13634864) | 13082.136(11788.586,14417.08) | 10660413(9637770,11725757) | 8371.283(7535.642,9224.688) | -0.426(-0.983,0.134) |
| Indonesia | 9797015(8867344,10721519) | 5134.033(4705.368,5571.291) | 10443682(9637903,11259219) | 3835.378(3543.193,4131.39) | -1.085(-1.310,-0.859) |
| Germany | 10098587(9401660,10883795) | 13348.059(12237.017,14586.443) | 10030374(9380350,10756443) | 12319.613(11200.124,13614.969) | -0.257(-0.313,-0.201) |
| France | 9548387(8913043,10189884) | 16549.417(15352.365,17833.884) | 9652464(9076216,10314897) | 14312.843(13165.595,15562.802) | -0.315(-0.377,-0.252) |
| Nigeria | 4262126(3895135,4638298) | 4377.143(4042.361,4710.214) | 8676596(7972453,9464365) | 3597.562(3345.113,3853.852) | -0.803(-0.920,-0.687) |
| Pakistan | 4319830(3926560,4735554) | 3721.399(3415.323,4060.112) | 7752048(7128783,8411114) | 3201.725(2978.543,3437.3) | -0.620(-0.916,-0.322) |
| Argentina | 6536212(5893931,7140757) | 19586.912(17676.182,21318.064) | 7558099(6843527,8217118) | 17125.652(15421.407,18675.069) | -0.528(-0.802,-0.253) |
| United Kingdom | 6906755(6315966,7524130) | 12921.924(11748.725,14265.364) | 7211386(6621416,7839301) | 11253.563(10217.081,12384.057) | -0.340(-0.450,-0.230) |
| Italy | 10088958(9172621,11130877) | 18379.295(16624.96,20361.793) | 7086380(6601436,7588074) | 13264.136(12102.334,14493.669) | -1.583(-1.717,-1.449) |
| Republic of Korea | 8335546(7699440,9000470) | 17740.086(16493.43,19085.275) | 6056390(5641816,6471617) | 12171.731(11120.741,13172.086) | -1.841(-1.967,-1.715) |
| Bangladesh | 4372214(3951595,4809420) | 3690.714(3371.809,4020.248) | 5892544(5356990,6491374) | 3483.446(3177.095,3826.441) | -1.102(-2.081,-0.114) |
| Australia | 4513695(4044392,4996830) | 27738.47(24751.092,30949.94) | 5803495(5253798,6331684) | 24192.908(21359.218,26967.281) | -0.474(-0.524,-0.424) |
| Saudi Arabia | 2143690(1989322,2302307) | 12999.125(12169.125,13882.478) | 5385354(5037823,5767945) | 12771.684(12015.243,13546.154) | 0.492(0.191,0.793) |
| Poland | 7220925(6465821,7964870) | 19233.573(17174.809,21198.343) | 5320507(4870333,5788521) | 14875.273(13364.534,16469.125) | -1.023(-1.120,-0.925) |
| Ukraine | 8060925(7324032,8872520) | 16164.487(14675.974,17767.997) | 5265125(4820045,5684266) | 13608.809(12467.798,14741.077) | -1.095(-1.257,-0.933) |
| Viet Nam | 3819631(3497717,4170492) | 5598.833(5175.134,6056.586) | 5157908(4825634,5473932) | 5246.621(4911.917,5573.781) | -0.260(-0.354,-0.165) |
| Egypt | 3361797(3094157,3633993) | 5574.098(5150.495,6010.732) | 4929714(4552541,5348829) | 4481.858(4157.434,4839.434) | -0.852(-0.933,-0.771) |
| Iran (Islamic Republic of) | 5400159(4997169,5861462) | 8876.573(8285.837,9529.308) | 4791399(4413380,5167882) | 5619.905(5167.857,6068.987) | -1.103(-1.314,-0.891) |
| Spain | 5089283(4693861,5481453) | 13560.838(12465.213,14665.437) | 4732494(4395813,5086056) | 11843.731(10743.044,13051.479) | -0.758(-0.833,-0.683) |
| Ethiopia | 3128313(2878583,3381332) | 5881.989(5455.247,6274.697) | 4506157(4150092,4899162) | 4041.806(3764.506,4317.442) | -1.377(-1.466,-1.288) |
| Turkey | 3903864(3566086,4246087) | 6375.812(5856.73,6894.236) | 4489444(4163422,4851737) | 5475.775(5057.771,5926.001) | -0.800(-0.925,-0.675) |
| Canada | 3279816(3037846,3520300) | 11704.863(10827.957,12574.971) | 4254974(4003629,4498445) | 10092.859(9357.368,10783.238) | -0.107(-0.147,-0.068) |
| Philippines | 3333911(3064340,3633332) | 5028.747(4671.493,5430.93) | 4135890(3807668,4474219) | 3583.075(3305.955,3863.912) | -0.778(-1.111,-0.445) |
| Iraq | 2235178(2049330,2437294) | 11475.096(10623.986,12461.645) | 3628770(3360507,3920116) | 8320.01(7747.517,8947.229) | -1.157(-1.346,-0.967) |
| Colombia | 3870839(3512747,4224376) | 10991.999(10036.013,11895.78) | 3321758(3009934,3623532) | 7321.603(6570.76,8078.613) | -1.686(-1.768,-1.604) |
| Chile | 2319618(2089190,2537926) | 16852.259(15243.703,18447.783) | 3300553(3038646,3561971) | 18706.526(17076.959,20313.444) | 0.163(-0.023,0.349) |
| Democratic Republic of the Congo | 1634903(1499998,1787104) | 3942.64(3668.181,4220.288) | 3233870(2980127,3503864) | 3495.971(3274.507,3726.615) | -0.556(-0.661,-0.451) |
| Thailand | 3029589(2784265,3278745) | 5126.67(4743.821,5525.12) | 2862474(2696904,3034247) | 4397.35(4096.62,4697.591) | -0.877(-1.044,-0.710) |
| Myanmar | 2630427(2444743,2820163) | 6200.964(5800.622,6593.461) | 2824794(2660435,3007282) | 4998.987(4710.589,5310.038) | -0.831(-1.909,0.258) |
| Romania | 4884381(4488474,5280590) | 21616.561(19814.685,23428.092) | 2777064(2553633,3013172) | 16543.933(14960.673,18157.514) | -1.329(-1.396,-1.262) |
| Uzbekistan | 2220443(2013961,2426011) | 9510.792(8681.688,10355.004) | 2759089(2520576,2994411) | 7921.505(7236.456,8614.229) | -0.972(-1.117,-0.826) |
| Peru | 1764625(1642309,1897532) | 7595.179(7133.995,8124.449) | 2524213(2341612,2714208) | 6873.498(6378.662,7394.367) | -0.520(-0.553,-0.488) |
| Sudan | 1318319(1213446,1423573) | 6174.217(5737.502,6647.577) | 2430449(2252307,2635038) | 5205.623(4861.489,5575.714) | -0.558(-0.624,-0.493) |
| Venezuela (Bolivarian Republic of) | 2306412(2060845,2576306) | 10916.475(9881.324,12068.373) | 2379710(2145821,2614864) | 9498.515(8526.158,10505.262) | -1.084(-1.442,-0.724) |
| United Republic of Tanzania | 1256922(1148710,1374037) | 4354.237(4043.58,4676.409) | 2364664(2172303,2575328) | 3801.462(3538.559,4056.852) | -0.563(-0.618,-0.507) |
| Algeria | 1814235(1668508,1956081) | 6665.07(6158.71,7121.795) | 2348839(2185509,2528597) | 5311.014(4952.05,5705.988) | -1.005(-1.092,-0.919) |
| Morocco | 2044368(1883134,2211131) | 7620.128(7033.697,8186.185) | 2339663(2170984,2505881) | 6265.879(5812.755,6697.514) | -0.905(-1.012,-0.797) |
| Kazakhstan | 2179938(2012398,2358479) | 12743.94(11792.959,13753.22) | 2036051(1886183,2190991) | 10798.09(10000.152,11653.462) | -0.682(-0.786,-0.578) |
| Nepal | 1245972(1153600,1350806) | 6240.107(5805.958,6693.176) | 1931341(1788898,2074427) | 6064.285(5647.503,6472.701) | -0.038(-0.481,0.407) |
| Kenya | 1093639(991494,1206489) | 4434.039(4080.684,4784.157) | 1924237(1770163,2097715) | 3853.009(3588.06,4138.868) | -0.835(-1.014,-0.657) |
| Netherlands | 1521740(1395678,1665975) | 10584.698(9572.236,11666.24) | 1921002(1799648,2051891) | 10810.692(9860.768,11835.355) | 0.495(0.095,0.898) |
| South Africa | 1775680(1624779,1929626) | 4481.24(4139.776,4829.445) | 1893712(1754627,2049820) | 3276.505(3041.323,3535.439) | -1.287(-1.468,-1.105) |
| Guatemala | 1020242(920272,1132569) | 10154.762(9360.563,11021.227) | 1757197(1629075,1911929) | 10664.336(9905.917,11533.88) | -0.271(-0.318,-0.223) |
| Belgium | 1482535(1378086,1601528) | 15575.006(14274.244,17051.093) | 1725583(1619161,1832407) | 14968.089(13753.62,16396.905) | 0.280(-0.121,0.682) |
| Afghanistan | 646537(594592,696647) | 6310.283(5839.252,6773.308) | 1714289(1587509,1849869) | 5210.654(4864.083,5556.856) | -0.736(-0.925,-0.547) |
| Yemen | 801046(732656,869456) | 5627.944(5191.742,6060.722) | 1682886(1554485,1819624) | 4753.831(4421.515,5077.694) | -0.490(-0.572,-0.408) |
| Czechia | 2455471(2223840,2681069) | 24498.867(21971.25,26947.73) | 1676359(1537226,1817719) | 17284.829(15507.733,19168.804) | -1.149(-1.335,-0.964) |
| Uganda | 777396(707602,852225) | 4022.951(3706.276,4349.097) | 1647022(1512499,1805209) | 3521.282(3290.151,3781.22) | -0.572(-0.677,-0.467) |
| New Zealand | 1062954(945537,1172961) | 31646.518(28023.888,35239.199) | 1368762(1259070,1469673) | 28700.566(26107.727,31160.343) | -0.494(-0.565,-0.423) |
| Hungary | 2062948(1892854,2231812) | 20074.496(18053.136,21936.903) | 1364046(1250130,1473188) | 15398.785(13674.051,17144.437) | -1.244(-1.338,-1.151) |
| Switzerland | 1467362(1371257,1564838) | 21698.779(20041.265,23356.812) | 1353634(1265927,1442327) | 15655.78(14295.228,17168.546) | -1.321(-1.423,-1.219) |
| Ghana | 624727(573686,681766) | 3907.37(3639.377,4195.994) | 1292088(1196777,1395722) | 3704.774(3467.002,3951.014) | -0.306(-0.386,-0.227) |
| Ecuador | 797216(736439,861384) | 7466.38(6954.038,7964.39) | 1291841(1201549,1383688) | 7046.486(6555.623,7537.602) | -0.426(-0.528,-0.323) |
| Sweden | 1219305(1112448,1332381) | 14963.822(13512.137,16544.049) | 1275489(1172321,1390363) | 12512.763(11341.28,13832.974) | -0.407(-0.523,-0.291) |
| Mozambique | 628634(575830,684180) | 4251.704(3949.446,4553.841) | 1263466(1161480,1367021) | 3863.156(3623.26,4122.038) | -0.514(-0.625,-0.402) |
| Cameroon | 442953(406310,482688) | 3947.45(3677.301,4224.697) | 1234619(1144685,1335379) | 3743.406(3508.169,3965.883) | -0.256(-0.337,-0.176) |
| Malaysia | 749971(678231,822177) | 4180.314(3826.541,4513.855) | 1214296(1125417,1315330) | 3788.199(3513.324,4093.514) | -0.405(-0.440,-0.370) |
| Haiti | 555561(507398,609107) | 8109.738(7476.465,8888.808) | 1203287(1108678,1304356) | 8887.41(8229.515,9612.622) | 0.140(-1.298,1.599) |
| Belarus | 1481588(1375634,1595538) | 14738.779(13584.596,15958.214) | 1201763(1130480,1276024) | 13917.549(12918.381,15038.861) | -0.336(-0.633,-0.038) |
| Sri Lanka | 1027372(954246,1106110) | 5791.23(5404.176,6189.437) | 1200512(1134780,1277462) | 5400.398(5088.048,5768.217) | -0.681(-1.512,0.157) |
| Serbia | 1567975(1398841,1716720) | 17049.71(15152.207,18744.72) | 1195986(1080966,1299185) | 14932.933(13179.89,16564.137) | -0.578(-0.635,-0.521) |
| Austria | 1355098(1267104,1447531) | 17929.219(16595.875,19341.025) | 1168387(1092028,1246868) | 13673.552(12512.71,14927.655) | -0.881(-0.932,-0.830) |
| Angola | 493995(456554,534126) | 4470.72(4178.225,4772.68) | 1154161(1061646,1249518) | 3418.822(3196.513,3645.668) | -1.054(-1.147,-0.960) |
| Côte d'Ivoire | 556038(508576,603535) | 4202.611(3923.447,4474.892) | 1126531(1044923,1214393) | 3884.107(3644.965,4131.702) | -0.399(-0.448,-0.350) |
| Madagascar | 612582(564833,667096) | 4668.662(4363.409,4999.022) | 1118932(1033360,1218191) | 3731.411(3481.392,3996.378) | -0.842(-0.914,-0.769) |
| Niger | 404921(369533,441322) | 4419.938(4098.028,4718.042) | 1113174(1025610,1205451) | 4072.386(3810.973,4337.681) | -0.383(-0.449,-0.318) |
| Mali | 434248(398831,469998) | 4489.602(4188.894,4792.725) | 1112445(1032490,1199285) | 4242.508(3983.368,4498.495) | -0.182(-0.238,-0.126) |
| Somalia | 417100(382621,453345) | 4925.152(4569.929,5268.631) | 1060811(978471,1143504) | 4694.949(4371.186,5005.609) | -0.301(-0.505,-0.098) |
| Bulgaria | 1670436(1539405,1799944) | 20996.182(19281.437,22823.4) | 1050564(963084,1138276) | 18287.311(16498.694,20097.3) | -0.693(-0.760,-0.627) |
| Israel | 674598(610193,743067) | 13283.892(12054.039,14563.789) | 1046833(949881,1155983) | 11150.647(10088.552,12360.062) | -0.616(-0.714,-0.517) |
| Greece | 1373453(1272513,1484118) | 14037.734(12890.822,15272.361) | 1000574(930060,1073984) | 11596.192(10550.874,12777.894) | -0.996(-1.100,-0.891) |
| Cuba | 843040(774633,914849) | 7729.676(7119.298,8362.441) | 991224(922592,1055392) | 9011.582(8223.139,9784.514) | 0.419(0.283,0.556) |
| Burkina Faso | 432483(396161,469548) | 4059.997(3769.862,4330.767) | 924631(855994,996604) | 3781.442(3550.168,4018.649) | -0.346(-0.375,-0.317) |
| Slovakia | 1139484(1041792,1245283) | 21688.027(19781.395,23778.268) | 923447(853200,992182) | 18297.46(16562.227,20062.026) | -0.774(-0.809,-0.740) |
| Portugal | 1370652(1275549,1474068) | 14182.679(13131.99,15277.482) | 902812(846373,967189) | 9392.143(8589.693,10267.502) | -1.664(-1.757,-1.570) |
| Finland | 936244(869569,1007527) | 19364.676(17766.825,21210.901) | 892844(835553,954599) | 16838.637(15350.305,18707.355) | -0.619(-1.129,-0.106) |
| Azerbaijan | 725181(652386,795505) | 9156.909(8286.774,9967.839) | 844274(769831,919939) | 8229.025(7487.961,8989.939) | -0.660(-0.721,-0.599) |
| Cambodia | 523072(482201,566995) | 4921.744(4579.871,5280.143) | 811459(758952,866123) | 4815.258(4531.07,5120.43) | -0.039(-0.233,0.156) |
| Tajikistan | 568656(515994,623881) | 9506.975(8692.691,10361.425) | 809959(732593,887340) | 7498.796(6816.083,8188.488) | -1.152(-1.286,-1.019) |
| Chad | 285521(261255,310495) | 4225.024(3925.275,4513.598) | 809707(750560,871310) | 4170.521(3923.182,4412.942) | -0.107(-0.175,-0.039) |
| Bolivia (Plurinational State of) | 514324(475389,555346) | 7602.333(7068.536,8117.847) | 804557(748731,862956) | 6709.144(6256.915,7169.123) | -0.602(-0.640,-0.564) |
| Honduras | 524320(467920,585870) | 9211.074(8381.208,10081.729) | 785986(706266,868306) | 7485.833(6749.075,8196.399) | -1.512(-2.099,-0.923) |
| Tunisia | 707471(645235,770304) | 8002.304(7356.631,8665.478) | 784406(724629,844952) | 6753.13(6219.279,7277.065) | -0.795(-0.861,-0.730) |
| Malawi | 464804(428867,506299) | 4223.562(3932.64,4526.714) | 742647(682579,808366) | 3597.276(3351.778,3839.214) | -0.713(-0.790,-0.636) |
| Zambia | 364799(334259,399140) | 4179.733(3892.869,4482.324) | 739991(685336,802530) | 3672.827(3452.2,3923.961) | -0.675(-0.781,-0.569) |
| Dominican Republic | 454831(411442,498850) | 5817.784(5324.043,6302.473) | 739493(674294,800344) | 6638.049(6064.621,7189.038) | 0.229(0.004,0.455) |
| Syrian Arab Republic | 784076(715719,863957) | 5549.94(5116.469,6024.646) | 727981(663213,802252) | 5140.01(4723.669,5634.175) | -0.548(-0.793,-0.304) |
| United Arab Emirates | 161433(148993,175212) | 7730.136(7174.261,8322.159) | 719488(663644,791513) | 6950.435(6502.042,7466.56) | -0.071(-0.283,0.142) |
| Norway | 671061(612583,732700) | 15858.112(14406.043,17379.175) | 705379(650848,766241) | 12891.61(11724.552,14196.936) | -0.596(-0.709,-0.482) |
| Denmark | 722088(672755,775956) | 14063.593(12817.712,15404.633) | 651553(601767,707718) | 11949.004(10754.04,13341.188) | -0.827(-0.879,-0.775) |
| Croatia | 928237(846246,1003379) | 19908.482(18046.607,21673.095) | 642758(603296,678103) | 15189.784(13929.932,16394.918) | -0.566(-0.667,-0.464) |
| Uruguay | 794176(722087,871471) | 25911.635(23526.464,28455.094) | 639006(590670,689203) | 19739.686(18123.453,21404.906) | -1.157(-1.234,-1.081) |
| Senegal | 367323(335561,399650) | 4266.614(3945.043,4567.949) | 633517(586782,685415) | 3864.608(3622.016,4119.134) | -0.592(-0.652,-0.531) |
| Jordan | 244048(221162,266669) | 5827.745(5339.864,6305.377) | 620302(569824,677910) | 4737.579(4370.677,5152.632) | -0.800(-0.899,-0.701) |
| Taiwan (Province of China) | 823017(775809,874715) | 3976.014(3748.763,4206.812) | 606030(569494,647122) | 2396.237(2232.052,2583.482) | -1.927(-2.225,-1.628) |
| Singapore | 437423(399259,476114) | 13409.82(12197.941,14551.898) | 591023(538860,645004) | 11769.74(10559.888,12998.965) | -1.129(-1.282,-0.975) |
| Guinea | 299599(274974,325094) | 4426.748(4121.623,4735.66) | 587361(545404,629009) | 4075.23(3831.373,4320.992) | -0.405(-0.439,-0.371) |
| Benin | 241171(221236,261247) | 4401.298(4097.238,4705.461) | 576965(533466,624809) | 3998.022(3750.027,4252.892) | -0.480(-0.544,-0.416) |
| Ireland | 499939(455699,546466) | 14007.946(12759.661,15279.697) | 576483(529867,627504) | 12688.507(11490.538,14048.525) | -0.554(-0.672,-0.435) |
| Zimbabwe | 403919(367948,443695) | 3688.438(3423.559,3989.407) | 563317(523554,605959) | 3529.034(3312.802,3745.791) | -0.230(-0.327,-0.133) |
| Burundi | 284141(263345,306179) | 4749.42(4431.637,5053.781) | 554903(514282,602672) | 4009.951(3762.438,4274.715) | -0.650(-0.724,-0.575) |
| Kyrgyzstan | 492950(452031,534296) | 10220.171(9411.835,11019.727) | 535131(481569,586390) | 7470.417(6759.903,8162.575) | -1.296(-1.360,-1.232) |
| Paraguay | 355393(322483,393468) | 8163.075(7481.409,8912.329) | 527274(483259,575369) | 7127.452(6545.506,7780.915) | -0.621(-0.710,-0.532) |
| Democratic People's Republic of Korea | 511042(477364,546002) | 2461.942(2301.581,2635.002) | 526995(491907,565248) | 1975.245(1845.986,2116.138) | -0.573(-0.685,-0.461) |
| Rwanda | 343558(317312,372449) | 4396.947(4112.321,4710.76) | 488846(450020,532005) | 3556.585(3314.918,3827.355) | -0.944(-1.025,-0.863) |
| Nicaragua | 396604(349876,447224) | 8398.999(7577.861,9231.694) | 485957(432378,539291) | 7211.737(6474.692,7950.154) | -1.246(-1.569,-0.923) |
| Turkmenistan | 394361(358816,430861) | 9326.015(8513.742,10128.582) | 464059(417109,503605) | 8707.194(7833.504,9450.006) | -0.485(-0.536,-0.435) |
| Albania | 851488(766932,942429) | 24170.419(21879.658,26665.663) | 454677(416759,498030) | 18802.775(17017.803,20611.442) | -1.752(-1.931,-1.573) |
| El Salvador | 498973(443568,558178) | 8089.037(7298.724,8921.156) | 437439(391371,486979) | 6757.178(6039.131,7512.757) | -1.175(-1.344,-1.005) |
| Bosnia and Herzegovina | 1065694(963977,1175818) | 23342.431(21082.753,25755.83) | 436492(393624,474614) | 15444.002(13675.213,17182.782) | -1.932(-2.216,-1.647) |
| Georgia | 696296(642218,752038) | 12901.688(11872.675,13947.675) | 436141(413392,460458) | 13550.938(12761.003,14408.352) | 0.069(-0.059,0.198) |
| South Sudan | 291615(267576,318665) | 4573.587(4231.663,4916.72) | 424726(391901,460437) | 4128.704(3848.118,4411.6) | -0.280(-0.398,-0.161) |
| Slovenia | 511228(469120,552687) | 26192.708(23782.219,28529.9) | 409561(377561,441225) | 20512.575(18477.587,22634.422) | -0.443(-0.719,-0.166) |
| Mongolia | 259878(235376,282371) | 10964.623(10005.7,11846.859) | 391697(362826,419012) | 11455.296(10609.499,12251.527) | 0.037(-0.052,0.127) |
| Libya | 273634(250006,299158) | 6091.154(5615.205,6561.353) | 360061(333446,387948) | 5076.703(4696.284,5456.607) | -0.556(-0.667,-0.445) |
| Papua New Guinea | 133600(123783,146012) | 3264.262(3039.772,3495.489) | 358394(335864,382719) | 3526.741(3321.343,3746.514) | -0.397(-1.221,0.434) |
| Sierra Leone | 192702(176359,208223) | 4139.154(3857.813,4408.157) | 357664(331309,385124) | 3827.748(3600.175,4077.014) | -0.606(-0.883,-0.329) |
| Costa Rica | 294015(257383,330613) | 8900.467(7922.741,9853.806) | 344146(309257,378247) | 7837.268(6955.481,8733.547) | -1.012(-1.080,-0.943) |
| Republic of Moldova | 667902(622245,713723) | 15048.868(14030.044,16128.264) | 324040(300855,347594) | 10091.333(9227.201,10942.818) | -1.856(-1.946,-1.766) |
| Lithuania | 615895(574762,658054) | 17036.675(15793.602,18290.331) | 320276(300453,340650) | 12621.871(11625.198,13642.321) | -1.325(-1.512,-1.138) |
| Togo | 171118(156770,186015) | 4214.34(3915.304,4508.113) | 318295(294190,342105) | 3712.068(3476.899,3948.384) | -0.704(-0.759,-0.649) |
| Panama | 225708(201235,251737) | 8721.128(7841.079,9588.321) | 317756(285267,352649) | 7525.06(6753.134,8366.147) | -0.841(-0.914,-0.768) |
| Palestine | 141388(129341,154448) | 6360.091(5869.609,6878.799) | 304657(279731,329470) | 5487.447(5063.755,5869.184) | -0.374(-0.452,-0.295) |
| North Macedonia | 350279(311854,388691) | 17488.599(15558.177,19397.487) | 300668(273297,329557) | 15372.267(13668.062,17082.626) | -0.812(-0.926,-0.698) |
| Oman | 152674(140654,165389) | 7520.077(6981.585,8064.066) | 297018(273817,321359) | 6307.477(5876.599,6745.932) | -0.588(-0.749,-0.427) |
| Eritrea | 174051(160300,189034) | 4767.184(4422.214,5106.141) | 287959(266182,309145) | 4291.684(4007.027,4564.434) | -0.541(-0.596,-0.486) |
| Lebanon | 167396(152815,182612) | 5428.486(4978.634,5881.416) | 285952(262608,310831) | 5153.388(4693.818,5604.598) | -0.212(-0.280,-0.144) |
| Kuwait | 121608(111967,131465) | 6367.222(5864.166,6872.399) | 277646(255989,302254) | 5825.942(5373.091,6303.908) | -0.471(-0.678,-0.263) |
| Lao People's Democratic Republic | 195312(179988,211181) | 4502.093(4184.073,4837.083) | 271631(252903,291537) | 3607.211(3363.292,3849.288) | -0.745(-0.807,-0.683) |
| Qatar | 42636(38962,46840) | 8316.832(7717.556,8940.787) | 245047(224343,267070) | 7237.14(6764.261,7722.525) | -0.016(-0.222,0.190) |
| Armenia | 500860(459106,543802) | 14123.38(12967.205,15318.736) | 233957(214449,253381) | 8557.152(7795.74,9310.343) | -2.115(-2.233,-1.996) |
| Puerto Rico | 248360(224079,272192) | 6885.435(6218.121,7554.128) | 214754(199694,230138) | 7250.896(6608.982,7946.445) | -0.075(-0.281,0.132) |
| Latvia | 494204(462351,524852) | 19283.309(17963.457,20621.253) | 214715(201032,228314) | 12741.798(11743.336,13766.322) | -1.942(-2.113,-1.770) |
| Central African Republic | 118965(110068,127839) | 4105.981(3843.056,4371.094) | 208391(194284,223655) | 3662.256(3448.882,3884.934) | -0.437(-0.494,-0.380) |
| Jamaica | 209102(188020,231914) | 8390.358(7619.383,9193.725) | 208381(189965,227176) | 7503.381(6809.535,8192.851) | -0.822(-0.965,-0.678) |
| Liberia | 116208(106604,125747) | 4224.779(3920.444,4510.285) | 204239(189055,221856) | 3653.176(3422.792,3908.958) | -0.776(-0.808,-0.744) |
| Congo | 97790(89628,106269) | 3886.489(3636.729,4146.055) | 174671(161498,189595) | 3231.065(3012.831,3461.835) | -0.798(-0.842,-0.755) |
| Cyprus | 113164(103606,122452) | 14784.114(13518.26,16012.934) | 167092(154651,180532) | 13136.536(11939.439,14439.321) | -0.533(-0.677,-0.388) |
| Mauritania | 90598(83065,98495) | 4135.249(3858.072,4422.59) | 161920(148926,177113) | 3522.475(3279.526,3775.898) | -0.603(-0.666,-0.539) |
| Estonia | 272378(254641,290258) | 18079.249(16795.238,19316.558) | 141722(131755,150794) | 12116.502(11082.693,13154.924) | -1.817(-1.940,-1.694) |
| Botswana | 61855(57048,67369) | 4420.149(4117.974,4731.261) | 100162(93605,107045) | 4091.816(3836.634,4351.029) | -0.541(-0.655,-0.427) |
| Gambia | 45436(41398,49797) | 4177.354(3873.931,4491.903) | 96148(88314,104147) | 3890.553(3628.354,4154.492) | -0.545(-0.653,-0.436) |
| Namibia | 57567(52903,62389) | 3855.838(3573.566,4139.444) | 91690(85314,99011) | 3629.09(3393.721,3891.651) | -0.306(-0.451,-0.160) |
| Montenegro | 114050(102261,125301) | 18236.117(16302.953,20072.392) | 88444(80695,96004) | 15717.682(13955.335,17378.379) | -0.780(-0.846,-0.715) |
| Luxembourg | 63800(59741,68252) | 17372.158(16074.934,18730.393) | 85416(79905,91373) | 13981.038(12910.656,15278.59) | -0.746(-0.805,-0.686) |
| Guinea-Bissau | 52877(48795,57326) | 4774.41(4470.225,5084.884) | 83875(77850,89656) | 3965.86(3715.49,4197.37) | -0.895(-0.961,-0.828) |
| Bahrain | 29342(26710,31923) | 5288.027(4843.343,5714.034) | 81038(74680,87763) | 5186.454(4747.695,5615.74) | -0.211(-0.354,-0.068) |
| Lesotho | 61430(56618,66885) | 3699.782(3444.626,3945.77) | 74148(69210,79312) | 3780.492(3564.505,4013.162) | 0.118(0.047,0.190) |
| Trinidad and Tobago | 76852(69949,84626) | 6048.61(5544.926,6607.456) | 71232(65178,77319) | 5616.681(5095.806,6184.889) | -0.658(-0.756,-0.561) |
| Gabon | 43016(39816,46569) | 4210.347(3952.63,4501.005) | 64491(59785,69829) | 3556.124(3334.606,3807.429) | -0.635(-0.673,-0.596) |
| Guyana | 57798(53364,62844) | 6910.55(6432.653,7416.247) | 61702(57610,65683) | 7985.013(7470.469,8487.628) | 0.125(-0.017,0.268) |
| Brunei Darussalam | 38356(34857,41724) | 13799.334(12640.804,14957.655) | 55867(51434,60251) | 12159.608(11125.706,13135.092) | -0.560(-0.595,-0.524) |
| Timor-Leste | 36206(33013,39191) | 4453.784(4096.431,4789.227) | 54053(50229,58378) | 3772.667(3537.858,4037.506) | -0.829(-0.949,-0.710) |
| Malta | 51798(47795,56648) | 14584.135(13366.9,16032.207) | 51711(48068,55372) | 13385.989(12193.269,14722.532) | -0.550(-0.639,-0.462) |
| Djibouti | 18874(17374,20621) | 4269.716(3961.55,4599.366) | 49697(46224,53490) | 3944.909(3690.47,4220.824) | -0.648(-0.883,-0.412) |
| Equatorial Guinea | 19863(18469,21598) | 4368.219(4093.011,4668.721) | 49063(44872,53998) | 3185.713(2965.62,3439.553) | -1.291(-1.328,-1.255) |
| Eswatini | 34216(31377,37323) | 3904.036(3639.407,4187.878) | 46908(43652,50522) | 3847.422(3612.028,4110.195) | -0.127(-0.253,-0.001) |
| Bhutan | 35770(32760,38792) | 5443.088(5035.359,5813.404) | 45018(42422,47971) | 5996.061(5654.031,6342.496) | -0.089(-0.468,0.291) |
| Mauritius | 47270(43284,51507) | 4084.296(3745.133,4434.774) | 43000(40060,46331) | 3529.826(3251.29,3833.17) | -0.576(-0.669,-0.483) |
| Iceland | 36793(33532,40086) | 14495.337(13183.434,15835.407) | 41815(38459,45420) | 12692.431(11539.158,14010.638) | -0.587(-0.685,-0.489) |
| Suriname | 24437(21989,26719) | 5933.433(5393.829,6432.455) | 36268(33346,39243) | 6391.054(5858.868,6935.924) | 0.009(-0.101,0.118) |
| Belize | 14763(13317,16328) | 7091.949(6474.385,7738.557) | 35460(32398,38811) | 7906.029(7278.169,8596.463) | 0.054(-0.146,0.255) |
| Solomon Islands | 14661(13617,15801) | 4625.732(4328.338,4913.299) | 33539(31619,35605) | 5188.689(4912.227,5484.033) | 0.585(0.255,0.916) |
| Comoros | 22150(20270,24098) | 4486.41(4162.782,4812.376) | 28823(26851,31240) | 3817.634(3573.278,4098.876) | -0.726(-0.790,-0.662) |
| Fiji | 26151(24069,28607) | 3292.373(3055.545,3560.304) | 26977(25079,29146) | 2936.401(2735.834,3156.275) | -0.657(-0.828,-0.485) |
| Maldives | 13150(11949,14330) | 5778.782(5314.118,6233.101) | 24099(22260,26158) | 4521.103(4193.34,4865.861) | -0.702(-1.233,-0.168) |
| Cabo Verde | 17903(16462,19435) | 4553.685(4248.179,4881.729) | 22941(21420,24576) | 4151.51(3880.949,4439.733) | -0.668(-0.711,-0.625) |
| Bahamas | 15603(13966,17287) | 5730.416(5198.465,6289.353) | 21391(19413,23433) | 5748.999(5181.286,6305.335) | 0.021(-0.342,0.385) |
| Barbados | 15043(13529,16602) | 5983.752(5381.053,6612.635) | 15941(14655,17228) | 6066.447(5489.615,6645.168) | -0.418(-0.575,-0.260) |
| Andorra | 7913(7281,8630) | 14961.345(13689.2,16325.029) | 12796(12080,13680) | 15080.972(13991.849,16361.717) | 0.083(0.032,0.135) |
| Sao Tome and Principe | 6862(6237,7500) | 4925.027(4556.403,5311.415) | 11597(10726,12419) | 5248.584(4910.763,5580.994) | -0.343(-0.421,-0.265) |
| Saint Lucia | 9579(8633,10583) | 6519.156(5923.008,7108.521) | 11481(10645,12345) | 6961.346(6423.558,7528.34) | -0.273(-0.429,-0.117) |
| Vanuatu | 5098(4671,5613) | 3311.585(3057.945,3567.768) | 9486(8852,10284) | 3020.367(2832.788,3240.268) | -0.474(-0.943,-0.003) |
| Antigua and Barbuda | 5909(5353,6465) | 9430.834(8619.101,10234.915) | 8752(8025,9470) | 10199.544(9314.778,11092.964) | -0.162(-0.451,0.127) |
| Grenada | 7189(6591,7841) | 7935.844(7302.909,8621.836) | 8546(7879,9238) | 8423.02(7764.078,9111.44) | 0.058(-0.117,0.234) |
| Saint Vincent and the Grenadines | 8235(7452,8993) | 6957.662(6393.814,7524.467) | 8105(7495,8683) | 7342.912(6766.486,7893.82) | -0.240(-0.399,-0.081) |
| Greenland | 8810(8222,9402) | 15891.386(14976.488,16851.258) | 7342(6942,7722) | 12632.009(11877.397,13344.003) | -0.713(-0.835,-0.591) |
| Samoa | 6864(6335,7430) | 3876.424(3619.397,4137.078) | 6884(6357,7545) | 3263.306(3042.931,3527.545) | -0.274(-1.341,0.805) |
| United States Virgin Islands | 7183(6517,7849) | 6711.354(6091.907,7311.259) | 4931(4521,5310) | 6427.73(5801.461,7081.786) | -0.657(-0.809,-0.503) |
| Saint Kitts and Nevis | 3616(3309,3914) | 8493.284(7851.425,9139.778) | 4902(4516,5304) | 8873.106(8148.068,9577.815) | -0.051(-0.252,0.151) |
| Guam | 4974(4521,5458) | 3534.302(3233.608,3858.398) | 4811(4468,5166) | 3100.109(2861.947,3351.503) | -0.637(-0.735,-0.539) |
| Monaco | 3238(3006,3494) | 12428.189(11222.426,13707.692) | 3954(3662,4256) | 12135.359(10938.774,13487.065) | -0.036(-0.123,0.051) |
| Seychelles | 3640(3338,3959) | 4864.984(4480.805,5250.135) | 3897(3621,4191) | 3759.939(3470.863,4057.809) | -0.990(-1.117,-0.862) |
| Dominica | 4485(4058,4936) | 5845.107(5331.732,6388.136) | 3798(3464,4112) | 5920.998(5393.819,6428.891) | 0.007(-0.381,0.397) |
| San Marino | 2914(2661,3164) | 13030.671(11822.653,14343.701) | 3689(3413,3952) | 12663.531(11521.341,13966.883) | -0.196(-0.253,-0.138) |
| Bermuda | 3624(3278,3955) | 6414.919(5747.217,7089.57) | 3487(3220,3757) | 6538.977(5893.062,7223.529) | -0.293(-0.414,-0.173) |
| Micronesia (Federated States of) | 3611(3312,3944) | 3451.529(3211.497,3699.153) | 3427(3208,3693) | 3328.389(3133.122,3561.662) | -0.212(-0.813,0.392) |
| Tonga | 3285(2971,3627) | 3208.5(2946.207,3490.529) | 2966(2702,3240) | 2776.276(2555.204,3019.029) | -0.421(-0.776,-0.063) |
| Kiribati | 1865(1714,2054) | 2414.785(2228.961,2623.808) | 2751(2532,3031) | 2205.072(2041.033,2406.469) | -0.294(-0.439,-0.149) |
| Northern Mariana Islands | 2465(2278,2672) | 5208.442(4847.417,5597.056) | 2121(2003,2265) | 4549.833(4279.663,4849.738) | -0.908(-1.035,-0.782) |
| Marshall Islands | 1605(1469,1765) | 3497.526(3253.339,3769.17) | 1824(1705,1963) | 3209.983(3017.727,3436.803) | -0.315(-0.390,-0.239) |
| American Samoa | 2052(1886,2237) | 4177.301(3870.246,4505.739) | 1814(1689,1957) | 3719.933(3480.427,3994.616) | -0.368(-1.231,0.503) |
| Palau | 876(819,939) | 5685.608(5346.575,6053.568) | 1046(991,1106) | 5955.112(5625.549,6329.359) | -0.001(-0.041,0.038) |
| Cook Islands | 720(660,790) | 3667.221(3385.447,3985.266) | 569(525,627) | 3325.345(3047.505,3688.632) | -0.480(-1.335,0.383) |
| Nauru | 368(339,400) | 3591.004(3350.118,3859.598) | 399(373,431) | 3605.162(3389.229,3863.429) | -0.072(-0.150,0.005) |
| Tuvalu | 311(290,336) | 3272.011(3050.756,3525.915) | 384(359,415) | 3101.227(2909.616,3340.299) | -1.789(-2.454,-1.119) |
| Niue | 82(75,88) | 3536.151(3272.014,3821.258) | 54(51,58) | 3312.23(3093.509,3546.281) | -0.422(-0.984,0.143) |
| Tokelau | 54(49,59) | 3342.553(3081.478,3621.231) | 43(40,46) | 3107.469(2895.912,3352.988) | -0.263(-0.333,-0.193) |

Abbreviations: EAPC, estimated annual percentage change; UI, uncertainty interval.

^a^ EAPC is expressed as 95% confidence interval.
