# Supplementary material for: Global, regional, national burden and trends of unintentional injuries from 1990 to 2021 and projections to 2035: a systematic analysis of the Global Burden of Disease study 2021
Source: Front Public Health. 2025 Sep 3;13:1653491. doi: 10.3389/fpubh.2025.1653491 (PMC12442766; doi:10.3389/fpubh.2025.1653491)
Supplement: Supplementary file 9 [file Table_2.docx]

**Table S2:** Unintentional injury deaths and ASRs per 100,000 cases, by 204 countries and territories, 1990 and 2021, and EAPC per 100,000 ASRs, 1990-2021

| Characteristic | Number in 1990 | Age-standardized Rate in 1990 (95% CI) | Number in 2021 | Age-standardized Rate in 2021 (95% CI) | EAPC (Death rates) |
| --- | --- | --- | --- | --- | --- |
| India | 360233(303753,404529) | 360232.962(303753.271,404528.788) | 460492(387389,516699) | 41.468(34.785,46.393) | -0.880(-0.945,-0.814) |
| China | 418551(372491,479431) | 46.18(41.535,50.603) | 295758(217833,366371) | 19.059(14.462,23.111) | -1.614(-1.817,-1.410) |
| United States of America | 39741(37391,40988) | 46.635(39.787,54.507) | 79952(70265,84890) | 14.806(13.388,15.578) | 1.622(1.489,1.755) |
| Pakistan | 32469(26720,37810) | 32469.047(26720.305,37810.459) | 49741(40515,59269) | 30.459(24.197,36.971) | -1.004(-1.714,-0.288) |
| Russian Federation | 65643(64849,66533) | 58.052(46.627,70.56) | 48667(44958,52105) | 25.492(23.612,27.221) | -2.012(-2.857,-1.159) |
| Nigeria | 30348(25822,34807) | 30348.235(25821.843,34806.9) | 47438(28686,61439) | 30.06(21.838,37.531) | -1.443(-1.644,-1.241) |
| Brazil | 30816(29759,31829) | 30816.319(29758.53,31828.853) | 42351(39201,44300) | 18.063(16.657,18.923) | -0.171(-0.418,0.076) |
| Japan | 16276(15409,16743) | 48.959(37.722,62.644) | 41051(33716,45105) | 9.968(8.797,10.616) | 2.651(2.219,3.085) |
| Indonesia | 39389(32109,44654) | 30.659(28.499,32.729) | 38860(31461,46987) | 18.674(14.76,21.943) | -1.419(-2.565,-0.260) |
| Viet Nam | 30680(25508,36573) | 51.708(50.194,53.173) | 32218(24763,39150) | 36.175(26.738,44.168) | -0.962(-1.120,-0.804) |
| Germany | 18307(16835,19292) | 48.237(41.924,55.855) | 28494(24285,30961) | 12.982(11.471,13.889) | 1.799(1.125,2.477) |
| France | 25526(23529,26684) | 73.187(52.906,111.751) | 27592(23644,29792) | 16.69(14.864,17.76) | -0.072(-0.331,0.188) |
| Ethiopia | 29609(22429,38458) | 29608.745(22429.474,38458.205) | 24198(19671,32101) | 39.972(33.149,50.756) | -3.431(-3.581,-3.282) |
| Bangladesh | 61268(51737,70822) | 61268.262(51737.477,70822.485) | 22584(18301,27209) | 16.156(13.191,19.698) | -5.200(-5.937,-4.458) |
| Democratic Republic of the Congo | 16794(13574,20605) | 16794.145(13573.885,20605.252) | 18919(14262,25448) | 34.112(26.132,44.45) | -2.133(-2.318,-1.947) |
| Mexico | 23876(23130,24805) | 23876.273(23129.714,24804.614) | 17659(15795,19615) | 14.377(12.87,15.958) | -2.161(-2.321,-2.000) |
| Thailand | 14267(12436,16202) | 25.945(24.55,27.09) | 16174(12629,20136) | 19.394(15.608,23.687) | -0.387(-0.767,-0.006) |
| Italy | 12320(11158,12950) | 28.957(24.271,34.71) | 15362(12494,16831) | 8.72(7.429,9.387) | 0.394(0.158,0.630) |
| Myanmar | 22873(17924,28353) | 35.324(34.274,36.327) | 15107(12310,18791) | 30.971(25.253,37.907) | -2.423(-3.894,-0.930) |
| Philippines | 14989(13944,16253) | 47.687(39.033,52.953) | 14927(12837,16989) | 16.023(13.851,18.221) | -1.061(-1.713,-0.404) |
| United Kingdom | 8287(7812,8525) | 44.342(37.932,53.005) | 13507(11870,14336) | 9.829(8.858,10.32) | 1.363(1.169,1.558) |
| Ukraine | 22394(21630,24479) | 41.463(34.657,48.949) | 11308(8243,14636) | 20.309(14.976,25.964) | -3.058(-3.846,-2.265) |
| Afghanistan | 7002(5387,8924) | 7002.206(5386.891,8924.003) | 11268(8788,14396) | 49.426(37.877,62.733) | -2.440(-2.881,-1.997) |
| South Africa | 10325(9154,11284) | 10325.477(9154.44,11284.311) | 10973(9990,11920) | 20.71(18.981,22.475) | -1.187(-1.441,-0.932) |
| United Republic of Tanzania | 9426(8065,10988) | 9425.851(8064.793,10987.878) | 10764(8360,13788) | 30.973(25.109,37.84) | -1.970(-2.058,-1.882) |
| Iran (Islamic Republic of) | 57572(53407,62181) | 57572.413(53407.167,62181.183) | 9910(9060,10977) | 12.341(11.22,13.925) | -3.631(-4.654,-2.598) |
| Canada | 4474(4168,4668) | 62.931(51.278,76.773) | 9677(8320,10437) | 13.072(11.551,13.958) | 1.828(1.683,1.974) |
| Turkey | 12560(10975,14370) | 12559.731(10974.748,14370.39) | 9648(7925,11331) | 11.819(9.635,13.892) | -2.158(-2.868,-1.443) |
| Spain | 5889(5642,6065) | 40.537(34.85,46.605) | 9232(7898,10064) | 8.693(7.767,9.3) | 1.304(1.079,1.529) |
| Poland | 11408(11096,11669) | 76.153(58.594,97.185) | 9219(8383,9952) | 14.837(13.654,16.002) | -0.768(-0.888,-0.647) |
| Republic of Korea | 11461(10590,12167) | 43.672(32.808,57.393) | 9170(7638,10529) | 11.148(9.476,12.823) | -1.267(-1.593,-0.940) |
| Egypt | 12538(10799,13876) | 12538.434(10798.78,13876.274) | 8969(7439,10659) | 11.25(9.291,13.429) | -2.690(-2.912,-2.468) |
| Iraq | 8887(7342,10730) | 8887.037(7342.438,10730.19) | 8849(6592,11814) | 24.531(18.941,32.052) | -2.841(-3.073,-2.608) |
| Kenya | 5180(4219,7001) | 5179.791(4219.279,7001.023) | 8499(6922,10618) | 35.384(28.514,43.701) | -0.353(-0.548,-0.157) |
| Sudan | 12211(9384,15300) | 12211.364(9383.512,15299.581) | 8429(5847,11276) | 23.032(16.575,30.451) | -3.592(-3.703,-3.481) |
| Nepal | 8406(6892,10083) | 8405.802(6891.721,10083.085) | 7971(6411,10153) | 34.689(27.77,45.647) | -1.378(-2.002,-0.750) |
| Uganda | 5300(4294,6792) | 5299.635(4294.09,6791.795) | 7589(5807,9936) | 32.393(26.091,40.356) | -1.903(-2.040,-1.766) |
| Niger | 4887(3826,6096) | 4886.72(3825.662,6096.405) | 7205(4495,10920) | 42.507(29.492,62.036) | -2.489(-2.708,-2.269) |
| Somalia | 3810(2714,5427) | 3809.88(2714.228,5427.155) | 7110(4539,12269) | 62.034(41.56,101.997) | -1.177(-1.544,-0.809) |
| Mozambique | 6044(4709,7526) | 6044.308(4708.902,7525.888) | 6965(4689,9947) | 42.282(29.279,58.598) | -1.813(-2.012,-1.614) |
| Mali | 4396(3374,5488) | 4395.552(3373.779,5487.687) | 6810(4584,8730) | 43.346(31.226,56.193) | -1.655(-1.822,-1.487) |
| Haiti | 4693(3740,5599) | 4693.472(3740.464,5598.992) | 6787(5742,7851) | 64.104(55.112,73.975) | -1.419(-4.192,1.434) |
| Burkina Faso | 4591(3741,5458) | 4590.574(3741.289,5458.135) | 6718(4518,9225) | 42.636(31.425,53.036) | -1.338(-1.470,-1.205) |
| Angola | 6329(5094,7837) | 6329.334(5093.623,7837.286) | 6627(4836,8694) | 32.647(24.473,41.931) | -3.621(-3.829,-3.412) |
| Argentina | 9457(9143,9704) | 44.624(35.855,53.989) | 6549(6142,6849) | 12.714(11.977,13.281) | -1.994(-2.147,-1.841) |
| Peru | 10190(8254,11243) | 10190.341(8254.188,11243.428) | 6537(5121,8282) | 18.577(14.497,23.581) | -3.321(-3.762,-2.878) |
| Cameroon | 3142(2577,3680) | 3142.331(2577.272,3680.472) | 6425(4151,8376) | 33.185(24.606,43.504) | -1.067(-1.314,-0.820) |
| Netherlands | 2425(2178,2578) | 27.931(23.358,32.526) | 6418(5396,7010) | 16.549(14.096,18.005) | 3.130(2.677,3.585) |
| Saudi Arabia | 6228(5206,7533) | 6227.601(5206.114,7533.472) | 6307(4708,8337) | 18.282(14.626,23.019) | -1.822(-2.150,-1.494) |
| Chad | 2389(1887,2932) | 2388.727(1886.985,2931.549) | 6143(4386,7700) | 47.381(36.179,60.925) | -0.334(-0.564,-0.104) |
| Yemen | 5676(4072,7404) | 5676.29(4072.346,7403.833) | 6073(4182,8181) | 24.217(17.436,31.984) | -2.838(-2.960,-2.715) |
| Côte d'Ivoire | 3617(2963,4402) | 3616.722(2963.196,4401.592) | 5942(4148,7884) | 35.41(27.339,45.105) | -0.816(-0.994,-0.637) |
| Ghana | 3519(2923,4258) | 3519.331(2923.063,4257.68) | 5810(4318,7452) | 30.374(23.906,37.78) | -0.629(-0.810,-0.447) |
| Morocco | 7592(6037,9064) | 7592.06(6037.403,9063.797) | 5760(4454,7270) | 16.897(13.318,21.133) | -2.077(-2.182,-1.973) |
| Democratic People's Republic of Korea | 4666(3433,6500) | 70.987(59.84,82.149) | 5628(4264,7465) | 20.57(15.87,26.977) | -0.041(-0.166,0.085) |
| Australia | 2095(1978,2179) | 82.761(65.552,111.113) | 5399(4572,5898) | 11.017(9.569,11.901) | 2.412(2.193,2.631) |
| Venezuela (Bolivarian Republic of) | 4121(4006,4242) | 4120.989(4006.413,4241.906) | 5203(4063,6486) | 19.719(15.474,24.548) | -0.913(-2.386,0.583) |
| Algeria | 5987(4988,7104) | 5987.349(4987.84,7103.522) | 5150(4147,6257) | 14.221(12.017,16.971) | -2.317(-2.580,-2.053) |
| Madagascar | 4013(3419,4611) | 4012.92(3418.899,4611.101) | 5082(4022,6437) | 31.454(25.359,39.528) | -1.850(-1.950,-1.750) |
| Colombia | 6602(6346,6857) | 6601.745(6346.374,6857.155) | 5014(4189,5843) | 9.492(7.904,11.087) | -2.162(-2.464,-1.858) |
| Cuba | 2963(2834,3056) | 29.576(25.616,33.97) | 4964(4361,5543) | 24.95(22.005,27.825) | 1.321(0.994,1.650) |
| Romania | 8085(7813,8340) | 47.449(40.938,53.444) | 4938(4402,5430) | 17.296(15.466,18.969) | -1.432(-1.719,-1.144) |
| Cambodia | 4564(3766,5407) | 31.245(29.842,32.78) | 4925(3832,6187) | 39.331(31.515,47.697) | -1.550(-1.739,-1.361) |
| Uzbekistan | 6306(5777,6892) | 20.874(17.136,24.932) | 4561(3950,5221) | 13.498(11.731,15.443) | -3.053(-3.374,-2.730) |
| Belgium | 2545(2353,2668) | 20.143(19.481,20.787) | 4286(3618,4672) | 16.632(14.659,17.844) | 1.878(1.573,2.184) |
| Sri Lanka | 5617(5057,6107) | 23.975(23.126,24.72) | 4160(2870,5472) | 17.774(12.561,23.135) | -2.222(-3.669,-0.753) |
| Taiwan (Province of China) | 5933(5827,6044) | 11.955(11.433,12.503) | 4152(3760,4422) | 11.604(10.624,12.265) | -1.794(-2.315,-1.270) |
| Malawi | 4711(3876,5746) | 4711.31(3875.658,5746.207) | 4143(3057,5529) | 41.081(32.298,51.659) | -2.738(-2.872,-2.604) |
| Zimbabwe | 1910(1552,2243) | 1909.938(1551.864,2243.038) | 4088(3185,5169) | 35.278(27.764,43.784) | 1.963(1.543,2.384) |
| Malaysia | 2802(2515,3065) | 26.834(25.738,27.847) | 4064(3621,4503) | 14.613(12.761,16.317) | -0.918(-1.170,-0.664) |
| Kazakhstan | 6629(6247,6983) | 20.909(18.147,24.208) | 4031(3640,4446) | 21.196(19.143,23.3) | -2.398(-3.065,-1.725) |
| Guinea | 2923(2327,3559) | 2923.258(2326.79,3558.8) | 3782(2578,5097) | 39.921(29.392,53.405) | -1.336(-1.590,-1.080) |
| Zambia | 3191(2704,3656) | 3191.055(2703.872,3656.066) | 3580(2678,4612) | 36.66(29.104,45.401) | -2.603(-2.801,-2.403) |
| Belarus | 4363(4187,4797) | 37.065(29.679,44.055) | 3564(2943,4270) | 27.561(22.839,32.689) | -1.296(-2.018,-0.568) |
| South Sudan | 2566(2011,3240) | 2566.401(2011.342,3239.981) | 3540(2651,4753) | 53.291(40.541,71.166) | -0.454(-0.913,0.008) |
| Guatemala | 3281(3145,3432) | 3281.107(3144.895,3431.851) | 3472(3013,3970) | 25.543(22.177,29.182) | -1.348(-1.884,-0.809) |
| Senegal | 2840(2357,3390) | 2840.459(2356.758,3389.512) | 3468(2569,4580) | 35.196(26.689,46.223) | -1.563(-1.751,-1.374) |
| Benin | 2002(1592,2358) | 2001.75(1592.02,2357.552) | 3349(2116,4430) | 35.112(24.929,44.618) | -1.440(-1.601,-1.279) |
| Ecuador | 2931(2827,3034) | 2930.551(2826.691,3033.688) | 3209(2662,3848) | 19.418(16.19,23.165) | -1.686(-1.906,-1.466) |
| Chile | 3869(3771,3963) | 35.833(29.757,43.355) | 3089(2874,3241) | 13.268(12.452,13.913) | -1.549(-1.979,-1.117) |
| Burundi | 2626(2111,3227) | 2625.685(2111.062,3226.71) | 3026(2139,4740) | 43.145(32.144,63.7) | -2.230(-2.346,-2.113) |
| Czechia | 4963(4684,5254) | 28.226(22.722,33.465) | 2962(2593,3312) | 15.027(13.282,16.823) | -1.864(-2.150,-1.578) |
| Greece | 1692(1605,1752) | 43.525(34.253,56.07) | 2836(2521,3032) | 11.591(10.667,12.192) | 1.980(1.364,2.599) |
| Hungary | 5806(5477,6103) | 26.571(23.05,30.11) | 2747(2400,3116) | 15.511(13.589,17.624) | -2.543(-2.758,-2.328) |
| Portugal | 2055(1984,2121) | 56.462(47.343,65.203) | 2736(2391,2949) | 10.716(9.715,11.431) | 0.911(0.366,1.458) |
| Rwanda | 3423(2866,4003) | 3422.506(2866.259,4002.75) | 2676(2034,3485) | 36.283(27.978,45.829) | -3.569(-3.899,-3.238) |
| Bolivia (Plurinational State of) | 3459(2953,4066) | 3459.067(2952.874,4065.83) | 2632(2084,3317) | 25.824(20.427,32.672) | -2.948(-3.108,-2.787) |
| Sweden | 2005(1845,2103) | 22.438(19.09,26.28) | 2620(2231,2916) | 10.503(9.056,11.646) | 0.627(0.404,0.851) |
| Switzerland | 2320(2110,2453) | 43.16(35.055,53.131) | 2535(2065,2803) | 11.604(9.84,12.584) | 0.170(-0.292,0.635) |
| Papua New Guinea | 1168(852,1483) | 26.637(25.728,27.4) | 2518(1980,3171) | 29.816(22.065,40.723) | -0.799(-1.445,-0.148) |
| Austria | 2103(1962,2202) | 58.029(48.931,73.156) | 2502(2161,2713) | 12.585(11.214,13.511) | 0.818(0.433,1.204) |
| Sierra Leone | 1931(1549,2284) | 1930.707(1549.421,2283.736) | 2311(1495,3154) | 36.784(26.494,47.702) | -1.802(-2.171,-1.431) |
| Central African Republic | 1681(1374,2027) | 1681.272(1374.248,2026.836) | 2096(1547,2723) | 52.162(40.086,66.724) | -1.367(-1.452,-1.283) |
| Finland | 1590(1489,1654) | 34.287(29.548,37.154) | 1963(1700,2119) | 15.271(13.708,16.235) | 0.367(0.251,0.484) |
| Eritrea | 1412(1135,1714) | 1412.429(1134.812,1714.23) | 1841(1338,2535) | 50.082(38.337,64.325) | -1.214(-1.310,-1.117) |
| Tajikistan | 1755(1530,1957) | 23.197(18.963,28.377) | 1788(1443,2158) | 17.911(14.554,21.366) | -2.891(-3.357,-2.422) |
| Togo | 1046(866,1231) | 1045.862(865.812,1230.701) | 1758(1167,2381) | 35.396(25.578,46.112) | -0.886(-1.041,-0.730) |
| Dominican Republic | 1723(1492,1980) | 46.119(37.935,54.261) | 1724(1415,2085) | 16.364(13.452,19.791) | -0.942(-1.295,-0.588) |
| Tunisia | 2057(1670,2436) | 2057.252(1670.105,2436.438) | 1678(1251,2216) | 13.993(10.568,18.381) | -1.743(-1.812,-1.674) |
| Honduras | 1465(1233,1646) | 1465.076(1232.725,1646.374) | 1652(1290,2099) | 22.168(18.199,27.311) | -2.858(-4.440,-1.250) |
| Norway | 1259(1151,1326) | 44.931(38.276,52.304) | 1632(1374,1776) | 14.037(12.113,15.12) | 0.217(0.086,0.349) |
| Croatia | 1908(1804,2025) | 20.372(16.807,24.316) | 1435(1263,1593) | 16.427(14.613,18.161) | 0.044(-0.293,0.381) |
| Bulgaria | 2472(2329,2630) | 49.017(41.048,58.992) | 1424(1226,1645) | 14.033(12.101,16.228) | -1.429(-1.634,-1.224) |
| Lao People's Democratic Republic | 1860(1441,2352) | 34.419(26.771,38.132) | 1404(1076,1791) | 24.174(18.942,30.094) | -2.851(-2.949,-2.752) |
| Slovakia | 1690(1440,1858) | 60.652(51.217,68.429) | 1332(1127,1527) | 16.757(14.298,19.168) | -0.591(-0.796,-0.386) |
| Denmark | 1703(1552,1800) | 66.81(57.497,77.736) | 1269(1088,1364) | 10.233(8.977,10.926) | -1.962(-2.281,-1.643) |
| Azerbaijan | 1929(1721,2139) | 102.675(95.544,110.467) | 1243(1031,1505) | 12.257(10.34,14.808) | -3.063(-3.310,-2.817) |
| Serbia | 1688(1499,1897) | 55.009(35.664,74.725) | 1242(1033,1433) | 8.699(7.339,10.008) | -0.745(-0.826,-0.664) |
| Libya | 790(664,926) | 789.957(663.679,925.948) | 1238(923,1664) | 20.258(14.88,27.66) | 0.209(-0.126,0.545) |
| Syrian Arab Republic | 2201(1822,2624) | 2201.268(1822.164,2624.396) | 1204(938,1530) | 10.111(7.979,12.577) | -2.401(-2.917,-1.882) |
| Israel | 799(758,827) | 28.859(22.187,37.558) | 1202(1035,1300) | 9.219(8.122,9.908) | -0.750(-1.050,-0.448) |
| Liberia | 1117(897,1322) | 1117.039(897.322,1322.028) | 1130(723,1651) | 34.55(24.056,47.239) | -2.749(-3.053,-2.445) |
| Republic of Moldova | 1953(1876,2032) | 48.109(39.56,60.161) | 1129(1024,1255) | 23.665(21.244,26.527) | -1.284(-1.708,-0.857) |
| Lithuania | 1890(1826,1984) | 49.222(42.022,58.027) | 1082(969,1184) | 24.682(22.363,26.826) | -1.347(-1.817,-0.875) |
| Paraguay | 696(603,811) | 696.241(602.709,811.369) | 1079(824,1350) | 17.339(13.303,21.785) | -0.278(-0.552,-0.002) |
| Congo | 865(724,1027) | 864.839(723.719,1027.465) | 1008(809,1263) | 30.096(24.795,36.478) | -2.382(-2.637,-2.126) |
| Georgia | 1654(1566,1745) | 54.166(43.654,67.251) | 977(884,1072) | 20.71(18.844,22.61) | -0.421(-0.943,0.103) |
| El Salvador | 1375(1151,1506) | 1374.804(1151.08,1505.845) | 972(786,1177) | 14.859(12.003,18.007) | -1.667(-2.270,-1.060) |
| Kyrgyzstan | 1515(1406,1630) | 17.002(15.855,18.039) | 954(829,1088) | 14.994(12.967,17.134) | -3.496(-3.733,-3.259) |
| Lebanon | 841(716,1009) | 841.47(715.656,1008.706) | 933(799,1072) | 14.895(12.77,17.168) | -1.363(-1.580,-1.145) |
| New Zealand | 488(457,509) | 40.068(30.657,55.044) | 920(796,994) | 11.487(10.199,12.314) | 1.551(1.181,1.923) |
| Uruguay | 1170(1132,1201) | 47.109(37.354,56.215) | 906(830,957) | 17.887(16.664,18.826) | -1.194(-1.542,-0.845) |
| Slovenia | 646(610,677) | 53.174(44.189,63.974) | 871(740,965) | 17.85(15.4,19.806) | 0.612(0.309,0.914) |
| United Arab Emirates | 432(338,549) | 431.767(337.529,548.997) | 855(680,1047) | 18.034(14.965,21.398) | -2.739(-3.022,-2.454) |
| Turkmenistan | 1297(1190,1409) | 32.195(26.126,38.735) | 843(687,1051) | 16.861(13.702,20.972) | -2.929(-3.219,-2.639) |
| Mongolia | 1097(964,1240) | 35.177(29.87,42.21) | 838(701,981) | 26.601(22.311,31.051) | -2.050(-2.245,-1.855) |
| Jordan | 609(534,690) | 608.835(534.141,689.655) | 836(702,995) | 8.839(7.441,10.514) | -3.214(-3.449,-2.978) |
| Costa Rica | 580(555,602) | 580.313(554.968,602.477) | 801(713,879) | 14.939(13.333,16.36) | -0.631(-0.940,-0.322) |
| Puerto Rico | 670(645,691) | 670.337(645.046,691.079) | 705(587,814) | 10.118(8.522,11.626) | 0.960(-0.332,2.269) |
| Mauritania | 521(442,597) | 521.072(442.083,596.719) | 701(527,888) | 26.617(20.201,34.064) | -1.542(-1.734,-1.349) |
| Latvia | 1603(1550,1668) | 70.498(58.163,83.516) | 700(629,769) | 23.835(21.512,26.119) | -2.665(-3.147,-2.180) |
| Nicaragua | 895(749,988) | 895.056(749.326,988.087) | 661(576,769) | 11.93(10.373,13.855) | -2.952(-4.123,-1.766) |
| Lesotho | 362(284,463) | 362.458(284.418,462.582) | 572(449,704) | 37.11(29.237,45.792) | 1.688(1.264,2.113) |
| Guinea-Bissau | 521(410,666) | 520.513(409.879,665.846) | 556(405,695) | 47.721(37.38,56.724) | -1.906(-2.159,-1.653) |
| Ireland | 592(563,615) | 33.479(24.868,45.301) | 548(473,592) | 7.204(6.34,7.754) | -1.140(-1.297,-0.983) |
| Gambia | 301(235,374) | 300.803(234.955,374.457) | 538(406,690) | 40.201(29.765,49.943) | -1.215(-1.478,-0.951) |
| Panama | 516(488,542) | 516.069(488.497,542.396) | 470(385,558) | 10.803(8.853,12.85) | -1.970(-2.188,-1.751) |
| Namibia | 308(256,370) | 307.552(256.331,370.073) | 460(344,626) | 23.852(18.242,31.138) | -0.358(-0.638,-0.077) |
| Armenia | 1117(1048,1196) | 26.054(23.412,29.585) | 459(403,515) | 13.218(11.539,14.844) | -3.069(-3.437,-2.699) |
| Bosnia and Herzegovina | 782(693,871) | 34.771(28.879,41.651) | 402(316,490) | 7.525(5.937,9.049) | -0.597(-0.859,-0.335) |
| Botswana | 330(251,443) | 330.095(250.752,443.448) | 399(313,504) | 19.944(16.166,24.72) | -1.203(-1.401,-1.004) |
| Palestine | 320(263,376) | 319.953(262.741,376.152) | 376(311,424) | 10.932(9.27,12.396) | -2.252(-2.409,-2.095) |
| Oman | 351(273,452) | 351.411(272.585,451.883) | 365(301,432) | 11.949(9.792,14.073) | -1.891(-2.200,-1.582) |
| Estonia | 872(836,945) | 62.21(52.101,74.111) | 357(316,393) | 17.127(15.258,18.869) | -3.432(-3.933,-2.929) |
| Singapore | 245(237,252) | 63.932(53.144,76.881) | 331(299,352) | 4.349(3.942,4.625) | -1.369(-1.528,-1.210) |
| Gabon | 306(256,357) | 306.128(255.772,357.487) | 324(242,425) | 27.572(21.355,35.708) | -1.631(-1.727,-1.535) |
| Jamaica | 253(241,266) | 252.586(240.843,265.774) | 315(251,397) | 9.814(7.806,12.421) | -0.172(-0.618,0.276) |
| Kuwait | 213(200,226) | 213.334(200.401,226.138) | 305(257,363) | 8.593(7.206,10.258) | -2.123(-2.490,-1.755) |
| Equatorial Guinea | 219(173,272) | 218.799(173.265,271.541) | 302(233,399) | 32.151(25.453,41.634) | -4.936(-5.507,-4.361) |
| Albania | 646(573,743) | 24.096(18.585,30.825) | 300(238,377) | 9.443(7.601,11.588) | -2.189(-2.470,-1.906) |
| Eswatini | 214(172,263) | 214.305(172.405,263.498) | 299(214,391) | 32.017(23.47,41.734) | 0.396(0.010,0.783) |
| Timor-Leste | 277(223,347) | 29.169(24.376,31.657) | 294(245,349) | 26.191(21.665,31.15) | -2.095(-2.557,-1.632) |
| Qatar | 87(72,104) | 86.853(72.317,104.492) | 271(207,350) | 13.879(11.056,17.414) | -2.087(-2.467,-1.706) |
| North Macedonia | 463(349,527) | 37.273(29.582,47.725) | 263(221,315) | 10.832(9.027,12.734) | -2.641(-2.913,-2.368) |
| Djibouti | 107(83,139) | 106.978(82.582,138.948) | 256(182,368) | 37.084(27.42,51.897) | -1.113(-1.688,-0.535) |
| Cyprus | 216(194,242) | 54.219(42.185,69.802) | 248(211,285) | 15.394(13.03,17.648) | -1.499(-1.685,-1.312) |
| Trinidad and Tobago | 252(242,262) | 251.632(241.877,261.936) | 243(192,302) | 15.159(12.011,18.685) | -0.691(-0.991,-0.390) |
| Bhutan | 223(135,295) | 223.336(135.291,294.6) | 237(163,318) | 38.626(26.306,51.63) | -0.940(-1.425,-0.453) |
| Guyana | 280(247,308) | 279.924(247.482,307.689) | 226(180,283) | 35.025(28.178,43.406) | 0.116(-0.174,0.407) |
| Mauritius | 185(179,191) | 184.68(178.783,190.554) | 201(187,209) | 12.997(12.09,13.496) | -0.420(-0.718,-0.121) |
| Comoros | 158(114,205) | 158.039(114.182,204.513) | 183(144,229) | 35.968(28.128,45.538) | -1.211(-1.450,-0.972) |
| Fiji | 158(136,187) | 35.043(29.162,39.655) | 173(138,215) | 22.413(18.121,27.579) | -0.328(-0.557,-0.097) |
| Solomon Islands | 86(57,117) | 20.105(17.649,23.262) | 166(128,205) | 32.261(25.337,40.659) | 0.195(-0.146,0.537) |
| Luxembourg | 99(94,103) | 30.518(26.647,33.787) | 148(127,163) | 12.918(11.335,14.245) | -0.026(-0.266,0.215) |
| Cabo Verde | 71(60,82) | 70.978(59.555,82.45) | 125(102,151) | 25.588(20.725,30.665) | -0.154(-0.571,0.265) |
| Suriname | 114(85,125) | 113.539(85.336,124.856) | 124(99,149) | 21.553(17.275,25.972) | -0.726(-1.058,-0.393) |
| Montenegro | 97(83,110) | 44.035(32.033,62.284) | 95(82,110) | 12.147(10.541,13.923) | -0.112(-0.317,0.092) |
| Bahrain | 60(55,66) | 60.338(55.091,65.703) | 93(80,107) | 9.147(7.818,10.477) | -2.608(-2.864,-2.351) |
| Malta | 59(55,62) | 34.587(27.689,43.259) | 92(78,102) | 9.982(8.773,11.08) | 0.959(0.785,1.133) |
| Belize | 59(55,63) | 39.056(33.388,45.102) | 77(70,85) | 21.81(19.629,24.05) | -1.807(-2.111,-1.503) |
| Bahamas | 70(66,75) | 35.545(30.628,40.972) | 73(60,90) | 19.71(16.2,24.19) | -0.046(-1.326,1.251) |
| Iceland | 39(36,42) | 55.546(47.969,64.739) | 72(60,80) | 11.53(9.848,12.824) | 0.737(-0.081,1.562) |
| Vanuatu | 32(24,40) | 18.722(16.641,20.458) | 65(53,77) | 27.711(23.075,32.618) | -0.248(-0.787,0.293) |
| Brunei Darussalam | 42(37,47) | 62.977(51.654,79.757) | 53(47,59) | 15.123(13.248,16.814) | -0.747(-1.094,-0.398) |
| Maldives | 72(61,86) | 56.218(49.355,65.38) | 52(43,62) | 12.793(10.814,14.84) | -3.680(-4.464,-2.889) |
| Barbados | 52(49,54) | 58.338(47.279,73.054) | 52(43,63) | 12.39(9.999,15.156) | -0.645(-0.976,-0.313) |
| Saint Lucia | 33(31,35) | 33.001(31.144,34.988) | 37(31,43) | 18.347(15.562,21.295) | -0.615(-1.101,-0.126) |
| Sao Tome and Principe | 31(26,36) | 30.505(25.879,36.5) | 35(29,42) | 26.365(22.576,31.108) | -2.148(-2.389,-1.906) |
| Samoa | 38(33,44) | 25.347(24.31,26.129) | 34(27,42) | 20.822(16.668,25.49) | -0.277(-1.539,1.001) |
| Saint Vincent and the Grenadines | 28(26,30) | 28.159(25.949,30.236) | 29(26,32) | 24.03(21.406,26.876) | 0.019(-0.330,0.369) |
| Grenada | 35(32,38) | 38.998(33.152,45.187) | 27(24,30) | 26.976(23.842,29.818) | -1.341(-1.761,-0.920) |
| Micronesia (Federated States of) | 27(22,33) | 24.647(23.434,25.756) | 23(18,30) | 28.015(22.095,35.131) | -0.455(-1.171,0.266) |
| Tonga | 19(16,23) | 27.964(23.492,32.632) | 19(15,24) | 20.497(16.377,25.983) | 0.029(-0.287,0.346) |
| Greenland | 27(22,31) | 26.736(21.864,30.857) | 18(14,21) | 33.044(25.869,39.625) | -1.310(-1.549,-1.070) |
| Seychelles | 22(19,24) | 22.141(19.15,24.052) | 17(15,19) | 15.018(13.429,16.903) | -1.440(-1.727,-1.153) |
| Antigua and Barbuda | 17(16,18) | 56.203(46.507,68.848) | 16(15,17) | 17.971(17.128,18.664) | -1.583(-2.291,-0.870) |
| United States Virgin Islands | 21(19,24) | 21.076(18.601,23.845) | 15(12,18) | 14.596(11.812,17.581) | -0.156(-0.559,0.248) |
| Dominica | 18(16,20) | 43.691(36.811,51.755) | 14(12,17) | 20.358(17.395,23.89) | 0.648(-0.821,2.138) |
| Guam | 16(14,17) | 15.55(13.833,16.985) | 14(13,16) | 8.105(7.138,9.074) | -0.445(-0.754,-0.135) |
| Marshall Islands | 10(9,12) | 25.464(21.353,27.598) | 13(10,17) | 29.097(22.801,36.368) | 0.282(0.096,0.467) |
| Monaco | 10(8,12) | 9.843(7.635,11.678) | 13(10,15) | 14.111(11.521,16.787) | 0.109(0.028,0.191) |
| American Samoa | 11(9,12) | 10.813(9.352,12.321) | 12(10,14) | 26.998(22.569,31.994) | 0.348(-0.581,1.285) |
| Kiribati | 9(7,11) | 36.611(35.716,37.517) | 11(8,13) | 11.247(8.879,13.909) | -0.885(-0.953,-0.817) |
| Andorra | 4(3,6) | 45.132(39.016,52.016) | 11(8,15) | 6.16(4.353,8.397) | 1.671(1.299,2.044) |
| Bermuda | 11(11,12) | 11.476(10.864,12.244) | 11(9,13) | 9.148(7.907,10.892) | -0.348(-0.962,0.270) |
| Northern Mariana Islands | 9(6,11) | 8.795(6.338,11.492) | 10(9,11) | 22.978(20.044,25.111) | 0.439(0.100,0.779) |
| Saint Kitts and Nevis | 12(12,13) | 12.417(11.707,13.089) | 10(9,12) | 18.91(16.239,21.729) | -1.892(-2.289,-1.494) |
| Palau | 7(6,9) | 7.087(5.602,8.834) | 9(7,10) | 52.2(43.581,62.572) | 0.239(0.112,0.366) |
| San Marino | 3(3,4) | 3.258(2.775,3.74) | 4(3,5) | 4.492(3.013,6.049) | 0.496(0.062,0.932) |
| Nauru | 3(2,3) | 2.801(2.267,3.388) | 3(2,4) | 33.049(25.824,41.609) | -0.221(-0.693,0.254) |
| Tuvalu | 3(2,3) | 2.842(2.346,3.345) | 3(2,3) | 24.725(19.685,30.099) | -1.350(-2.792,0.113) |
| Cook Islands | 3(3,4) | 3.148(2.802,3.579) | 2(1,2) | 8.296(6.517,10.117) | -1.395(-3.053,0.291) |
| Niue | 1(0,1) | 0.556(0.451,0.687) | 0(0,1) | 28.841(25.259,33.43) | -0.221(-1.120,0.687) |
| Tokelau | 0(0,0) | 0.296(0.229,0.373) | 0(0,0) | 24.293(19.281,29.397) | 0.006(-0.341,0.354) |

Abbreviations: EAPC, estimated annual percentage change; UI, uncertainty interval.

^a^ EAPC is expressed as 95% confidence interval.
