# Supplementary material for: Global, regional, national burden and trends of unintentional injuries from 1990 to 2021 and projections to 2035: a systematic analysis of the Global Burden of Disease study 2021
Source: Front Public Health. 2025 Sep 3;13:1653491. doi: 10.3389/fpubh.2025.1653491 (PMC12442766; doi:10.3389/fpubh.2025.1653491)
Supplement: Supplementary file 10 [file Table_3.docx]

**Table S3:** Number of DALYs and ASRs per 100,000 unintentional injuries by 204 countries and territories, 1990 and 2021, and EAPC per 100,000 ASRs, 1990-2021

| Characteristic | Number in 1990 | Age-standardized Rate in 1990 (95% CI) | Number in 2021 | Age-standardized Rate in 2021 (95% CI) | EAPC (DALYs rates) |
| --- | --- | --- | --- | --- | --- |
| India | 23196893(20148380,26094722) | 2937.776(2583.777,3293.503) | 22176660(18846933,25315796) | 1725.495(1472.027,1965.03) | -1.876(-1.971,-1.780) |
| China | 30963292(27447502,35089716) | 2775.574(2464.727,3146.148) | 16201728(13388808,19553188) | 1089.777(925.02,1288.01) | -2.853(-3.068,-2.638) |
| United States of America | 3242035(2665824,3939874) | 1190.746(989.939,1435.636) | 4348104(3595176,5372494) | 972.071(814.792,1175.79) | 0.182(-0.054,0.420) |
| Nigeria | 2254459(1907622,2583983) | 2106.787(1819.925,2418.344) | 3482355(2143332,4434796) | 1444.397(980.585,1796.649) | -1.424(-1.643,-1.204) |
| Russian Federation | 5130209(4548832,5882159) | 3286.112(2939.749,3739.808) | 3325654(2826075,4015415) | 1903.374(1635.968,2268.107) | -2.133(-2.812,-1.450) |
| Pakistan | 2190991(1826729,2570659) | 1836.612(1552.051,2102.191) | 3179140(2697452,3673620) | 1426.666(1204.951,1657.918) | -1.003(-1.728,-0.272) |
| Brazil | 2858625(2540590,3254603) | 2062.436(1812.373,2368.591) | 2795353(2393388,3303731) | 1193.035(1024.959,1401.3) | -1.438(-1.505,-1.371) |
| Indonesia | 3103549(2676552,3507230) | 1730.987(1483.921,1960.437) | 2638080(2257570,3053961) | 1029.116(880.532,1179.193) | -1.789(-2.838,-0.728) |
| Japan | 1542382(1205434,1952924) | 1101.245(880.665,1377.211) | 1779446(1433958,2222500) | 794.482(625.819,1005.685) | 0.296(0.039,0.554) |
| Bangladesh | 4975194(4197754,5741407) | 3264.56(2794.845,3717.267) | 1617523(1387985,1888785) | 1032.325(884.14,1205.863) | -5.543(-6.203,-4.878) |
| Viet Nam | 1995650(1701483,2338279) | 2872.293(2451.321,3376.678) | 1613344(1354806,1884861) | 1669.915(1395.118,1941.283) | -1.816(-2.002,-1.629) |
| Mexico | 2205857(1978430,2475991) | 2768.045(2452.974,3144.065) | 1532235(1281896,1820806) | 1188.298(998.544,1414.077) | -1.953(-2.198,-1.708) |
| Ethiopia | 2073718(1567445,2603041) | 3852.868(3060.201,4944.72) | 1513019(1214967,2033099) | 1620.547(1350.854,2072.787) | -3.761(-3.876,-3.645) |
| Germany | 1253536(1000569,1549185) | 1290.908(1040.634,1592.449) | 1404461(1113435,1757675) | 995.241(769.648,1274.443) | 0.368(0.198,0.537) |
| France | 1273292(1066286,1530141) | 1873.501(1575.762,2236.624) | 1329086(1059683,1662736) | 1283.686(1006.711,1609.979) | -0.129(-0.222,-0.035) |
| Democratic Republic of the Congo | 1298019(1046008,1574676) | 2665.273(2230.286,3245.995) | 1255236(965487,1717595) | 1540.486(1218.015,2006.29) | -2.548(-2.781,-2.314) |
| Philippines | 1165439(1073385,1264023) | 1763.547(1616.865,1936.562) | 1053096(926277,1202654) | 979.246(860.178,1120.23) | -1.408(-1.990,-0.822) |
| Myanmar | 1680829(1304110,2053327) | 3921.18(3110.51,4720.75) | 1017744(864806,1219552) | 1876.532(1600.204,2231.424) | -2.616(-3.976,-1.236) |
| Ukraine | 1755832(1551162,2043656) | 3175.585(2828.005,3656.548) | 970355(749524,1211697) | 1855.433(1458.993,2275.744) | -2.375(-2.891,-1.856) |
| Italy | 1008158(781889,1276598) | 1456.606(1139.011,1840.627) | 935299(718246,1203808) | 955.195(710.412,1261.708) | -0.588(-0.688,-0.487) |
| Thailand | 1011802(891056,1151564) | 1850.304(1636.198,2099.736) | 884350(744221,1062284) | 1191.297(1018.53,1411.67) | -1.179(-1.505,-0.853) |
| United Kingdom | 731450(571700,917630) | 1085.563(855.484,1362.807) | 838600(650968,1067998) | 866.764(663.138,1112.143) | 0.049(-0.030,0.128) |
| Afghanistan | 474451(358615,601163) | 4272.063(3318.079,5374.615) | 802410(639626,1014696) | 2569.37(2068.449,3183.299) | -2.307(-2.776,-1.836) |
| Iran (Islamic Republic of) | 4203896(3905710,4536551) | 6380.865(5937.24,6855.731) | 768944(669239,915305) | 883.596(771.812,1043.111) | -3.746(-4.650,-2.834) |
| Egypt | 1013984(872206,1127397) | 1742.338(1537.872,1925.252) | 767866(654176,913171) | 779.576(667.578,929.633) | -2.539(-2.741,-2.336) |
| Republic of Korea | 991704(858206,1166210) | 2382.681(2072.22,2798.454) | 725820(563648,929375) | 994.891(777.756,1274.334) | -1.669(-1.915,-1.421) |
| United Republic of Tanzania | 688817(584593,795855) | 2325.885(2042.367,2658.816) | 721477(557625,945252) | 1359.432(1099.032,1699.499) | -2.148(-2.272,-2.023) |
| Poland | 962439(807435,1152231) | 2430.637(2051.864,2893.939) | 702413(550419,882959) | 1360.388(1072.617,1713.437) | -1.000(-1.101,-0.898) |
| South Africa | 755533(682751,829178) | 2002.597(1812.183,2207.597) | 696119(632430,771977) | 1232.409(1116.426,1370.383) | -1.624(-1.836,-1.410) |
| Saudi Arabia | 492643(417503,580683) | 3394.758(2888.771,3988.703) | 670624(537499,841451) | 1690.226(1359.106,2098.818) | -1.130(-1.413,-0.847) |
| Iraq | 670363(561549,780245) | 3693.216(3069.249,4335.144) | 660642(535007,838932) | 1661.634(1351.514,2093.55) | -2.827(-3.054,-2.599) |
| Turkey | 953535(826864,1092954) | 1685.253(1471.545,1927.907) | 652782(544831,787819) | 758.305(641.89,905.813) | -2.597(-3.149,-2.042) |
| Sudan | 966572(734610,1220608) | 3884.616(3089.715,4716.386) | 650313(490724,843845) | 1523.802(1175.045,1955.209) | -3.667(-3.788,-3.545) |
| Spain | 528881(426634,651267) | 1257.503(1030.821,1534.189) | 614365(474673,787303) | 913.451(699.416,1185.909) | 0.062(-0.009,0.133) |
| Argentina | 739395(647623,862298) | 2269.893(1986.263,2649.299) | 607500(485487,760575) | 1254.957(1015.723,1562.964) | -1.612(-1.704,-1.521) |
| Haiti | 386405(307294,456935) | 4938.41(4076.619,5684.81) | 579517(500876,666297) | 4391.002(3855.812,5023.842) | -0.751(-3.342,1.910) |
| Niger | 396852(309295,493991) | 3421.586(2769.795,4224.606) | 536240(343023,793396) | 1987.032(1376.178,2885.984) | -2.780(-3.011,-2.548) |
| Uganda | 383240(308821,475164) | 1968.04(1648.393,2498.881) | 524547(390047,677345) | 1373.201(1108.732,1711.765) | -1.930(-2.084,-1.776) |
| Canada | 323782(264976,398387) | 1107.637(915.966,1351.368) | 491717(396191,613116) | 865.744(700.191,1075.709) | 0.563(0.475,0.652) |
| Mali | 331814(248962,416982) | 2967.123(2390.331,3654.751) | 487628(340344,612981) | 1972.597(1442.892,2490.251) | -1.825(-2.014,-1.635) |
| Somalia | 275067(195556,378505) | 3315.815(2509.725,4682.011) | 485956(320164,803990) | 2591.905(1821.699,4192.482) | -1.276(-1.673,-0.878) |
| Burkina Faso | 336367(267416,407719) | 2736.026(2309.163,3200.145) | 475029(321339,664383) | 1913.02(1391.473,2518.342) | -1.390(-1.538,-1.243) |
| Angola | 499842(404898,621450) | 3657.823(3021.875,4384.953) | 474255(347406,624544) | 1508.044(1168.229,1905.667) | -3.890(-4.150,-3.628) |
| Nepal | 614155(497862,745202) | 2932.555(2467.857,3478.081) | 473469(397466,563159) | 1671.284(1404.632,1975.144) | -2.124(-2.701,-1.545) |
| Kenya | 350470(297334,442451) | 1652.483(1382.06,2194.686) | 472151(389972,578879) | 1259.109(1043.583,1508.386) | -0.917(-1.148,-0.686) |
| Peru | 820428(666645,915269) | 3421.23(2820.101,3806.619) | 471427(391010,574443) | 1313.8(1087.742,1602.076) | -3.650(-3.997,-3.302) |
| Uzbekistan | 575847(516499,644623) | 2512.197(2242.819,2829.913) | 463298(395626,543947) | 1329.531(1136.441,1567.3) | -2.610(-2.816,-2.403) |
| Mozambique | 453240(356138,543164) | 2790.115(2249.645,3415.053) | 462650(324845,651647) | 1734.846(1272.937,2363.355) | -2.251(-2.440,-2.062) |
| Chad | 176514(133843,219965) | 2294.107(1851.033,2766.235) | 455821(327872,574305) | 2268.597(1739.355,2841.253) | -0.282(-0.502,-0.061) |
| Colombia | 677846(599972,769457) | 2152.689(1879.524,2468.417) | 454710(374103,560435) | 890.287(732.949,1097.398) | -2.406(-2.591,-2.222) |
| Australia | 279003(212642,359354) | 1576.613(1211.611,2020.028) | 448102(337501,585415) | 1308.953(969.942,1723.437) | 0.323(0.235,0.411) |
| Yemen | 444795(328007,570433) | 2754.15(2076.051,3593.338) | 446448(332289,571556) | 1423.22(1085.603,1793.318) | -2.988(-3.102,-2.873) |
| Cameroon | 227145(180984,270244) | 1948.694(1669.435,2244.311) | 445004(297934,564247) | 1509.75(1095.982,1893.05) | -1.127(-1.388,-0.865) |
| Venezuela (Bolivarian Republic of) | 397144(355575,448927) | 2157.938(1910.443,2471.173) | 431734(354275,527184) | 1638.17(1350.605,1990.421) | -1.118(-2.285,0.063) |
| Morocco | 605627(498118,722952) | 2313.919(1927.123,2712.036) | 422886(349560,528707) | 1150.6(953.108,1433.926) | -2.369(-2.476,-2.261) |
| Côte d'Ivoire | 272193(220498,331000) | 2080.822(1775.804,2482.477) | 406332(289394,539989) | 1572.266(1194.021,1989.048) | -1.067(-1.253,-0.880) |
| Romania | 786138(672959,933738) | 3349.747(2904.143,3936.318) | 401620(320137,501460) | 1652.32(1330.346,2051.338) | -1.771(-1.910,-1.632) |
| Algeria | 492203(422761,569816) | 1926.827(1663.208,2234.439) | 400377(336713,479335) | 937.536(791.339,1117.901) | -2.445(-2.646,-2.244) |
| Ghana | 249072(209902,296008) | 1655.799(1426.695,1948.654) | 357528(270824,459014) | 1238.199(976.256,1555.094) | -1.064(-1.236,-0.892) |
| Madagascar | 291700(249276,338483) | 2243.99(1952.893,2534.576) | 353950(286206,435480) | 1408.741(1167.56,1727.372) | -1.933(-2.058,-1.807) |
| Kazakhstan | 534361(487659,593300) | 3235.258(2946.054,3618.573) | 328113(286492,385569) | 1687.721(1475.245,1982.323) | -2.258(-2.729,-1.784) |
| Chile | 312110(277284,356208) | 2433.937(2147.276,2797.612) | 288073(225390,366456) | 1344.046(1055.806,1701.582) | -1.178(-1.497,-0.858) |
| Guatemala | 280899(257753,304800) | 3337.269(3052.141,3633.565) | 286381(248032,329349) | 1916.098(1652.413,2217.84) | -1.546(-1.980,-1.110) |
| Cambodia | 330589(273874,390515) | 2979.399(2508.949,3449.073) | 285858(231597,353545) | 1831.528(1504.164,2233.286) | -2.300(-2.464,-2.135) |
| Democratic People's Republic of Korea | 317803(237705,422528) | 1519.96(1150.792,2013.06) | 281481(217037,368757) | 1067.499(828.806,1390.029) | -1.053(-1.185,-0.922) |
| Zimbabwe | 134341(108420,157324) | 1424.635(1212.551,1640.92) | 272685(222629,337042) | 1848.527(1502.925,2264.791) | 1.895(1.512,2.280) |
| Sri Lanka | 361505(324287,399761) | 2200.194(1982.444,2430.477) | 267247(215122,333014) | 1132.953(920.511,1405.897) | -2.203(-3.547,-0.840) |
| Guinea | 220335(168463,271687) | 2712.442(2173.741,3237.035) | 261989(179968,342547) | 1899.857(1382.244,2471.404) | -1.583(-1.807,-1.358) |
| Malawi | 357906(290323,426642) | 2920.345(2461.745,3565.536) | 261902(194777,347133) | 1634.026(1285.315,2084.874) | -3.236(-3.368,-3.104) |
| Netherlands | 159186(124957,199329) | 935.348(739.529,1164.432) | 260960(209517,326073) | 932.935(737.051,1181.889) | 1.458(1.003,1.916) |
| Malaysia | 191868(172056,214491) | 1205.86(1082.514,1352.276) | 246630(219019,281889) | 790.788(702.145,902.422) | -1.257(-1.460,-1.054) |
| South Sudan | 182392(144564,225617) | 2771.249(2232.558,3497.436) | 245078(186102,319144) | 2446.168(1912.592,3177.758) | -0.399(-0.855,0.059) |
| Benin | 152183(119213,182364) | 2421.306(2016.831,2825.164) | 244807(159856,323418) | 1719.559(1206.984,2192.535) | -1.537(-1.713,-1.360) |
| Belarus | 328621(292473,376195) | 3059.235(2740.352,3477.198) | 240777(203122,297059) | 2052.357(1743.173,2516.657) | -1.329(-1.891,-0.763) |
| Zambia | 236113(199859,273696) | 2600.955(2266.408,2971.621) | 235285(180152,301273) | 1474.329(1189.408,1832.878) | -2.895(-3.115,-2.675) |
| Ecuador | 231759(213924,250849) | 2387.967(2191.54,2618.031) | 232782(199288,276012) | 1325.673(1135.661,1570.459) | -1.992(-2.158,-1.826) |
| Belgium | 177440(142260,219427) | 1502.921(1218.942,1844.343) | 232283(183704,292088) | 1311.878(1021.547,1654.944) | 0.803(0.592,1.014) |
| Czechia | 315615(261501,382149) | 2749.265(2287.227,3310.568) | 211686(165307,269491) | 1419.523(1101.272,1823.746) | -1.254(-1.363,-1.146) |
| Senegal | 212881(176766,255915) | 2299.782(1960.191,2715.696) | 209805(161555,277022) | 1493.625(1161.771,1925.303) | -2.225(-2.450,-1.999) |
| Burundi | 183741(147002,223887) | 2996.887(2489.978,3681.449) | 197694(140600,297436) | 1772.086(1347.406,2581.271) | -2.252(-2.453,-2.051) |
| Bolivia (Plurinational State of) | 275305(237192,320760) | 3797.106(3344.689,4366.962) | 192183(161464,234356) | 1697.416(1426.898,2067.931) | -3.256(-3.379,-3.134) |
| Papua New Guinea | 86017(67171,104508) | 2039.088(1588.725,2543.419) | 190758(158276,229360) | 1832.83(1528.335,2217.989) | -0.709(-1.333,-0.080) |
| Hungary | 327707(278425,389068) | 2750.87(2353.773,3237.498) | 186560(149536,234045) | 1363.764(1089.874,1718.442) | -1.848(-1.938,-1.757) |
| Cuba | 173486(154951,198087) | 1612.006(1442.097,1836.52) | 185585(156389,222274) | 1208.827(1019.253,1455.383) | 0.052(-0.127,0.232) |
| Taiwan (Province of China) | 365848(346396,392616) | 1864.733(1760.8,2005.793) | 175906(155338,200998) | 603.9(542.631,678.562) | -3.110(-3.518,-2.701) |
| Tajikistan | 160325(141854,179581) | 2661.704(2346.128,3034.471) | 167731(142381,200571) | 1615.455(1360.288,1930.43) | -2.615(-2.991,-2.238) |
| Sierra Leone | 148125(117512,177597) | 2648.263(2198.879,3110.874) | 163765(109358,220014) | 1813.037(1276.516,2392.408) | -2.018(-2.379,-1.656) |
| Rwanda | 241750(199400,284937) | 3112.38(2662.753,3621.898) | 163517(127547,210680) | 1455.327(1151.634,1834.152) | -3.944(-4.265,-3.621) |
| Switzerland | 155841(122850,195310) | 1818.576(1437.604,2282.647) | 160174(122963,205264) | 1160.62(872.534,1514.098) | -0.773(-0.820,-0.726) |
| Austria | 149122(118661,184750) | 1597.523(1279.489,1966.47) | 149553(116074,189765) | 1103.255(843.063,1418.066) | -0.177(-0.295,-0.059) |
| Sweden | 137489(106752,173684) | 1232.97(958.515,1549.894) | 147334(113761,187167) | 921.469(700.078,1187.247) | -0.247(-0.396,-0.098) |
| Central African Republic | 123154(99401,149352) | 3735.958(3110.536,4427.396) | 145902(109202,190418) | 2585.72(1990.349,3305.885) | -1.480(-1.599,-1.360) |
| Greece | 153352(120907,193151) | 1307.795(1048.921,1627.443) | 145153(115746,182155) | 959.22(755.388,1215.402) | -0.068(-0.158,0.022) |
| Bulgaria | 247656(205423,299429) | 2683.184(2265.049,3191.979) | 140089(111211,179494) | 1613.412(1291.507,2054.445) | -1.260(-1.339,-1.180) |
| Portugal | 168931(139610,203728) | 1587.314(1342.256,1898.107) | 135070(108437,167593) | 811.621(651.758,1020.112) | -0.916(-1.152,-0.679) |
| Dominican Republic | 148421(129892,169056) | 1947.722(1727.43,2222.962) | 133597(113232,158471) | 1217.168(1030.666,1444.116) | -1.326(-1.596,-1.055) |
| Honduras | 128589(112166,146033) | 2620.169(2285.439,2952.275) | 133109(109059,164146) | 1445.089(1201.402,1764.546) | -3.081(-4.492,-1.649) |
| Azerbaijan | 176214(156127,198725) | 2347.195(2081.246,2657.707) | 130553(109233,157366) | 1231.522(1036.781,1474.389) | -2.515(-2.700,-2.328) |
| Serbia | 182665(149792,227580) | 1822.968(1509.284,2244.156) | 129080(98807,170358) | 1111.568(849.88,1461.744) | -0.874(-0.935,-0.813) |
| Tunisia | 162493(137697,188600) | 1960.501(1658.316,2277.293) | 122900(99452,154858) | 995.565(810.39,1245.367) | -1.950(-2.028,-1.872) |
| Finland | 116233(95175,142323) | 1977.644(1629.005,2415.902) | 121350(94878,153498) | 1411.234(1091.633,1787.664) | -0.191(-0.534,0.154) |
| Eritrea | 101418(82229,123345) | 2939.185(2449.015,3429.749) | 117491(87948,158467) | 2055.466(1605.478,2677.2) | -1.572(-1.679,-1.465) |
| Slovakia | 139320(116352,169983) | 2527.971(2119.1,3065.733) | 116209(91555,148287) | 1653.572(1306.659,2103.908) | -0.553(-0.631,-0.476) |
| Togo | 80188(66022,95360) | 1970.252(1685.224,2298.78) | 112436(76234,148642) | 1520.418(1081.672,1964.91) | -1.426(-1.599,-1.254) |
| Syrian Arab Republic | 182192(153472,213943) | 1423.657(1222.915,1659.545) | 104396(84507,128119) | 752.637(614.065,919.346) | -2.146(-2.446,-1.845) |
| United Arab Emirates | 34296(28332,41431) | 2079.208(1726.368,2520.55) | 104351(84354,132378) | 1094.832(917.851,1314.352) | -1.537(-1.664,-1.410) |
| New Zealand | 65535(50147,84715) | 1838.56(1414.547,2366.699) | 95276(71347,124705) | 1526.453(1149.045,1997.419) | 0.099(-0.045,0.244) |
| Lao People's Democratic Republic | 138909(110041,175815) | 2926.352(2358.055,3619.426) | 94142(74868,116994) | 1341.9(1079.131,1648.48) | -3.202(-3.296,-3.108) |
| Israel | 59083(48842,72445) | 1226.487(1012.242,1505.522) | 87558(67777,112457) | 829.219(636.004,1073.884) | -0.687(-0.775,-0.600) |
| Croatia | 128546(107160,154275) | 2423.336(2034.682,2886.058) | 85036(66728,106643) | 1323.225(1031.288,1681.368) | -0.716(-0.877,-0.554) |
| Kyrgyzstan | 129695(118266,143815) | 2815.612(2554.55,3161.535) | 83566(72645,97603) | 1247.685(1080.949,1464.389) | -3.254(-3.409,-3.099) |
| Norway | 75415(60141,94027) | 1413.412(1128.568,1754.98) | 83134(65181,105189) | 1032.791(792.941,1328.336) | -0.405(-0.506,-0.304) |
| Libya | 61812(53135,71408) | 1552.963(1339.687,1790.706) | 82949(67491,103431) | 1220.426(996.807,1509.145) | -0.306(-0.542,-0.068) |
| Paraguay | 64182(55430,74320) | 1654.346(1425.703,1948.705) | 82728(69673,101164) | 1205.798(1015.514,1473.783) | -0.987(-1.183,-0.792) |
| Jordan | 50988(44983,56711) | 1420.022(1242.291,1612.217) | 79650(67027,96434) | 688.432(578.413,833.229) | -2.722(-2.895,-2.549) |
| Denmark | 90380(73547,111105) | 1364.339(1111.607,1674.393) | 77479(59747,99156) | 909.359(686.791,1174.58) | -1.100(-1.169,-1.030) |
| Liberia | 86106(67578,103988) | 2617.871(2148.644,3045.584) | 75943(49748,108781) | 1539.17(1073.627,2140.874) | -3.016(-3.366,-2.665) |
| Turkmenistan | 118212(106898,131861) | 2854.766(2571.634,3195.633) | 75288(63236,90138) | 1458.371(1222.003,1750.381) | -2.944(-3.181,-2.707) |
| Georgia | 142311(125730,165185) | 2584.594(2300.043,2987.43) | 73363(61347,88686) | 1773.285(1489.742,2124.625) | -0.775(-1.135,-0.414) |
| Republic of Moldova | 152594(137373,173061) | 3441.228(3103.038,3902.179) | 72656(62496,84591) | 1697.216(1454.895,1975.64) | -1.874(-2.130,-1.617) |
| El Salvador | 121105(105698,136122) | 2253.331(1982.402,2536.206) | 71917(60374,85960) | 1117.588(938.822,1335.978) | -2.276(-2.718,-1.833) |
| Mongolia | 89077(78139,101254) | 3893.458(3462.825,4397.337) | 69615(59498,81940) | 2093.858(1787.286,2477.891) | -1.963(-2.161,-1.765) |
| Lithuania | 136501(121703,156339) | 3556.121(3187.233,4041.895) | 65126(55554,78488) | 1769.916(1517.609,2117.707) | -1.816(-2.172,-1.458) |
| Nicaragua | 84913(74158,95779) | 2059.177(1804.453,2328.113) | 63741(53709,75871) | 1011.355(853.079,1198.403) | -2.954(-3.925,-1.972) |
| Congo | 60284(50698,72689) | 2417.824(2092.531,2829.397) | 62233(50169,77172) | 1319.592(1100.911,1589.844) | -2.728(-2.992,-2.464) |
| Uruguay | 88387(76810,103462) | 2782.124(2431.76,3239.535) | 60398(49004,74059) | 1500.678(1232.863,1842.778) | -1.683(-1.863,-1.502) |
| Ireland | 46568(37476,57675) | 1270.396(1026.114,1571.754) | 56419(42012,73842) | 914.382(677.277,1205.981) | -0.370(-0.498,-0.243) |
| Singapore | 31676(24979,40210) | 1063.227(840.291,1345.055) | 54024(39331,72324) | 740.616(542.424,987.223) | -0.412(-0.451,-0.373) |
| Costa Rica | 44861(39650,51097) | 1633.624(1434.269,1876.67) | 51867(43569,62064) | 1022.078(860.498,1223.747) | -1.129(-1.269,-0.988) |
| Slovenia | 54032(43785,66999) | 2454.945(2000.563,3035.388) | 51741(40423,66049) | 1608.947(1236.519,2083.928) | -0.091(-0.269,0.087) |
| Lebanon | 51057(43678,60153) | 1793.301(1543.911,2104.213) | 50067(42908,58932) | 848.898(729.415,1000.276) | -1.875(-2.032,-1.718) |
| Bosnia and Herzegovina | 97421(79470,121031) | 2117.906(1727.832,2626.926) | 48637(36581,63491) | 1133.188(851.435,1485.481) | -1.136(-1.351,-0.920) |
| Albania | 79502(67150,97324) | 2526.605(2105.714,3119.32) | 43289(33026,56667) | 1410.578(1090.857,1817.619) | -1.708(-1.868,-1.548) |
| Panama | 44082(39622,49432) | 1877.625(1667.035,2118.436) | 42795(35516,52035) | 999.118(829.145,1213.945) | -1.874(-2.017,-1.732) |
| Latvia | 112668(100066,128538) | 3997.051(3580.773,4502.069) | 42625(36070,51390) | 1733.418(1478.25,2078.29) | -2.867(-3.212,-2.521) |
| Mauritania | 35098(29407,40783) | 1663.139(1453.359,1880.848) | 41951(32416,52485) | 1099.894(874.622,1360.408) | -1.900(-2.166,-1.633) |
| Armenia | 107280(95443,121764) | 3148.377(2789.155,3591.837) | 39514(33561,47222) | 1208.587(1038.407,1436.275) | -3.256(-3.547,-2.965) |
| Puerto Rico | 44686(39166,51515) | 1248.709(1096.692,1437.828) | 37144(30439,46455) | 801.829(654.883,1007.901) | 0.031(-0.772,0.840) |
| Guinea-Bissau | 38621(30060,48725) | 3264.527(2661.01,4026.336) | 36826(27193,46315) | 2030.384(1573.359,2485.197) | -2.251(-2.547,-1.954) |
| Oman | 28939(23430,34762) | 1606.323(1306.115,1953.473) | 36800(30457,44676) | 862.789(711.581,1043.107) | -1.467(-1.695,-1.238) |
| Lesotho | 24752(20320,30284) | 1620.774(1314.003,2047.272) | 36055(29079,43004) | 2004.369(1615.236,2407.236) | 1.438(1.084,1.792) |
| Palestine | 26093(22024,30664) | 1344.179(1130.968,1597.209) | 35269(29556,41413) | 784.042(652.577,925.723) | -1.799(-1.938,-1.660) |
| Kuwait | 19023(16912,21708) | 1200.538(1056.794,1386.665) | 33450(27065,42423) | 698.508(571.724,869.08) | -1.401(-1.601,-1.201) |
| Gambia | 22118(17190,27601) | 2086.805(1689.605,2553.114) | 32869(25334,42209) | 1605.81(1260.459,2002.345) | -1.858(-2.132,-1.582) |
| North Macedonia | 44987(37183,54866) | 2267.897(1874.883,2755.657) | 32026(24528,41832) | 1223.916(944.794,1586.862) | -1.664(-1.767,-1.561) |
| Namibia | 21868(18433,25795) | 1622.665(1386.317,1932.336) | 30591(24066,40158) | 1330.61(1057.52,1711.345) | -0.479(-0.738,-0.218) |
| Qatar | 7352(6241,8729) | 1864.741(1595.016,2179.511) | 29373(23635,37186) | 971.499(786.098,1215.628) | -1.364(-1.631,-1.097) |
| Botswana | 23544(18802,30194) | 1890.572(1492.49,2477.101) | 29295(24466,35834) | 1270.38(1070.725,1538.759) | -1.046(-1.212,-0.881) |
| Jamaica | 24352(20897,28842) | 1097.429(932.79,1315.019) | 24667(19882,30642) | 825.318(666.548,1023.291) | -0.836(-1.053,-0.618) |
| Estonia | 61750(54966,70853) | 3757.046(3374.252,4264.531) | 24101(19800,29853) | 1411.845(1168.145,1736.356) | -3.253(-3.601,-2.904) |
| Eswatini | 15724(13044,18750) | 1938.604(1614.205,2358.504) | 19895(15062,24982) | 1788.88(1374.45,2264.271) | 0.048(-0.243,0.339) |
| Equatorial Guinea | 15952(12646,19762) | 3093.52(2536.406,3768.144) | 19894(15374,26208) | 1497.748(1199.693,1945.008) | -5.156(-5.655,-4.653) |
| Timor-Leste | 22383(18196,27786) | 2389.796(2010.527,2885.623) | 19469(16541,22837) | 1420.638(1213.26,1648.483) | -2.814(-3.255,-2.372) |
| Gabon | 19176(16091,22833) | 1970.467(1687.168,2291.306) | 19370(14451,25989) | 1217.683(948.724,1593.648) | -1.677(-1.822,-1.532) |
| Cyprus | 12918(10857,15543) | 1780.958(1515.72,2114.564) | 18343(14479,23280) | 1098.122(870.423,1389.716) | -0.718(-0.831,-0.604) |
| Djibouti | 8048(6421,10064) | 2038.981(1646.736,2560.366) | 15669(12017,21182) | 1490.477(1150.678,1995.144) | -1.749(-2.326,-1.168) |
| Trinidad and Tobago | 18589(16760,20641) | 1624.875(1460.554,1813.711) | 15225(12636,18465) | 1024.321(860.349,1239.324) | -1.119(-1.293,-0.945) |
| Guyana | 20637(18225,22961) | 2698.665(2407.803,2986.166) | 14588(12212,17443) | 1969.734(1653.095,2346.736) | -0.439(-0.668,-0.210) |
| Bhutan | 15015(9377,19274) | 2566.053(1741.841,3290.733) | 11738(9118,15072) | 1672.875(1303.379,2144.403) | -1.950(-2.448,-1.450) |
| Solomon Islands | 6207(4588,7814) | 2073.217(1514.784,2654.837) | 11733(9674,13953) | 1920.33(1587.64,2281.674) | 0.120(-0.211,0.451) |
| Fiji | 11182(9772,13028) | 1526.363(1342.178,1768.909) | 11518(9534,13924) | 1287.997(1070.673,1549.784) | -0.541(-0.766,-0.316) |
| Mauritius | 13368(12179,14846) | 1268.655(1150.255,1417.711) | 11400(10103,13143) | 791.378(704.731,904.399) | -1.071(-1.265,-0.877) |
| Comoros | 11135(8076,14147) | 2300.353(1741.575,2894.529) | 10074(8206,12392) | 1509.845(1242.028,1832.566) | -2.000(-2.217,-1.782) |
| Bahrain | 5134(4578,5805) | 1118.063(990.593,1269.392) | 9921(8106,12260) | 646.787(535.934,785.955) | -1.584(-1.708,-1.461) |
| Luxembourg | 7403(5964,9121) | 1666.739(1359.181,2037.048) | 9759(7555,12489) | 1112.728(851.569,1433.181) | -0.659(-0.790,-0.529) |
| Montenegro | 11172(8804,14126) | 1764.766(1399.552,2222.739) | 9695(7422,12554) | 1293.184(995.335,1670.06) | -0.495(-0.632,-0.357) |
| Suriname | 8201(6380,9273) | 2151.68(1713.579,2430.541) | 8018(6903,9416) | 1387.213(1196.806,1623.067) | -1.192(-1.427,-0.957) |
| Malta | 5178(4088,6507) | 1324.676(1052.027,1657.321) | 6773(5202,8679) | 1072.32(817.456,1391.218) | 0.456(0.341,0.572) |
| Cabo Verde | 4842(4037,5751) | 1341.894(1157.812,1545.838) | 6707(5647,7985) | 1262.935(1063.113,1499.555) | -0.969(-1.416,-0.521) |
| Belize | 4775(4381,5253) | 2307.439(2117.316,2550.68) | 5700(5049,6608) | 1394.821(1230.29,1621.128) | -2.048(-2.302,-1.793) |
| Brunei Darussalam | 3887(3358,4566) | 1741.329(1479.021,2065.964) | 5239(4262,6477) | 1189.088(978.36,1458.947) | -0.696(-0.875,-0.517) |
| Bahamas | 5096(4593,5646) | 2058.871(1855.431,2283.907) | 4902(4087,5902) | 1246.516(1043.178,1504.696) | -0.529(-1.584,0.538) |
| Iceland | 3345(2666,4194) | 1252.746(999.869,1571.756) | 4636(3617,5899) | 1018.579(788.483,1310.259) | -0.113(-0.563,0.340) |
| Maldives | 5774(4918,6764) | 2333.186(2032.838,2684.443) | 4244(3564,5148) | 866.27(738.246,1035.743) | -3.545(-4.260,-2.826) |
| Vanuatu | 2204(1757,2691) | 1609.084(1264.098,1975.815) | 4209(3540,4892) | 1456.553(1231.32,1690.059) | -0.427(-0.982,0.131) |
| Barbados | 3258(2889,3719) | 1253.756(1110.865,1432.284) | 2874(2353,3548) | 825.465(677.2,1021.434) | -0.951(-1.089,-0.812) |
| Sao Tome and Principe | 2246(1881,2728) | 1676.788(1431.376,1959.791) | 2293(1926,2749) | 1259.188(1079.248,1471.534) | -2.521(-2.815,-2.227) |
| Saint Lucia | 2385(2162,2635) | 1855.256(1681.91,2060.071) | 2288(1950,2728) | 1203.935(1031.405,1426.584) | -0.986(-1.278,-0.694) |
| Samoa | 2598(2249,2957) | 1591.194(1379.873,1833.044) | 2221(1860,2666) | 1142.36(960.078,1369.078) | -0.378(-1.609,0.869) |
| Saint Vincent and the Grenadines | 2125(1915,2350) | 2023.265(1837.779,2240.542) | 1738(1517,2036) | 1461.409(1278.453,1707.217) | -0.711(-0.962,-0.460) |
| Grenada | 2196(1983,2437) | 2581.179(2324.592,2868.362) | 1626(1422,1903) | 1538.262(1352.066,1791.528) | -1.284(-1.592,-0.975) |
| Micronesia (Federated States of) | 1775(1433,2122) | 1872.097(1538.816,2231.719) | 1447(1168,1766) | 1471.704(1201.173,1779.629) | -0.662(-1.379,0.061) |
| Andorra | 650(479,861) | 1137.476(846.907,1499.981) | 1413(1050,1871) | 1084.734(796.155,1438.008) | 0.996(0.867,1.125) |
| Antigua and Barbuda | 1144(1017,1310) | 1933.062(1713.978,2221.482) | 1240(1054,1479) | 1316.332(1125.864,1553.383) | -1.167(-1.746,-0.583) |
| Greenland | 1825(1563,2116) | 3575.75(3102.79,4125.283) | 1165(971,1397) | 1942.412(1644.891,2287.556) | -1.570(-1.765,-1.375) |
| Tonga | 1270(1093,1492) | 1380.617(1182.738,1621.513) | 1110(910,1361) | 1102.511(905.137,1350.797) | -0.337(-0.660,-0.014) |
| Guam | 1159(1034,1309) | 905.493(802.543,1023.099) | 1079(931,1281) | 640.778(554.949,756.922) | -0.582(-0.773,-0.391) |
| Seychelles | 1312(1169,1438) | 1908.605(1691.374,2110.848) | 1014(888,1158) | 884.856(781.724,1008.74) | -1.380(-1.653,-1.107) |
| United States Virgin Islands | 1580(1388,1814) | 1536.391(1356.178,1761.582) | 1012(837,1228) | 1099.347(923.684,1323.571) | -0.540(-0.788,-0.291) |
| Dominica | 1151(1013,1306) | 1632.614(1439.152,1848.202) | 911(788,1074) | 1366.22(1178.763,1606.719) | 0.515(-0.618,1.661) |
| Marshall Islands | 689(590,789) | 1766.026(1534.586,1992.379) | 842(683,1034) | 1550.459(1271.198,1896.573) | 0.037(-0.183,0.258) |
| Kiribati | 701(601,820) | 911.336(785.962,1053.465) | 812(675,977) | 700.291(590.968,837.765) | -0.990(-1.070,-0.910) |
| Saint Kitts and Nevis | 869(785,975) | 2216.042(1994.75,2505.936) | 793(656,975) | 1284.335(1077.651,1565.924) | -1.525(-1.778,-1.270) |
| American Samoa | 729(638,837) | 1642.748(1447.081,1866.285) | 686(584,804) | 1450.917(1232.509,1701.303) | -0.093(-1.010,0.833) |
| Monaco | 518(417,635) | 1259.213(1014.682,1563.869) | 628(505,782) | 1113.386(897.506,1390.193) | -0.092(-0.173,-0.012) |
| Northern Mariana Islands | 621(484,778) | 1452.757(1189.354,1772.529) | 599(523,686) | 1227.813(1078.659,1398.763) | -0.158(-0.406,0.090) |
| Bermuda | 748(657,869) | 1215.898(1073.348,1403.835) | 591(470,742) | 717.384(581.226,898.024) | -0.827(-1.131,-0.522) |
| Palau | 425(340,514) | 2925.701(2389.63,3523.704) | 467(396,553) | 2609.793(2228.223,3080.114) | -0.088(-0.189,0.012) |
| San Marino | 275(213,354) | 999.187(782.363,1280.814) | 402(294,529) | 809.984(585.682,1069.797) | 0.406(0.300,0.513) |
| Nauru | 190(157,228) | 1965.682(1633.077,2360.137) | 192(151,244) | 1814.961(1445.713,2269.055) | -0.283(-0.764,0.200) |
| Tuvalu | 189(155,223) | 1924.641(1607.067,2233.414) | 152(126,183) | 1279.902(1053.889,1532.43) | -2.848(-4.109,-1.571) |
| Cook Islands | 217(193,246) | 1187.224(1050.925,1349.676) | 119(98,146) | 608.039(504.832,744.21) | -1.564(-3.018,-0.088) |
| Niue | 30(25,37) | 1335.968(1099.191,1644.639) | 29(26,33) | 1869.405(1693.61,2132.076) | -0.150(-0.986,0.693) |
| Tokelau | 17(14,22) | 1160.817(924.902,1448.995) | 20(17,24) | 1573.175(1306.863,1867.451) | -0.294(-0.807,0.221) |

Abbreviations: DALYs, disability adjusted life years; EAPC, estimated annual percentage change; UI, uncertainty interval.

^a^ EAPC is expressed as 95% confidence interval.
