# Supplementary material for: Global, regional, national burden and trends of unintentional injuries from 1990 to 2021 and projections to 2035: a systematic analysis of the Global Burden of Disease study 2021
Source: Front Public Health. 2025 Sep 3;13:1653491. doi: 10.3389/fpubh.2025.1653491 (PMC12442766; doi:10.3389/fpubh.2025.1653491)
Supplement: Supplementary file 11 [file Supplementary_file_1.docx]

**Figure S1:** Global Trends in the Number and Percentage Change in Unintentional Injury Deaths in 204 Countries and Areas A. Number of Deaths in 1990 B. Number of Deaths in 2021 C. Percentage Change in Number of Deaths

**Figure S2:** Global trends in Age-Standardized death rates for unintentional injuries and estimated annual percentage changes for 204 countries and areas A. Age-Standardized death rates for 1990 B. Age-Standardized death rates for 2021 C. Estimated Annual Percentage Changes in death rates

**Figure S3:** Global Trends in the Number and Percentage Change in the Number of Unintentional Injury Disability-Adjusted Life Year (DALY) Cases in 204 Countries and Areas A. Number of DALYs in 1990 B. Number of DALYs in 2021 C. Percentage Change in DALYs Cases

**Figure S4:** Global Trends in Accidental Injury Age-Standardized Disability-Adjusted Life Year (DALY) Rates and Estimated Annual Percentage Changes for 204 Countries and Areas A. 1990 Age-Standardized DALYs Rates B. 2021 Age-Standardized DALYs Rates C. Estimated Annual Percentage Changes

**Figure S5:** Cutting-edge analysis, represented by the solid black line, exploring the relationship between the Sociodemographic Index (SDI) and the age-standardized rates (ASR) of incidence (A), deaths (B), and Disability-Adjusted Life Year (DALY) rates (C) for unintentional injuries

**Figure S6:** Projected unintentional injury deaths figures and ASR from 1990 to 2035 according to BAPC modeling

**Figure S7:** Projected unintentional injury disability-adjusted life year (DALY) rates and ASRs from 1990 to 2035 based on BAPC modeling

**Table S1:** Unintentional Injury Morbidity and ASRs per 100,000 cases, by 204 countries and territories, 1990 and 2021, and EAPC per 100,000 ASRs, 1990-2021

**Table S2:** Unintentional Injury Deaths and ASRs per 100,000 Cases, by 204 Countries and Territories, 1990 and 2021, and EAPC per 100,000 ASRs, 1990-2021

**Table S3:** Number of DALYs and ASRs per 100,000 unintentional injuries by 204 countries and territories, 1990 and 2021, and EAPC per 100,000 ASRs, 1990-2021
